# A sexual division of labour at the start of agriculture? A multi-proxy comparison through grave good stone tool technological and use-wear analysis

**DOI:** 10.1371/journal.pone.0249130

**Published:** 2021-04-14

**Authors:** Alba Masclans, Caroline Hamon, Christian Jeunesse, Penny Bickle

**Affiliations:** 1 Department of Archaeology and Anthropology, Institució Milà i Fontanals, Consejo Superior de Investigaciones Científicas, Barcelona, Spain; 2 UMR 8215 Trajectoires, French National Centre for Scientific Research, Nanterre, France; 3 MISHA 5, Université de Strasbourg, Strasbourg, France; 4 Department of Archaeology, University of York, York, United Kingdom; University at Buffalo - The State University of New York, UNITED STATES

## Abstract

This work demonstrates the importance of integrating sexual division of labour into the research of the transition to the Neolithic and its social implications. During the spread of the Neolithic in Europe, when migration led to the dispersal of domesticated plants and animals, novel tasks and tools, appear in the archaeological record. By examining the use-wear traces from over 400 stone tools from funerary contexts of the earliest Neolithic in central Europe we provide insights into what tasks could have been carried out by women and men. The results of this analysis are then examined for statistically significant correlations with the osteological, isotopic and other grave good data, informing on sexed-based differences in diet, mobility and symbolism. Our data demonstrate males were buried with stone tools used for woodwork, and butchery, hunting or interpersonal violence, while women with those for the working of animal skins, expanding the range of tasks known to have been carried out. The results also show variation along an east-west cline from Slovakia to eastern France, suggesting that the sexual division of labour (or at least its representation in death) changed as farming spread westwards.

## Introduction

Gender is acknowledged to be a produced, performed, and regulated cultural construct that can be materially documented and analysed [1, 2]. In turn, sexual division of labour and gendered task specialisation have been noted to be key issues for understanding social, political, and economic systems in the social sciences worldwide. This has been widely discussed through ethnographic research in hunting-gathering-fishing communities [see reviews in 3–6] as well as in agrarian societies [7–14].

The impact of a sexual division of labour in the formation of the first farming societies during the European Neolithic has been tackled from different perspectives producing little agreement about its significance in social organisation [15–23]. Despite the potential of archaeology to contribute to the history of sexual division of labour, using methods for identifying gendered activities that do not rely upon ethnographic and historical analogy research has been limited. The variabilities in cultural and economic practices across the European Neolithic have made it increasingly difficult to accept universal models of gender during the transition to agriculture [22, 24].

In view of the above, this paper aims to make a contribution to better understanding the processes by which women, men and other potential genders came to be practically and ideologically associated with certain tasks and activities during the *Linearbandkeramik* period in Central European Early Neolithic (*LBK*; ca. 5,500–5000 cal BC). The LBK burial record will be our main target to tackle this question, aiming to go beyond the analysis of the funerary sphere as a straight-forward presentation of the two sexes at death, by considering the relationship between the tasks carried out in life and the ways in which they were represented at death for the *LBK*.

Gender-based lifeways, taskways and symbolism during the spread of farming in Central Europe have been approached from different methodological and interpretative frameworks. It has been noted that population growth entailed decreased spacing of births and increasing time spent on nursing and childcare [25–27]. Strontium isotope ratios suggest gender-biased mobility patterns in some areas of the LBK [28–30] and dietary isotopic analysis indicate that males and females may have had slight differences in their diets [29]. Osteological and physical activity data strongly suggest sex differentiated activity patterns in upper and lower limbs muscular-skeletal stress markers and labour-related asymmetry between the sexes [23, 31–33]. Despite this rich data record, studies on gendered lifeways are still rather isolated and limited to a few sites [24].

*LBK* funerary customs mostly include large cemeteries and isolated tombs within settlements, where graves are generally oval, dug directly in the ground, and the bodies inhumed individually [34]. The grave goods generally consist of ornaments, polished and bevelled artefacts (PBAs from henceforth), flint blades, pottery vessels, pebbles possibly used as utensils, bone tools, antler items and ochre, as well as a group of objects generally referred to as ‘grinding tools’. Previous analysis of the grave good assemblage variation by osteological sex and age has led many researchers to argue that women and children were of lower status than men [34–41], though others have argued these interpretations were based on modern assumptions of binary gender hierarchies and values, rather than established directly from the evidence [41–43]. Whether the variation in grave goods between male and female burials also extended to tasks in life remains to be determined.

Grave goods have been considered to be representative of the buried individuals’ former belongings, gifts from mourners, equipment for the afterlife, or as symbolic representation of the activities related to the identity of the deceased, and, as such, are subject to political or ideological manipulation by mourners [44, 45]. Thus, while grave goods may not represent the identity of the deceased in a one-to-one relationship [for further discussion see 46, 47], they provide a powerful insight into the role of material culture in identity formation. Grave goods have also been shown to be active in creating identity through their powerful symbolic role in death rites [48], and thus may have shifting meanings over time. We recognise that while in an individual case, the gender of the deceased may not be captured directly by the grave goods, or may have been actively denied by mourners, analysis at a broadscale will reveal which activities were recurrently associated with sex, gender, age, or other hierarchies.

Artefact analysis [43, 49, 50], as well as interpretations based on the prevalence of grave goods [29, 34, 51, 52], have suggested that sexed-based differences were being symbolically represented in funerary assemblages, including those related to labour. The tasks represented in LBK grave goods have started to be challenged thanks to the development of use-wear analysis [43, 50], with new proposals suggesting that items such as stone adzes were being used not only in woodworking activities but also in other tasks.

Our research aims to contribute to a greater understanding of this topic through use-wear analysis of lithic tools. Here we analyse for the first time how 441 stone tools were used before they became grave goods. Use-wear analysis, the microscopic examination of tool surfaces, in combination with experimental data, can assess what types of material a tool was brought into contact with during its use-life [53, 54].

**Fig 1:**
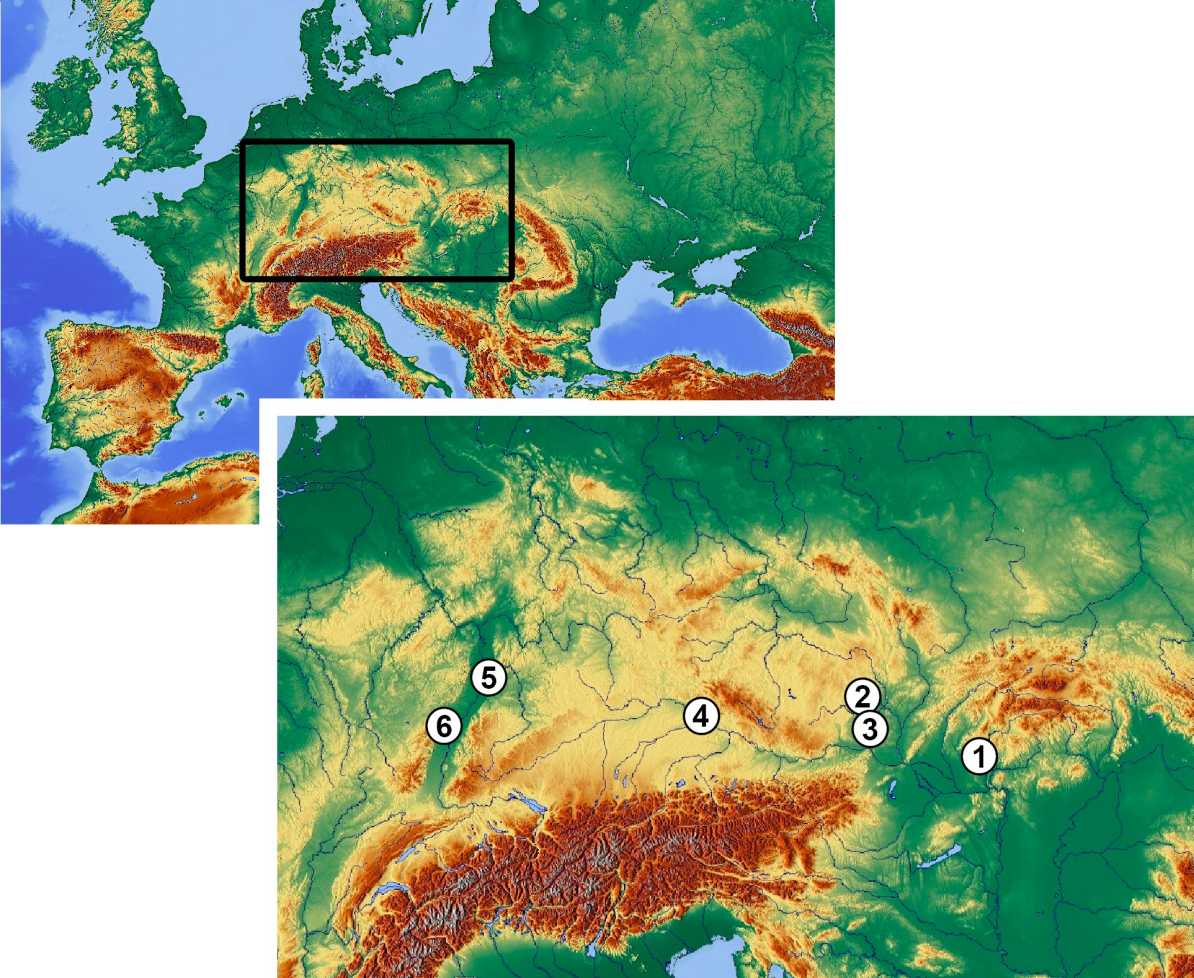
Location of the studied sites. 1. Nitra, 2. Vedrovice, 3. Kleinhadersdorf, 4. Aiterhofen, 5. Schwetzingen, 6. Vendenheim.

The sample selection comes from inhumations from six large cemeteries across central Europe: Vedrovice [Czech Republic; 55], Nitra [Slovakia; 35], Kleinhadersdorf [Austria; 56], Aiterhofen [Germany; 38], Schwetzingen [Germany; 57, 58], and Vendenheim [France; 59] (Fig 1). The chronological span of these cemeteries is 150 years, between 5315–5224 cal BC (95% probability) [Vedrovice initial occupation span according to 43 on the basis of 60] and *c*.5100 cal BC [*moyen-récent* LBK pottery phases at Vendenheim, a period radiocarbon dated by 61], thus allowing a broadly contemporary snapshot of early Neolithic burial practices.

## Objectives

The main aim of the present research is to compare and interpret how biological sex could have been experienced in life, in the activities carried out, with how it was represented at death. Overall, we want to assess whether the data stood up against the suggestion that there had been a sexual division of labour in the Neolithic and, going beyond this, investigate what tasks that may have entailed.

To that end, we established the following specific objectives: 1) to identify grave goods’ stone tool use and technological characteristics through use-wear and metrical analysis; 2) to assess whether there is correlation between stone tool functional and technological data and the inhumated individuals’ sex provided by previous osteological reports [35, 38, 55–58, 62]; 3) to examine whether functional and technological differences co-vary with other potential markers of identity, such as mobility, diet, and funerary practices, as well as geographic variation in an European east-west axis.

In the text, ‘gender’ will be used to refer to the cultural attributes and ‘sex’ to the predominant biological differences, as a starting point from which to determine how strongly the latter is expressed in the former.

## Materials and methods

### Materials

*Linearbandkeramik* funerary customs varied, encompassing large cemeteries, isolated graves associated with houses and settlements, various instances of disarticulated human remains from settlements, enclosures and caves, and some rarer instances of mass burials after episodes of violence [34, 63, 64]. Single inhumation at cemeteries accounts for the largest percentage of known burials [34, 62].

We selected our sample with the following factors in mind: 1) geographic representativity from the distribution of the LBK; 2) reliability of the contexts: where spatial and temporal information was available, detailed and well attested through radiocarbon dating; 3) availability of lifeways data from specific analyses results δ^13^C, δ^15^N, δ^87^Sr/^86^Sr isotope analyses has been taken into account; 4) bone preservation and availability of osteological studies; 5) cemeteries of more than 30 individuals have been sought in order to be able to perform statistically significant analysis.

This study is comprised of six cemeteries, from a transect across the southern distribution of the LBK, from Slovakia to Alsace. This amounted to 621 well preserved inhumations, belonging to 151 female-sexed skeletons, 36 probably female-sexed skeletons, 107 unsexed individuals, 137 males, 36 probably male-sexed skeletons and 154 non-adults (see Table 1). We drew on the sex estimations from the published literature, and all the information related to osteological criteria can be consulted in detail in the corresponding publications [35, 38, 55–58, 62]. Nitra [65] and Stuttgart-Mühlhausen [66] are currently being studied for aDNA analysis and we acknowledge sex determinations may change in the future.

**Table 1:** Age and sex ranges of the individuals.

Site	Age	Female	Male	Indeterminable
*Vedrovice*	Non adult			21
	Juvenile/ Adult	41	21	3
*Nitra*	Non adult			19
	Juvenile/ Adult	26	19	7
*Kleinhadersdorf*	Non adult			16
	Juvenile/ Adult	11	14	5
*Aiterhofen*	Non adult			18
	Juvenile/ Adult	43	54	21
*Schwetzingen*	Non adult			58
	Juvenile/ Adult	63	52	12
*Vendenheim*	Non adult			22
	Juvenile/ Adult	3	13	59

Non adult (from birth to 12 years old), Juvenile/Adults (from 12 to more than 50 years old). Specific age details according to [67] can be consulted at Table 1 in S2 File.

An assemblage of 146 polished and bevelled artefacts (stone adzes, axes, scrapers, named PBAs hereafter), 173 projectiles, 102 flaked tools and 20 pebbles/maces/discs were assessed through surface microscope analysis (Table 2, Tables 2–5 in S2 File).

**Table 2:** Studied lithic tools count according to the site.

Site	PBAs	projectiles	flaked tools	pebbles/maces/discs
*Aiterhofen*	54	50	40	1
*Kleinhadersdorf*	12	14	7	0
*Nitra*	10	0	8	0
*Schwetzingen*	17	35	21	8
*Vedrovice*	19	39	9	2
*Vendenheim*	34	35	17	9

The data for our multi-proxy approach was taken from published sources. The isotope data was collated from the “Lifeways project” [62], as well as from several publications on the Vedrovice cemetery [68–71], alongside the grave good data (Table 1 in S2 File). Two principal sets of isotope data were used, the dietary isotopes of carbon and nitrogen and strontium isotope ratios. The methods and quality data for the isotope data used in this paper are reported in full in 62. The database produced for this isotope analysis is available on request from penny.bickle@york.ac.uk.

### Methods

During the use-wear analysis the PBA, quartzite, quartz and flint experimental reference collection was kindly provided by IMF-CSIC’s Laboratory of Prehistoric Technology (Barcelona) (available on request from asd@imf.csic.es). The identification and interpretation of wear traces on PBAs and macrolithic tools were made in accordance with established methods [72–78], while the flaked tools were studied following the standards proposed by [53, 79–82] for the projectile wear traces.

The use-wear analysis was performed using a reflected-light electron microscope (Olympus BH2, 50× to 200× magnification) and a binocular microscope (Olympus BX51TRF, 10× and 40× magnification). The image procurement was achieved by means of a Canon EOS1100D camera and multifocal assemblage using Helicon Focus software. The cleaning procedures included washing the artefacts with soap and water. In those cases where it was necessary to eliminate concretion of calcareous nature, the artefact was left for at least 5 minutes in the ultrasonic machine submerged in a 5% aqueous NaCl solution. This procedure was repeated until the complete disappearance of the residue.

PBAs were also subjected to further description through measurement of size and weight, which were then assessed against existing typologies [83]. Technological description of the PBA was performed according to established methods [84, 85].

The comparison to the burial data was performed through multi-level modelling, through MCA [86] to identify correlations between different datasets, first, the skeletal and then the isotopic. MCA statistics has been performed using “R” software (version 4.0.1.) (see S3 File for details).

## Stone tool technological results

### PBA technological characteristics

Of the 146 PBAs included in this study, 144 were sufficiently preserved to perform technological analysis (Table 2 in S2 File). 95% of those items were whole artefacts, whereas in 8 cases they were partial, having been fragmented before burial. The PBA’s technical features were very standardised without proximal fractures, completely polished and displaying both technological extraction presence (flaking rough outs) and fine finishes (Table 3).

**Table 3:** PBA’s technical features.

Site	Extractions			Tech polish		Proximal fracture		
	absence	ind	presence	total	ind	absence	presence	ind
*Vedrovice*	1		18	19		14	5	
*Nitra*			7	7		7		
*Kleinhadersdorf*	4		7	11		7	4	
*Aiterhofen*	7	3	40	48	1	42	3	5
*Schwetzingen*	6	3	8	15	2	1		2
*Vendenheim*	7	2	23	30	2	27	1	2
**Total**	25	8	**103**	**130**	5	**98**	13	9

Ind = indetermined.

**Fig 2:**
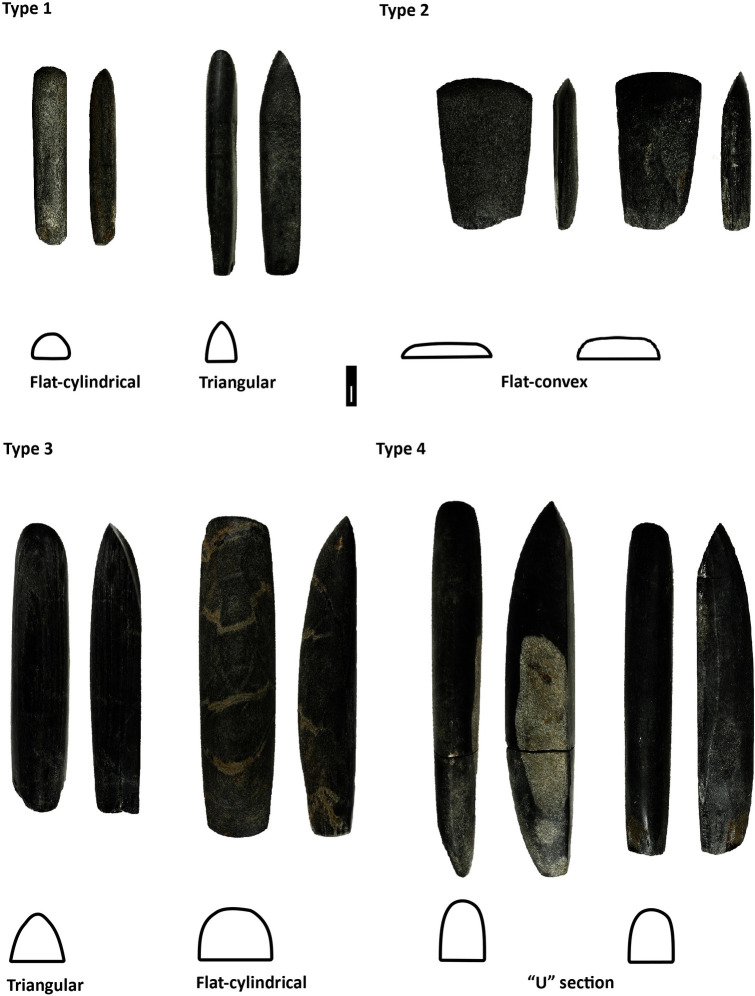
Polished and bevelled artefact types and sections examples [43]. Types 1–4 corresponding to Ramminger’s PBA classification [83].

**Fig 3:**
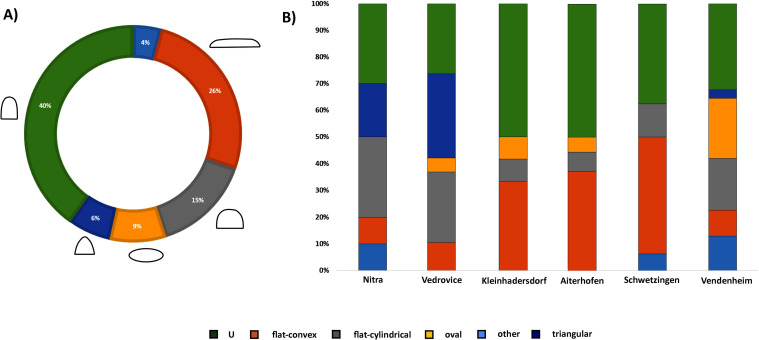
A) PBA sections general percentage; B) PBA section proportions according to the sites.

Morphological descriptions of the PBAs were based on their section, length, and weight (Table 2 in S2 File). Sections were classified as oval, flat-convex, flat-cylindrical, triangular, “u”-shaped and “other” (irregular, hexagonal, quadrangular) (Fig 2). “U”-shaped and flat-cylindrical were the most frequent in the sample, though significant differences between sites and regions can be observed (Fig 3) (Table 1 in S1 File). Triangular and flat-cylindrical features are more often found in the most eastern (Vedrovice, Nitra) and western (Vendenheim) sites, whereas flat-convex and “u”-shaped were dominant in the central German and Austrian sites (Aiterhofen, Kleinhadersdorf and Schwetzingen).

**Fig 4:**
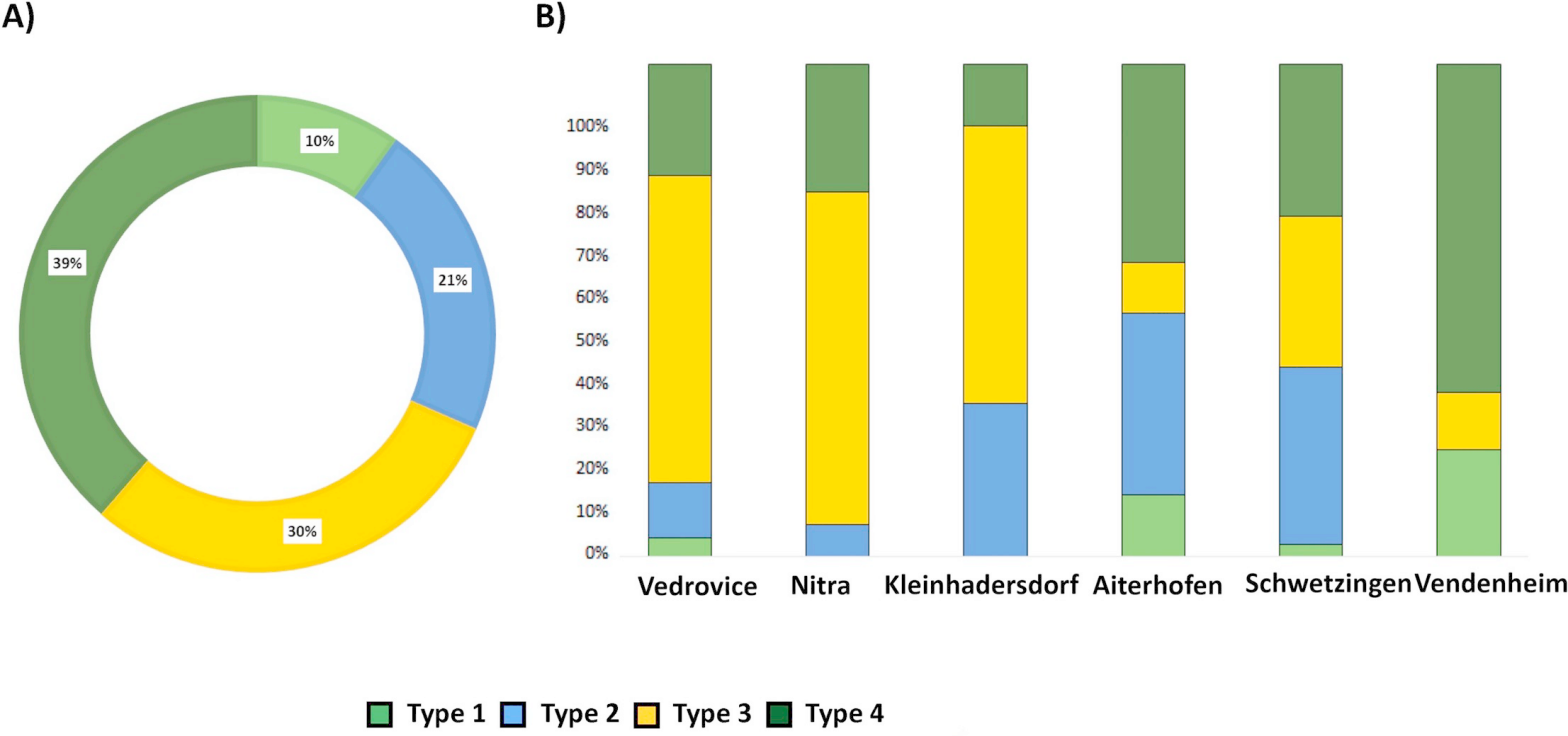
A) PBA types general percentage; B) PBA types proportions according to the sites.

Ramminger’s typological classification was used for analysis, namely HBI/width correlation, HBI being the “height-breadth-index” calculated as (thickness/width)*100 [83] (Fig 2). Types 3 and 4 are the most abundant among the selected sample, though, again, their proportion significantly varies depending on the site and regional distribution (Table 1 in S1 File). Type 3 is dominant among eastern LBK cemeteries (Vedrovice, Nitra and Kleinhadersdorf). At Aiterhofen, Schwetzingen and Vendenheim there is more typological variability, with types 2 and 4 the most frequent (Table 4, Fig 4).

**Table 4:** PBA’s HBI types count according to the sites.

Site/ HBI Type	Type 1	Type 2	Type 3	Type 4
*Vedrovice*	2	6	**33**	12
*Nitra*	0	2	**21**	8
*Kleinhadersdorf*	0	10	**18**	4
*Aiterhofen*	15	**44**	12	**48**
*Schwetzingen*	1	**14**	**12**	12
*Vendenheim*	17	0	9	**52**
Total	35	76	105	136

**Fig 5:**
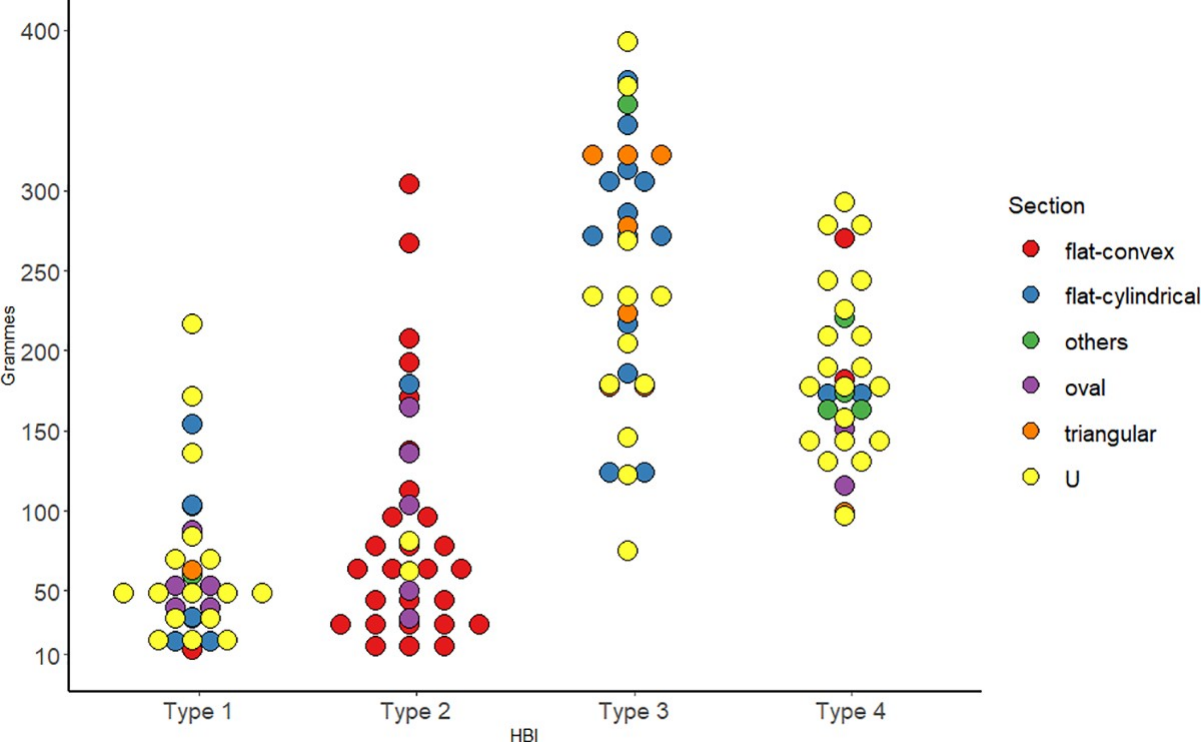
Dot plot including the PBA’s weight values and HBI types according to their sections.

Fig 5 illustrates the relationship between the variables weight (in grammes), HBI type and section (Table 2 in S1 File). Type 1 are the lighter PBAs (between 10 and 60 g) including oval, “u”-shaped and flat cylindrical sections. Type 2, strongly related to flat-convex, oval sections, and light weights (between 10 and 150 g). Types 3 and 4 contain the heavier tools (between 100 and 400 g). In the first case, two groups can be identified: a mixture of flat-cylindrical and “u”-shaped items between 100 and 230 g, and another between 130 and 400 g, predominantly formed by triangular and flat-cylindrical. Finally, type 4 is mainly represented by “u”-shaped tools.

### Flaked tools and projectiles technological characteristics

A total of 173 projectiles and another 102 flaked tools were targeted in this study, mainly made of chert and, to a lesser extent, radiolarite or jasper. Of those, it was possible to perform technological analysis on 99 flaked items and 169 projectiles (Tables 3 and 4 in S2 File).

**Fig 6:**
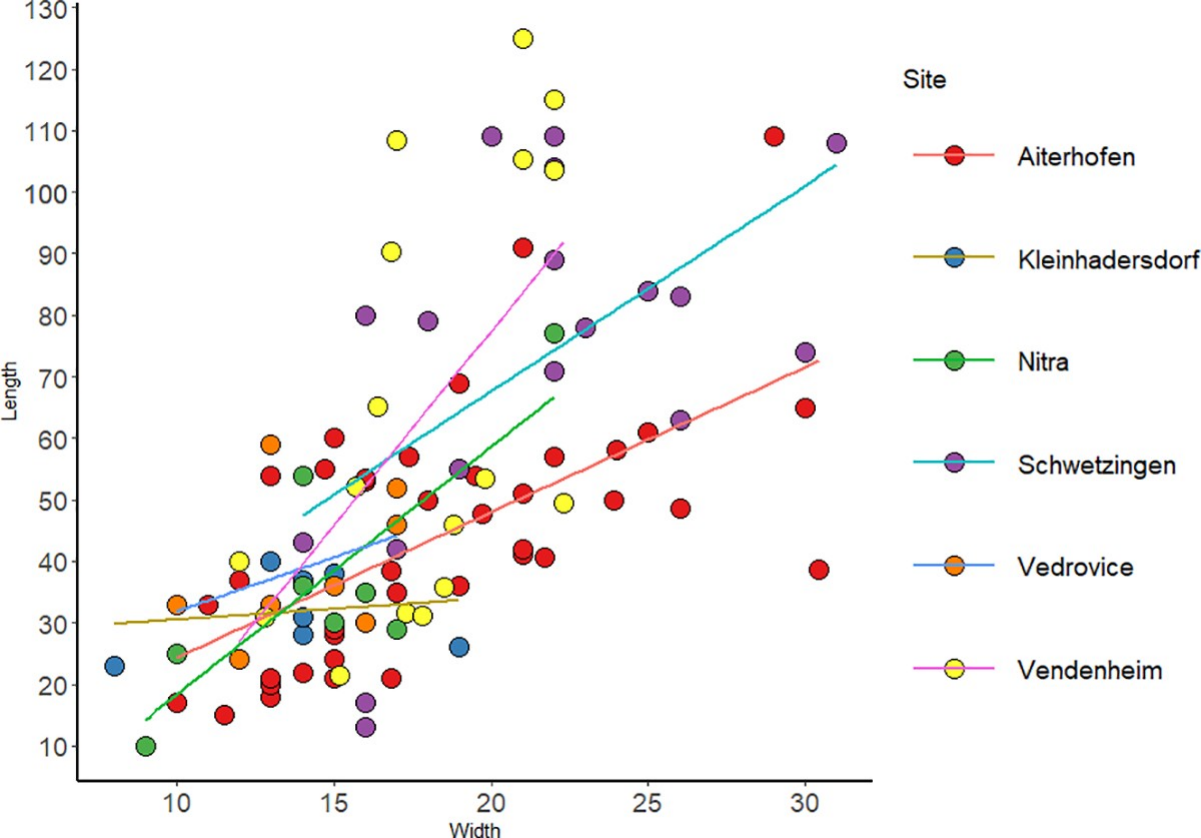
Flaked tools measurements (length/width) according to the site.

Flaked tools mainly correspond to blades/blade fragments (75%). Significant differences were identified between the different sites’ tool measurements (Table 1 in S1 File). Eastern sites (Vedrovice, Nitra and Kleinhadersdorf) presented the most similar values, displaying low variability (between 10–77 mm length and 8–22 mm width) in comparison with the western sites and Aiterhofen (Fig 6). No difference between the sites has been noted in the quantity of flaked tools (Table 1 in S1 File).

**Fig 7:**
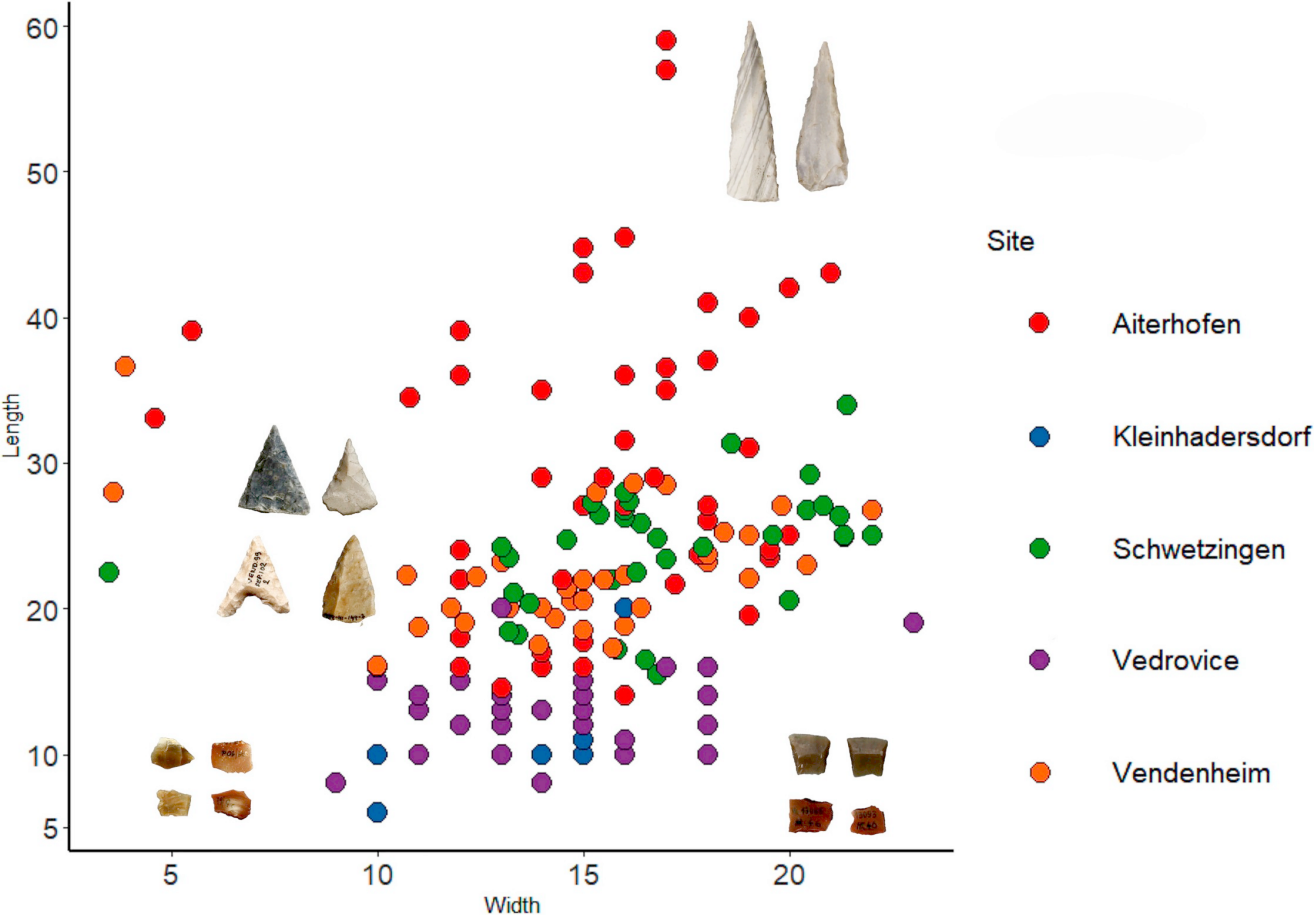
Projectile measurements (length/width) according to the site.

There was a strong east-west variation in morphological characteristics and metrics of the projectiles (mainly blade fragments) assemblages (Table 1 in S1 File). Projectile points from western sites (Schwetzingen and Vendenheim) and Aiterhofen were generally asymmetric and symmetric and triangular in shape, retouched and displaying high elongation indexes (ratio length/width): Aiterhofen (mean elongation index 2.08), Schwetzingen and Vendenheim (mean elongation indexes 1.6 and 1.9 respectively). In contrast, trapezoidal tips with or without retouch were common in the east, characterised by lower elongation indexes (0.9 at Vedrovice and Kleinhadersdorf) (Fig 7).

An increasing number of projectiles in graves was found from east to west, with statistically significant differences between sites. Eastern cemeteries presented projectile ratios between 0 to 13%, while at the western sites and Aiterhofen, the projectile presence is slightly higher (Table 5). It is worth noting that at Schwetzingen projectile points were developed by means retouched bone (there were 30 bone arrowpoints in 15 burials, corresponding to 7.6% of the burials) as well as of flaked stone (7.6% of the burials), accounting for 13.5% projectiles.

**Table 5:** Lithic projectile count and percentages according to the cemeteries.

	Vedrovice	Kleinhadersdorf	Nitra	Aiterhofen	Schwetzingen	Vendenheim
*Burials with projectiles*	8	6	0	17	14	17
*Burials without projectiles*	78	40	71	119	171	80
*Total burials*	86	46	71	136	185	97
*% of burials with projectiles*	9%	13%	0	13%	8%	18%

## Results of the use-wear analysis

### PBA

Out of the total number of 146 artefacts included in the study, 21 were not suitable for use-wear analysis because of the poor preservation of their surface areas and in a further 18 cases only the morphological data was available. As a result, the analysis has been performed on 107 items (Table 2 in S2 File).

The use-wear results indicate, for the first time with use-wear analysis, a broad spectrum of PBA uses including woodwork, contact with hide/leather, bone and meat. Wood-working activities and contact with meat and bone through direct percussion (broadly defined as butchering) were the most frequent uses (Table 6). PBAs were also employed against soft matters (possibly animal-related) and hide-working tasks, though less frequently (Table 6). A significant number of tools (28%) were used in direct percussion activities against hard materials but did not present enough evidence to determine the specific contact matter. In these cases, the macroscopic traces observed on the active surface areas indicated the presence of contact with hard materials, so we know that they were used, but no comparisons could be determined from the reference collection.

**Table 6:** Use-wear analysis on PBAs tools results according to the worked materials.

PBA uses	Total tools	% *
butchering	15	**16**
Animal soft tissues?	8	9
hard material	26	**28**
hide-work	5	**5**
Not analysable	26	
not studied	19	
not used?	12	13
Soft matter	6	**6**
used tool (use indeterminable)	7	
woodwork	15	**16**
woodwork?	7	7
Total	146	

* Percentage calculated based on the assemblage with determinable use (excluding those with indeterminable use, not analysable and not studied items).

In 12 cases the artefact active surfaces were apparently unaltered. In those circumstances three possible interpretations arise: either they were unused (that is to say, made *ex profeso* to be buried), intentionally sharpened previously to their deposition in the graves [as has been interpreted in other contexts by 87 and 78], or they were used for such a short time that no wear developed.

**Fig 8:**
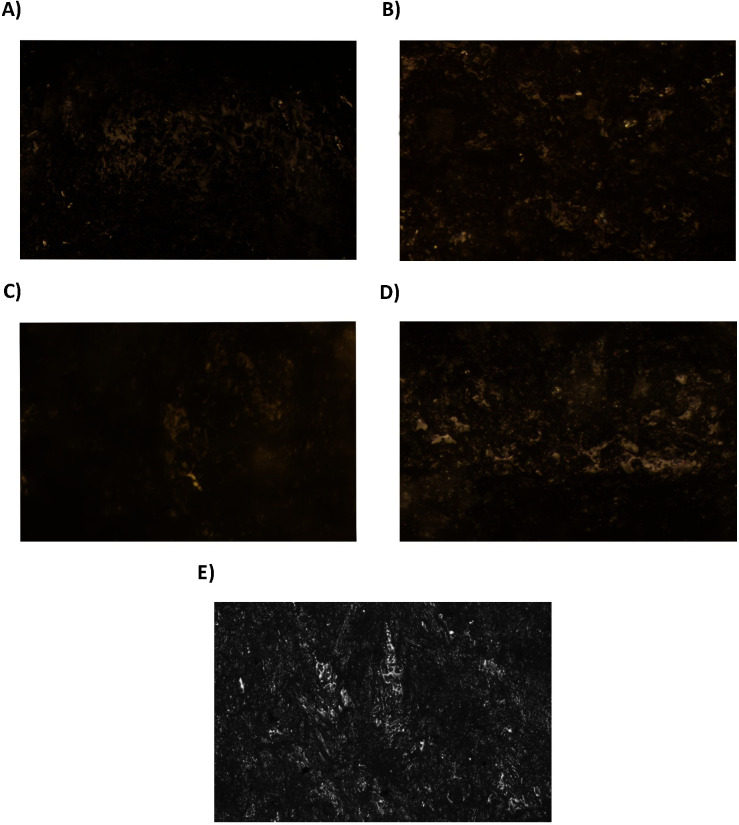
Woodworking tools. A) Burial 28 (Aiterhofen) 200x; B) Burial 6 (Schwetzingen) 200x; C) Burial 148 (Vendenheim), 200x; D) Burial 25 (Nitra), 200x; E) Burial 17 (Vedrovice), 400x.

Fifteen tools display evidence of woodworking (Table 7) and seven more of provable use on woodwork through direct percussion. Wood-working traces consist of isolated, compact, and very shiny micro polishes of undulating micro-topography (Fig 8). Sporadically there are also abrupt, isolated, and fresh micro-chipping in tools which indicate continual exposure to a percussive activity against a hard material. The wear development on these artefacts indicates transversal hafting to a handle, where one of the two bevels is more affected by micro-chipping damage.

**Table 7:** PBAs displaying evidence of woodworking count and characteristics.

Site	Burial	Code	Section	Grammes	HBI Type
**Aiterhofen**	grab56	56.b	flat-convex	32	2
**Aiterhofen**	grab29	29	U	66	1
**Aiterhofen**	grab28	28.e	U	81	2
**Aiterhofen**	grab10	10.b	flat-convex	92	2
**Aiterhofen**	grab25	25.a	U	145	4
**Aiterhofen**	grab139	139	U	188	4
**Aiterhofen**	grab10	10.a	U	215	4
**Nitra**	8	8	flat-cylindrical	286	3
**Nitra**	25	25	flat-cylindrical	369	3
**Nitra**	58	58	hexagon	354	3
**Schwetzingen**	stz-220	220	U	283	4
**Schwetzingen**	stz-006	006	flat-convex	171	2
**Vedrovice**	15/75	13015	U	554	3
**Vendenheim**	151	151.2	flat-convex	87	1
**Vendenheim**	148	148.7	U	97	4

The PBA morpho-technical characteristics suggest their use on several kinds of woodworking activities, which could have included tree felling, cutting firewood and various types of carpentry from building houses to small object carving. This matches the expectations from tool marks suggesting PBA use, found on preserved wood from the LBK, e.g. in water wells [88, 89]. Carpentry tasks, as well as other wood-related crafts, have been widely recognised to have been performed using PBAs, although more use-wear studies on wood [such as those of 90] are necessary in order to refine knowledge of the woodworking techniques employed.

**Fig 9:**
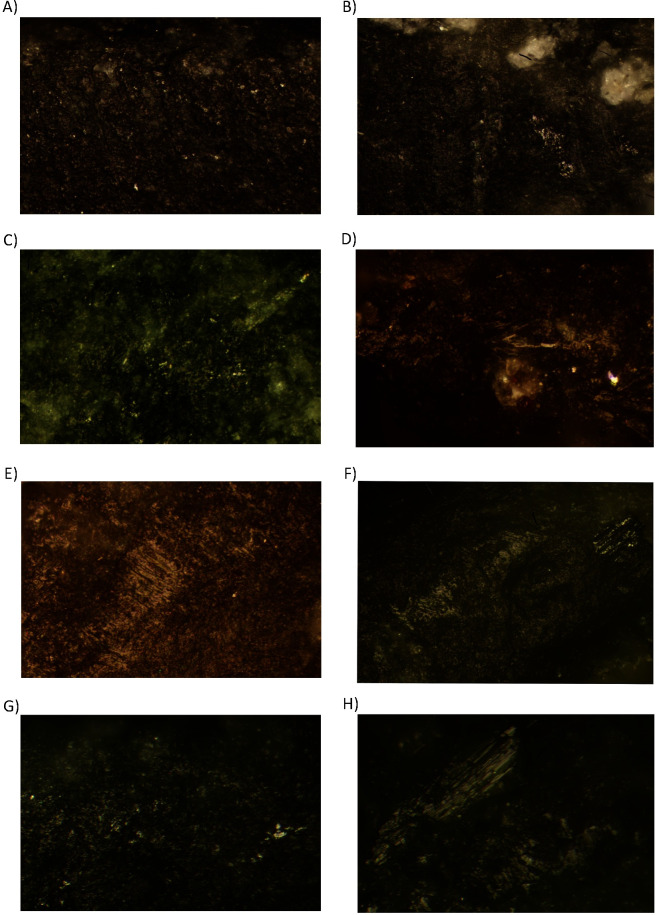
Butchering and soft animal tissue tools. A) Burial 22 (Aiterhofen) 200x; B) Burial 153a (Aiterhofen) 200x; C) Burial 67 (Kleinhadersdorf), 200x; D) Grab 21 (Nitra), 200x; E) Grab 21 (Nitra), 200x; F) Burial 172.3 (Vendenheim), 200x; G) Burial 19/75 (Vedrovice), 200x; H) Burial 69/78 (Vedrovice), 200x.

Fifteen tools display evidence of contact with bone and/or meat (Table 8), and 8 more, of provable use on soft animal tissues (Table 9). A slight rounding along most of the tool’s edge with open/semi-open micro-polishes of irregular microtopography on the matrix was documented and related to contact with fresh meat or other soft animal tissues (Fig 9). In some cases, these traces appeared combined with sporadic fresh surfaces and small spots of a shiny compact directional micro-polish, possibly indicating contact with a hard and moist material such as bone (Fig 9).

**Table 8:** PBAs displaying evidence of bone and meat count and characteristics.

Site	Burial	Code	Section	Grammes	HBI Type
**Aiterhofen**	grab2	2.5	flat-convex	193	2
**Kleinhadersdorf**	40	40.3	flat-convex	208	2
**Kleinhadersdorf**	90	90.3	U	239	3
**Nitra**	21	21	triangular	278	3
**Nitra**	34	34	triangular	327	3
**Vedrovice**	57/78	13126	U	191	4
**Vedrovice**	69/78	13146	flat-cylindrical	278	3
**Vedrovice**	19/75	13025	flat-cylindrical	313	3
**Vedrovice**	77/79	13175	triangular	318	3
**Vedrovice**	46/77	13085	triangular	321	3
**Vedrovice**	59/78	13190	flat-cylindrical	341	3
**Vedrovice**	54/78	13114	U	393	3
**Vendenheim**	172	172.3	U	184	4
**Vendenheim**	20	20.1	flat-cylindrical	154	1
**Vendenheim**	94	94.2	U	175	4

**Table 9:** PBAs displaying evidence of soft animal tissues count and characteristics.

Site	Burial	Code	Section	Grammes	HBI Type	WMC	Kinematics
**Aiterhofen**	grab153	153.a	flat-convex	101	2	1) soft 2) hard material	direct percussion
**Aiterhofen**	grab153	153.b	U	172	4	soft	direct percussion
**Aiterhofen**	grab41	41.b	U	74	1	soft	indeterminable
**Kleinhadersdorf**	g3	62218	oval	165	2	(1) soft + (2) hard	direct percussion
**Kleinhadersdorf**	67–2	9	U	229	3	soft	direct percussion
**Schwetzingen**	stz-133	133	U	179	3	soft	direct percussion
**Vedrovice**	71/79	13163	triangular	183	3	soft	direct percussion
**Vedrovice**	31/76	13047	flat-convex	70	2	soft	indeterminable

WMC = worked materials general characteristics.

Bone and meat damage patterns could be interpreted either as resulting from interpersonal violence or animal butchering. Butchery of animals in the course of food production seems much the more probable of the two, due to the high occurrence of animal bone on settlement sites. Archaeozoological evidence of using PBAs in butchering activities in Europe is presently scarce possibly due to the lack of specific studies concerning the identification of animal butchering marks. To resolve this, future studies could apply use-wear studies to animal bones, such as those carried out by [91, 92] in Southern France Final Neolithic contexts, where the presence of PBA impact traces on animal bones was documented.

The use of PBAs in interpersonal violence, however, is attested, especially in later phases of the LBK where they have been shown to be repeatedly used in the course of human massacres as well as to inflict torture and mutilation [93–97]. Palaeopathological studies on a more circumspect number of LBK individuals suggest that almost 20% from the analysed population was affected by physical interpersonal violence [98]. Thus, PBAs may have also served as temporary weapons to inflict violence on human bodies. From this use, we hypothesis that PBAs may have held a symbolic power of this use in conflict.

**Fig 10:**
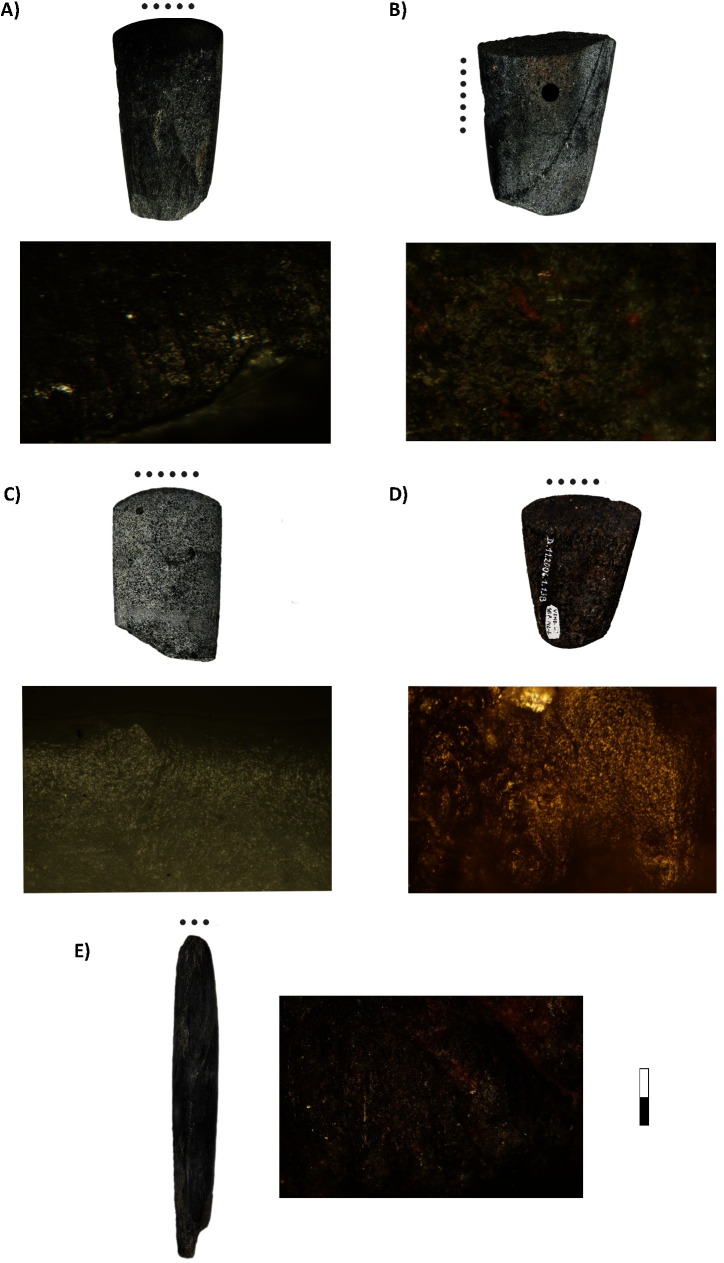
Hide-working tools. A) Burial 18 (Vedrovice); 100x; B) Burial 36 (Vedrovice), 200x; C) Burial 133 (Vendenheim), 100x; D) Burial 142 (Vendenheim), 100x; E) Burial 141 (Aiterhofen) 100x.

Hide-working traces were identified on 5 PBAs (Table 10). The use-wear on these artefacts is characterised by high rounding on the central part of the edge covered by an irregular semi-open to closed micro-polish that becomes closed on the upper parts of the topography (Fig 10). This phenomenon always appears more developed on one edge than the other, signalling one-bevelled scraping. In some cases, U-shaped in cross section, deep striations perpendicular to the edge are formed and covered with directional semi-open/semi-closed micro-polish of irregular micro-topography (Fig 10A, 10D and 10E).

**Table 10:** PBAs displaying evidence of hide-working count and characteristics.

Site	Burial	Code	Section	Grammes	HBI Type	Worked mat gen charact	Kinematics
**Aiterhofen**	grab141		U	84	1	1) hard + 2) flexible and abrasive	1) percussion + 2) abrasion
**Vedrovice**	36/76	13057	oval	104	2	soft flexible	scrape
**Vedrovice**	18/75	13021	flat-convex	70	2	abrasive flexible	scrape
**Vendenheim**	142	142.2	oval	38	1	soft flexible abrasive	scrape
**Vendenheim**	133a	133a	oval	52	1	abrasive	scrape

Skin-work by means of flint and macro-lithic tools has been consistently demonstrated in LBK [99, 100], though the absence of other PBA functional studies prevents us from making comparisons in use of this tool in this type of activity. The ethnographic record, however, provides interesting examples of skins being processed with the aid of these instruments such as certain indigenous communities from Alaska, Australia and the European circumpolar Arctic [101, 102].

**Fig 11:**
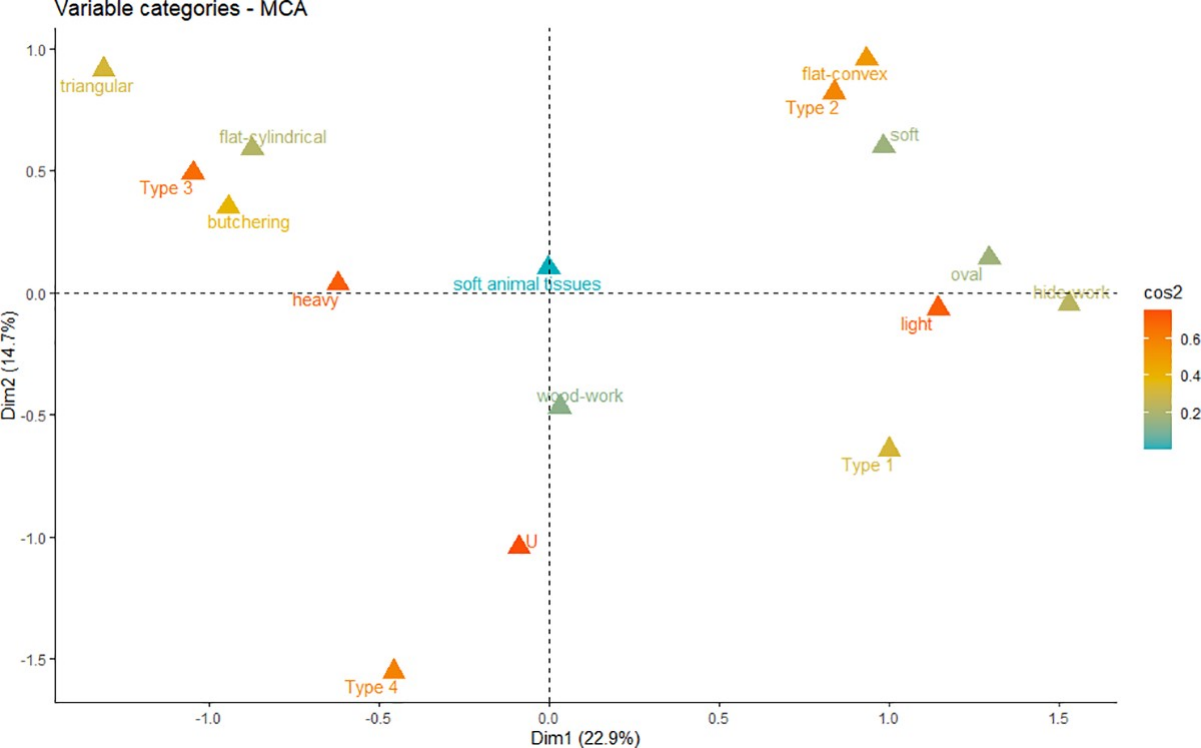
Multiple Correspondence Analysis displaying the relationships between PBAs morphological characteristics (sections, types, and weights) and their uses.

A Multiple Correspondence Analysis (MCA; Text 1 in S3 File) confirmed that PBAs morphological characteristics are strongly related to their uses (Table 2 in S1 File) (Fig 11), especially in two cases: hide-working and butchering tools. Hide-working and soft-matter working artefacts are generally associated to light oval and flat-convex-section tools belonging to HBI Type 2. In the case of Type 3 tools, they often correlated with triangular and flat-cylindrical sections, high weights and contact with meat and bone. Woodwork is located in the lower central part of the graph, as this activity tends to be identified among PBAs with Types 3 and 4 and rather heavy weights, though there are some cases from Aiterhofen and Vendenheim displaying lower weights and flat-convex sections.

A substantial geographical shift from the bigger and more standardised eastern tools of Vedrovice, Nitra and Kleinhadersdorf to the heterogeneous assembles in Aiterhofen and the western sites has been observed. Some variation was also observed between the different site’s PBA uses, though they were not statistically significant (Table 11) (Table 2 in S1 File). This variability may be related to changes and differences in the PBAs funerary symbolism, though more research is needed on PBAs from domestic contexts to confirm this pattern.

**Table 11:** Use-wear analysis on PBAs results according to the site.

Site/activity	butchering	hide-work	soft animal tissues	woodwork	hard material	soft
*Nitra*	2	0	0	2	1	0
*Vedrovice*	7	2	2	3	2	1
*Kleinhadersdorf*	2	0	2	0	6	0
*Aiterhofen*	1	1	3	10	7	3
*Schwetzingen*	0	0	1	2	3	2
*Vendenheim*	3	2	0	3	5	0

### Flaked tools and projectiles

**Fig 12:**
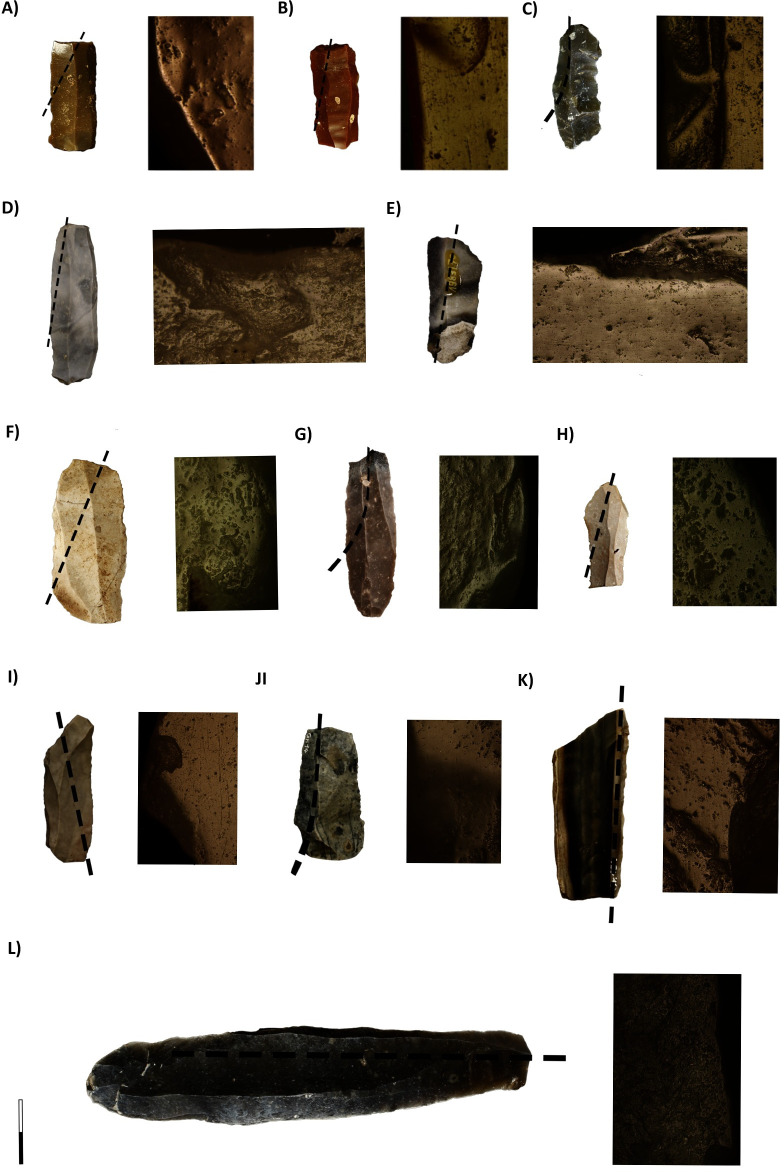
Harvesting tools. A) Burial 58 (Nitra), 10 and 200x, respectively; B) Burial 17.5 (Kleinhadersdorf), 10 and 200x, respectively; C) Burial G.1c (Kleinhadersdorf), 10 and 200x, respectively; D) Burial 10 (Aiterhofen), 200x; E) Burial 93 (Aiterhofen) 100x; F) Burial 90 (Vendenheim), 100x; G) Burial 137 (Vendenheim), 100x; H) Burial 148 (Vendenheim), 200x; I) Burial 21 (Schwetzingen), 100x; Burial 14 (Schwetzingen), 200x; Burial 154 (Schwetzingen), 200x; Burial 73 (Schwetzingen), 100x.

**Fig 13:**
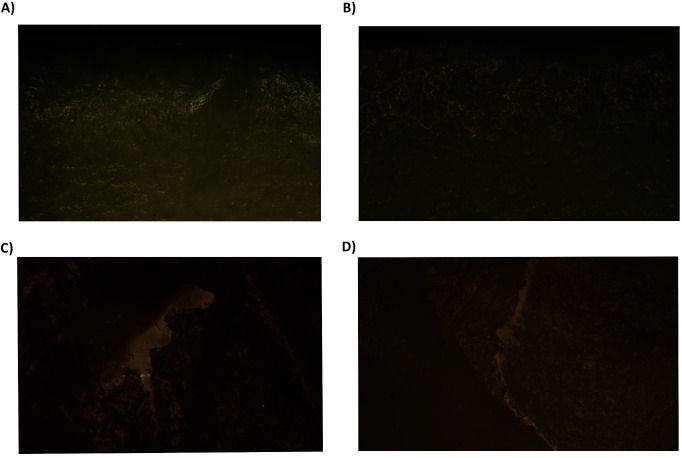
Flaked tools animal-related activities. A) Hide work (Vendenheim Burial 141, 200x); B) Hide work (Vendenheim Burial 165, 100x); C) Bone contact (Aiterhofen, Burial 153, 200x); D) Woodwork (Aiterhofen, Burial 55, 200x).

Use-wear analysis on flaked tools in the *LBK* has been rather sporadic [80, 99, 103–106] considering the size the flaked tools assemblages found on settlements. These studies suggest that hide-working activities were always dominant, with woodworking and harvesting accounting for only 10% of the assemblages, and bone/antler and soft materials were rather rare. Presently, no studies have focused on flaked lithic tools from burials. Our analysis concluded that these items, blades and some flakes, were mainly used as sickle blades in harvesting activities, although hide-work was also well represented (Figs 12 and 13) (Table 12, Table 3 in S2 File).

**Table 12:** Use-wear analysis on flaked tools results according to the worked materials.

Flaked uses	total tools	%*
animal longitudinal	3	5
animal?	1	
bone/antler	3	5
butchering	1	2
harvest	16	**26**
hide-work	8	**13**
hide-work?	1	
indeterminable	24	
not studied	11	
Not used	16	26
Not used?	14	23
woodwork?	4	
Total	102	

* Percentage calculated based on the assemblage with determinable use (excluding those with indeterminable use, not analysable and not studied items).

At Nitra and Kleinhadersdorf, localised patches of wear traces indicate that the sickle blades were diagonally hafted, suggesting a curved sickle with a serrated cutting-edge (Fig 12A–12C). One of the active edges displays intensely used surfaces, with a deep rounding, transversal striation and a compact wear network. The opposite edges, however, present evidence of the first stages of use, suggesting that after blunting the first edge, the blade fragments were flipped, reattached to the haft and used for a small amount of time before its deposition in the burial. The presence of fresh micro-chips crossing the abraded surface also indicates that the some of the sickle blades were re-sharpened.

Aiterhofen sickle blades showed different degrees of use intensity, from deeply used to only scarcely used. In all cases, there was only one used edge and the localisation of the micro-polish suggest that they were not as diagonally hafted as those from eastern cemeteries (Fig 12D and 12E). At Schwetzingen, a combination of inserts parallel to the handle (Fig 12K and 12L) and diagonally hafted inserts (curved sickle with a serrated cutting-edge) was identified (Fig 12I and 12J). All analysed tools had fresh micro-chips crossing the abraded surface indicating that the active areas were sharpened. The degree of utilisation of the blades is heterogeneous, with some items flipped to use the opposite edge, and some buried with one edge worn. Vendenheim sickle blades were also diagonally hafted and probably inserted in a curved sickle (Fig 12F–12H). Those items displayed only one used edge, indicating that they were not flipped to be re-used.

These data reveal a rather heterogeneous patchwork of harvesting techniques and degrees of artefact reutilisation, probably reflecting regional differences in farming practices.

A significant number of flaked items did not display evidence of use (26%), which suggests either that they were selected or made specially for the purpose of being deposited with the dead or that they were used on materials whose wear is difficult to be created and preserved on flaked tools (such as meat cutting or soft vegetal materials processing) and are therefore more likely to be archaeologically underrepresented. Finally, diverse animal-related activities, including butchering, bone contact and soft animal tissue manipulation appeared very sporadically, with little consistent patterning (Fig 13).

In the case of projectile points, only 11 of the 83 provided clear evidence of possible wear traces (Table 4 in S2 File). Thus, either these tools were made specifically as grave goods, or items in a very good state of preservation were deliberately selected. In LBK contexts, evidence suggests that projectiles were used for both acquisition of animal resources through hunting activities and interpersonal violence. For example, the repeated use of missile weapons (projectile points) against human targets in addition to close-quarter fighting has been demonstrated [97, 107–109], increasing in quantity towards the west. Here, we hypothesis that projectiles were understood by LBK groups as related to these kinds of activity, perhaps as symbolically associating hunting and interpersonal violence as two related activities.

### Hammerstones and handstones

**Fig 14:**
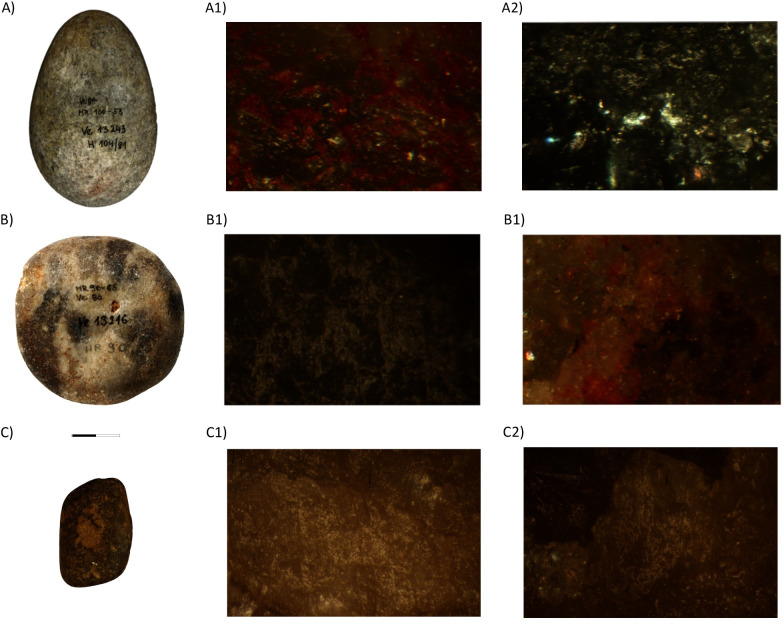
A. Burial 104/81 (Vedrovice). A1) Levelling and ochre residues, 100x; A2) Fresh pecking, 100x; B. Burial 90/80 (Vedrovice). B1) directional micro-polish of semi-closed network and irregular microtopography, 200x; B2) Fresh pecking, 100x. C. Burial 152 Schwetzingen. C1-2) rounding and directional micro-polish of semi-closed network and irregular microtopography, 200x.

Hammerstones and handstones made out of pebbles were also included in this study, although their presence is rather low. Only four tools with sexually determined skeletons provided positive use-wear results: two female burials from Vedrovice (104/81 and 90/80) and two males from Schwetzingen (Table 5 in S2 File) (Table 13). At the first site, they were used to crush a semi-hard material and ochre, and to soften work hide/leather (Fig 14A and 14B). The Schwetzingen pebbles were associated with softening hide/leather and other elastic, abrasive materials (Fig 14C).

**Table 13:** Tools made out of pebbles in sexually determined burials displaying positive use-wear analysis results.

Site	Burial	Code	Sex	Age	RM	Weight (g)	Length (mm)	Thickness (mm)	Width (mm)
Vedrovice	104/81	13243	f	senile	quartzite	185	61	38.2	62
Vedrovice	90/80	13216	f	indet	quartz	204	81	28.9	52.8
Schwetzingen	Stz-132	147.2a	m	mature adult	indet	43	45	13,6	30
Schwetzingen	Stz-152	170.4	m	senile	sandstone	20	41	13	29

## Statistical associations: Activities, sexed skeletons, and PBAs types

PBAs, projectiles and other flaked stone tools were present in less than 20% of burials included in this study: 20% in case of PBAs, 12.5% flaked blades and flakes, 10% projectile points, 3% tools made out of pebbles. Therefore, differences identified between the biological sexes should not be seen as representative of absolute binary gender in the funerary context, but as one indicator of identity in the myriad of gendered identity markers within the symbolic system. Keeping this in mind, our data indicate that lithic tools were much more frequent in males than in female burials (Table 3 in S1 File) (Table 14), a fact that had already been suggested by other authors [34, 49].

**Table 14:** PBAs, projectiles and other flaked stone tools distribution according to age and sex.

	NA among adults	% male graves	% female graves	NA among non-adults	% non-adults
*PBA*	120	76	8	25	17
*Flaked items*	93	67	18	12	11
*Projectiles*	140	77	3	24	14

Abbreviation: NA = Number artefacts.

Thanks to the morpho-technical and functional analysis, the results presented here have generated a more nuanced overview of lithic tools and their relationship to the sex of the deceased (Table 15). PBAs and projectile use-wear variability correlated with sex of the skeleton with whom it was buried (Table 3 in S1 File), taking into account the proportions of females and males at each site (Table 4 in S1 File). PBAs associated with male-sexed skeletons were statistically related to butchering and woodworking activities. Female individuals were only buried with PBAs showing wear arising in soft/elastic animal work, including hide/leather working and soft indeterminable tissues. Projectile points, related to hunting activities and/or interpersonal violence, were almost exclusively buried with male-sexed skeletons (Table 3 in S1 File). The activities identified in the use-wear on flaked tools and on tools made out of pebbles were represented in burials of both sexes without clear evidence of any a sex-determined functional pattern (Table 3 in S1 File). Only in the case of harvesting tools does there seem to be a slight trend towards them being more abundant among males (Table 15).

**Table 15:** Activities identified through use wear analysis according to the sex of the buried individuals.

Activity and tool	Males	Females	Non-adults
PBA—woodwork	21	0	0
*PBA—meat/bone*	13	0	1
*PBA—hide-processing*	1	1	3
*PBA—hard material*	13	0	7
*PBA—soft tissues*	9	2	2
*PBA–“not used”*	5	1	3
*Macro—tools softening + pecking*	2	2	0
*Flaked—harvesting*	7	1	2
*Flaked–hide/leather work*	4	0	1
*Flaked–“not used”*	16	3	1
*Flaked–possible woodwork*	2	1	0
*Flaked–Other animal-related*	5	2	0
*Projectiles*	108	4	24

Non-adults were buried with statistically less lithic tools than males, but with more than females (Table 15) (Table 3 in S1 File) and display evidence of most of the activities represented in both males and females’ burials (Table 15). This may indicate that the same symbolic values applied to children lithic grave goods as well.

Our study confirmed that the morphological characteristics of PBAs were strongly related to their uses and with the sex and age of the individual they were buried with (Table 3 in S1 File) (Table 16). χ2 tests confirmed that females were found exclusively with type 2 tools, which had flat-convex sections, and weighed less, whereas males were accompanied by Ramminger’s tool types 3 and 4 (Table 5 in S1 File). Type 3 was specifically related to butchering and included the heavier tools displaying flat-cylindrical sections and, to a lesser extent, “u”-shaped sections, while type 4 had “u” -shaped sections and intermediate weights. Non-adult individuals were associated with PBAs type 2 and especially type 1 as well as with flat-convex, “u”-shaped and oval sections (Table 5 in S1 File).

**Table 16:** PBA morphological characteristics according to the sex of the buried individuals.

HBI Type/ section	Males	Females	Non-adults
Type 1	13	0	11
Type 2	21	5	9
Type 3	31	0	1
Type 4	22	1	3
Section flat- convex	20	4	9
Section flat- cylindrical	15	0	3
Section oval	3	1	4
Section triangular	6	0	1
Section U	40	2	7

**Fig 15:**
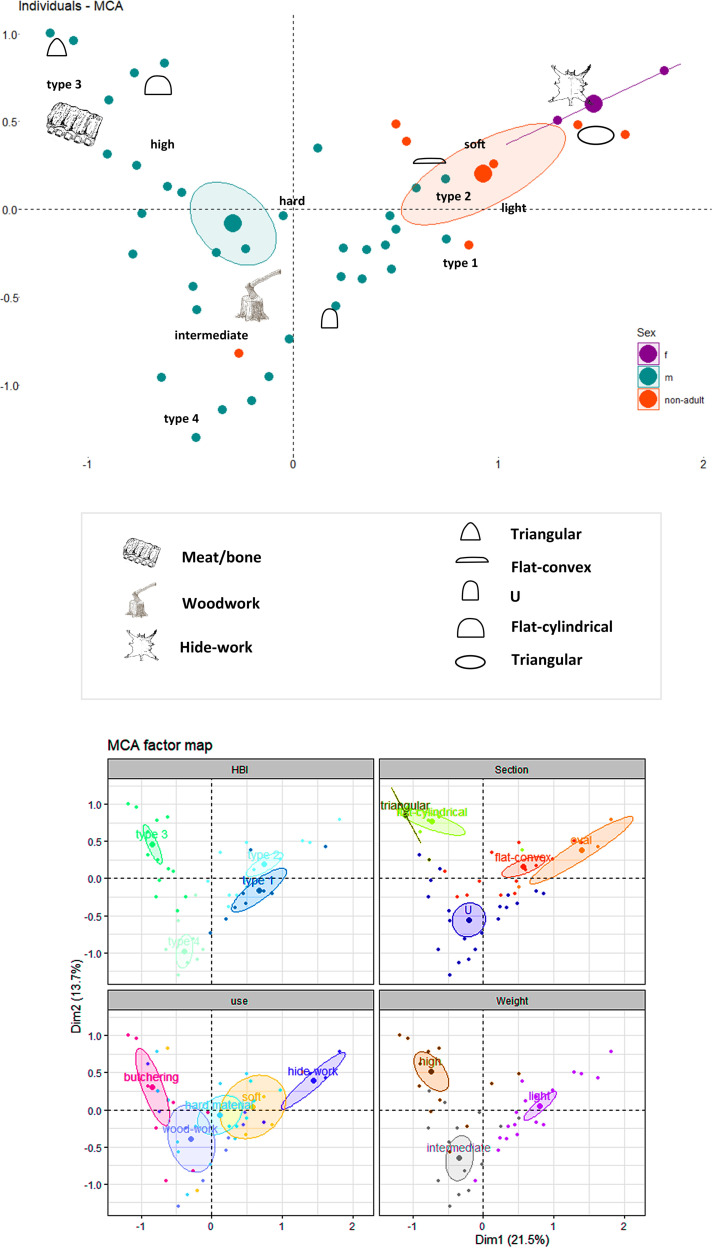
Multiple Correspondence Analysis plot of PBAs morphological characteristics and sex/age of the buried individuals, with items classified according to their weight, HBI index type, use and section (Text 2 in S3 File).

A Multiple Correspondence Analysis (MCA; Text 2 in S3 File) was performed to check the inter-relationships between sex, age, PBA functionality and PBA morphological characteristics (Fig 15). To these categories we added that of weight. Weight groups were characterised by means of a Hierarchical Cluster analysis (Paired group Algorithm, distance 150) as: high (267–393g), intermediate (136–242g) and light (18–104g). The results of the MCA confirmed our hypothesis, allowing a visual overview of the results.

### Regional gender variations

Projectile points and PBAs showed distinct regional patterns with variations falling along an east-west axis following the trajectory of the LBK’s spread (Table 1 in S1 File), and consistent with presence of variability in other cultural and gender patterns.

The frequency of projectiles in graves increases from east to west (between 0 to 13% among eastern cemeteries, climbing to c.17% at Vendenheim), as well as the number of items displaying evidence of wear (Table 4 in S2 File). These results are even more significant if the male/female ratios per site are considered (Table 4 in S1 File), as females were more frequently buried than men both at Schwetzingen and at Vedrovice.

Of the total analysed and functionally determinable projectiles, 11 cases provided evidence of possible use-wear, all of them belonging to the Aiterhofen and Schwetzingen assemblages. These results indicate that, at least at the eastern sites, projectiles may have been made specifically with the intention of being deposited as grave goods or there was a specific selection of already used items in a very good state of preservation [43]. This pattern may have changed further west, where they not only appear more often, but they also appear in a more used state in funerary contexts. In addition, projectile technical characteristics change, from trapezoidal tips with low elongation indexes in the east, to triangles with high elongation indexes in western sites and Aiterhofen (*vide supra*).

Thus, we can argue that arrowheads may have replaced PBAs as items symbolising interpersonal violence, as farming spread westwards. At the western-most extent of the LBK, in the Paris Basin PBAs completely disappear from grave goods assemblages and could symbol the final progression of this trend. However, this phenomenon could also be related to a change in the economic and symbolic importance of hunting. To date, only in the Aisne valley (Paris Basin) have animal bone assemblages indicated a differentiation between hunted and domestic animals by occurring in different frequencies alongside houses [110]. Here, houses of “hunter” and “farmers” were thought to live alongside each other at the same settlement, perhaps representing that the action of hunting played a role in a more circumspect range of identities.

PBAs types and uses among male graves do vary significantly across Europe (*vide supra*): at eastern sites, the presence of use-wear suggesting animal treatment (butchering, soft animal tissues) accounted for 76.4% of identifiable use-wear, mostly found on type 3 PBAs. At the western sites and Aiterhofen, woodworking increases, accounting for 50% of the use-wear. In the west, “u”-shaped and flat-convex sections are the most abundant with types 1 and 4 (Vendenheim) and 1, 2 and 4 (Aiterhofen). These results suggest a possible east-west change, in the way hunting, fighting and woodworking were socially valued and hence symbolised at death.

## Multi-proxy comparison: Lifeways and burial data

### Isotopic and grave good data presentation

We now turn to examine whether the sex and geographic variations in the presence and use-wear of stone tools co-varied with other grave goods, as well as with the available isotopic data which informs on lifeways.

Three isotopes formed the focus on the analysis, δ^15^N and δ^13^C, which charts diversity in protein consumption, and ^87^Sr/^86^Sr, which can indicate lifetime residential mobility. Including the study of diet in the sexual division of labour is relevant here, as food was largely the product of many of the tasks thus represented by the use-wear analysis.

All the sexed adult inhumations with isotopic values were included in the analysis. The number of sampled skeletons is statistically representative of the studied population, except in the case of Vendenheim (Table 17).

**Table 17:** Quantification of buried individuals sampled for 87Sr/86Sr and δ15N/ δ13 isotopes, and percentage of each sex group of sampled individuals according to the number of males (m)/females (f) per site.

**DIET Individuals sampled for δ**^**15**^**N/ δ**^**13**^							
**SEX/AGE**	**f**	**indet**	**m**	**non-adult**	**Total**	**% f sampled**	**% m sampled**
**Aiterhofen**	22	3	24	7	56	51	44
**Kleinhadersdorf**	9	3	13	8	33	82	93
**Nitra**	24	0	14	2	40	92	74
**Schwetzingen**	53	0	47	3	103	84	90
**Vedrovice**	40	1	21	2	64	98	100
**Vendenheim**	2	9	6	12	29	x	x
**Individuals sampled for** ^**87**^**Sr/**^**86**^**Sr**							
**SEX/AGE**	**f**	**indet**	**m**	**non-adult**	**Total**	**% f sampled**	**% m sampled**
**Aiterhofen**	21	0	33	7	61	49	61
**Kleinhadersdorf**	10	2	11	6	29	91	79
**Nitra**	17	3	13	12	45	65	68
**Schwetzingen**	52	0	46		98	83	88
**Vedrovice**	28	0	12	12	52	68	57
**Vendenheim**	2	14	9	12	37	x	x

As the dietary isotopic data confirms a terrestrial diet, based on C3 plants, any δ^15^N variations are likely to indicate differential protein ingestion [62, 68, 69, 111] (Table 1 in S2 File). δ^15^N values were not statistically different between male and female individuals at the sites, excepting Vedrovice [43, 70].

**Fig 16:**
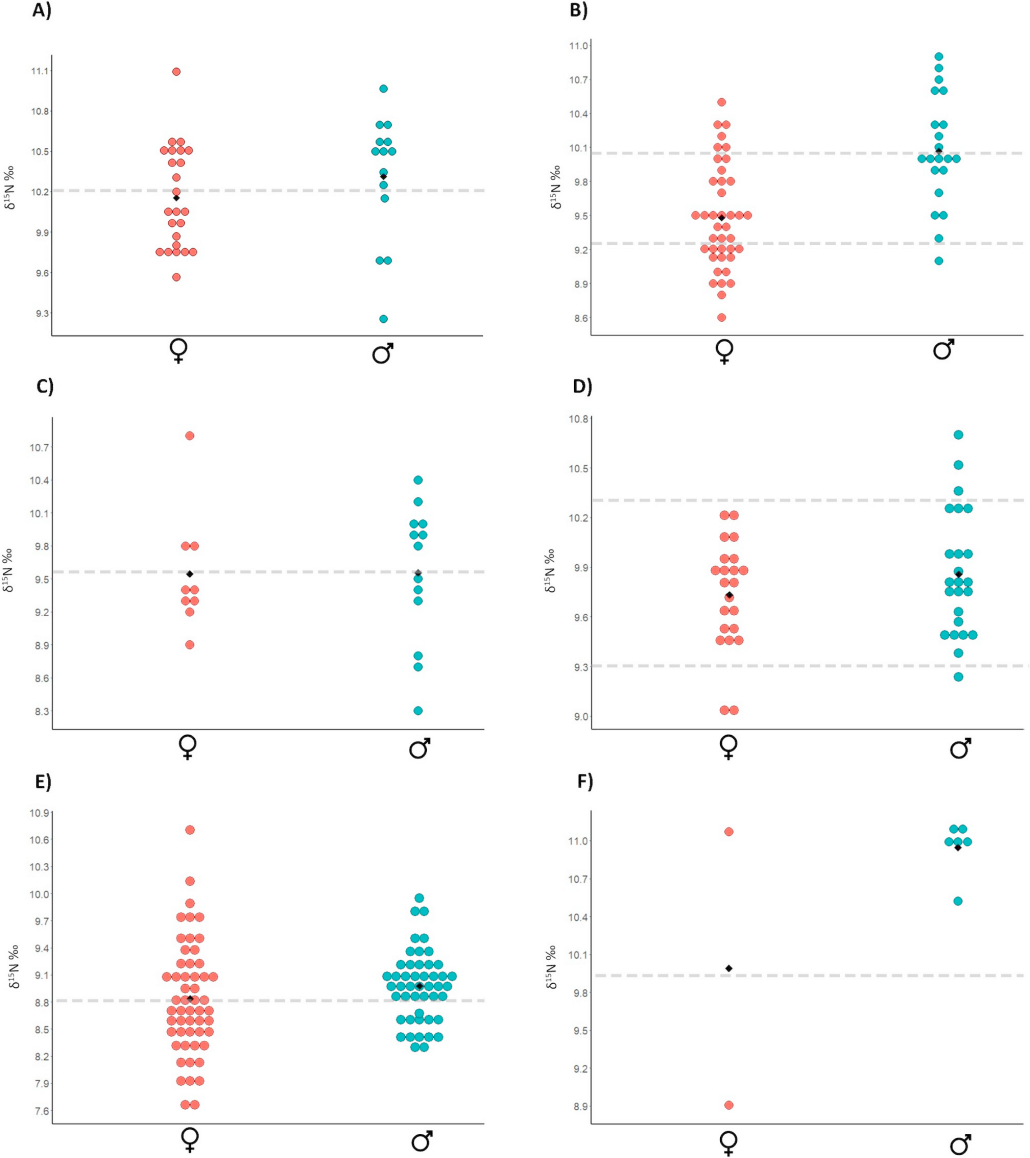
Dot plot including δ15N values according to sex. Grey discontinuous line = separation between NCL1 (down), NCL2 (up) made by us. Black diamond: mean. A) Nitra; B) Vedrovice; C) Kleinhadersdorf; D) Aiterhofen; E) Schwetzingen; F) Vendenheim. Red dots = female; blue dots = male.

However, Hierarchical Cluster Analysis (Ward’s method–distances 1–2.5) identified clusters of burials with similar δ^15^N values in each cemetery. Kruskal-Wallis Tests confirmed that the clusters were statistically significant (Table 6 in S1 File). As a result, at least two groups were recognised in each cemetery and classed respectively as possessing “high” and “low” δ^15^N values. It must be noted that for these groups there is only a small difference between the δ^15^N values in real terms, meaning this pattern should not be taken as representing two significantly different diets. The observation of the data under this parameter indicates that while both males and females share a range of Nitrogen values, more of those with lower δ^15^N values were biologically female and more of those with higher δ^15^N values were biologically male (Fig 16). This has been here interpreted as evidence that, although some female individuals shared the same protein ingestion rates with males, there were more women consuming lower amounts of protein than men. In some cases (such as Schwetzingen, Vedrovice and Nitra) this translated into females presenting a more variable dietary pattern than males.

These δ^15^N differences could also be the result of males and females consuming animals that grazed in areas with different manuring regime intensity or animals with access to food sources enriched with δ^15^N, such as meat and human waste. Archaeobotanical and stable isotopic analysis suggest that manuring systems and livestock diets changed across the LBK regions [112]. More studies in this direction are needed to understand animal-crop husbandry systems within communities and help elucidating the impact of those practices in male and females’ lifeways.

Strontium isotopes ^87^Sr and ^86^Sr recorded in human bone and teeth are a widespread method used to determine geographic origin and mobility patterns [113, 114]. Sr isotopic signatures make their way from local geology into the mineral composition of the human skeleton through the diet and local water sources. Commonly, an individual is considered ‘local’ if his or her strontium isotope ratio falls within the ‘local’ baseline range, or ‘non-local’ if the value falls outside [115]. We are aware that the dichotomy between ‘locals’ and ‘non-locals’, however, can lead to ambiguities, since such categories depend on their historical context [for further discussion about the problems related to what can be considered “local” or “non-local” in prehistory see 116]. A further challenge for interpretation arises in small areas with a high strontium variability. In these cases, individuals appearing as “non-local”, might only have travelled a small distance.

**Fig 17:**
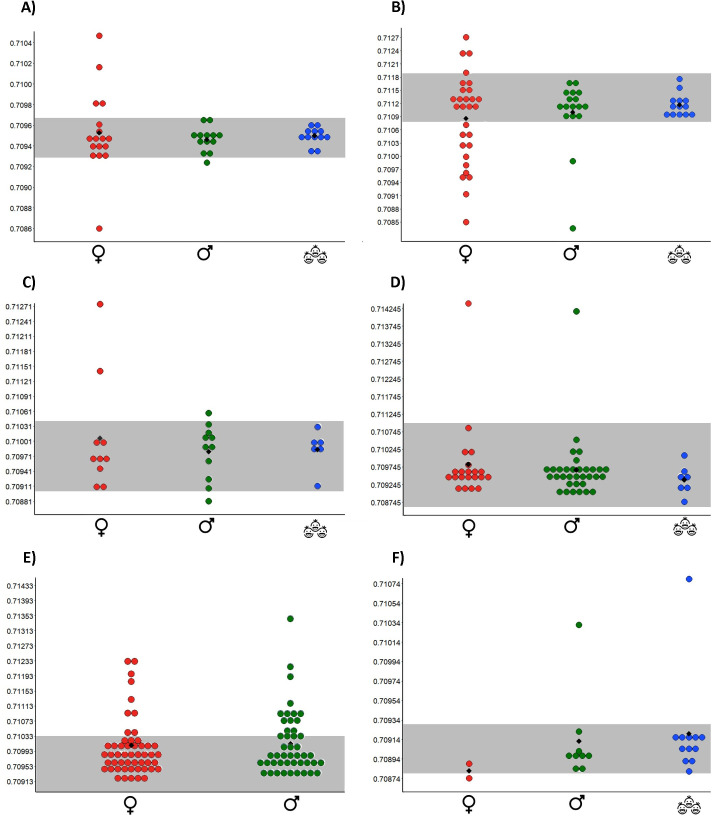
Dot plot including 87Sr/86Sr values and 87Sr/86Sr concentration according to the sex/age of the buried individuals. A) Nitra; B) Vedrovice; C) Kleinhadersdorf; D) Aiterhofen; E) Schwetzingen; F) Vendenheim. Grey shadow = “local” range. Red dots = female; green dots = males; blue dots = nonadults. Black diamond: mean.

As strontium isotope data from local animals are not available, nor are baseline models predicting geological strontium variations (except in the case of Schwetzingen and indirectly Vendenheim and Aiterhofen), a range of values for children and juveniles was used as the “local” indicator in eastern sites (Fig 17A–17C). This approach assumes that children were more likely to be local because they had less time to migrate than adults in their lifetime [117].

In the case of Aiterhofen, the majority of the sampled individuals have ratios which match the local geology of the area [118] with only two outliers whose origin can probably be traced to the Bavarian Forest [Fig 17D, 119]. Bickle et al. [119] plotted the ^87^Sr/^86^Sr ratios against 1/Sr ppm (parts per million), which revealed two possible mixing lines for men based on their diet (more or less Sr-rich foods), which may have corresponded to transhumance or hunting being performed on different soils (e.g. transhumance). At Schwetzingen the local signature of the area is between 0.7085 and 0.7103, with the loess thought to have a ^87^Sr/ ^86^Sr ratio at or below about 0.710 [70, 120, 121] (Fig 17E). At Vendenheim the “local” signature of the area is between 0.7085 and 0.71 [122]. In this case the majority of the sample fits this range with three outliers (Fig 17F).

The ^87^Sr/^86^Sr ratios have already confirmed that females were more likely to be classed as “non-local” than males in eastern sites, particularly at Vedrovice and Nitra [28, 43, 50, 62] (Table 18). The situation changes at Aiterhofen (Bavaria), where differences between the sexes have been argued to be based around some women moving between communities and regular mobility (e.g. transhumance) among men [119]. At Schwetzingen (Baden-Württemberg), the isotopic data points towards a major mobility among males (Table 18). In this last case, this pattern has been interpreted as the result of use of nearby uplands for animal husbandry [30, 123]. These observations can be interpreted in terms of a possible change in mobility patterns, away from the otherwise largely favoured interpretation that virilocal marriage was dominant for the LBK.

**Table 18:** Female and male mobility data according to the site.

	F non-local	M non-local	F local	M local
**Nitra**	5	0	12	13
**Vedrovice**	14	2	15	15
**Kleinhadersdorf**	2	0	8	11
**Aiterhofen**	1	1	20	32
**Schwetzingen**	10	15	42	31

Abbreviations: M = male; F = female.

All the grave goods recurrently deposited within burials were included in the analysis: projectiles, PBAs, flaked items, bone tools, entire pottery vessels, *Spondylus* ornaments and unfurnished graves (Table 1 in S2 File). Other items were not included due to being too infrequently found in graves to assess their distribution statistically. The excluded grave goods included: firelighter kits (flint and pyrite), other kind of ornaments (i.e. different kind of freshwater shells, human and animal teeth, stone and marble beads, board tusks, manganese and graphite beads), grinding stones, perforated iron oxides, flint knapping stones and animal parts (fox, dog, cow, sheep, goat, pig and deer).

The presence of pottery vessels, *Spondylus* items and bone tools statistically varied between males and females (Table 7 in S1 File). However, the proportion of the pottery vessels and *Spondylus* ornaments distribution among sexes was not consistent between the cemeteries reflecting an intense regional heterogeneity (Table 19, for a more detailed observation of the data see Table 8 in S1 File and Figs 1–3 in S1 File). Only in the case of bone tools, could a similar pattern to stone tools be ascertained, as they were much more abundant in male graves.

**Table 19:** Count of the percentage of presence/absence of major categories of grave goods according to the amount of female and male buried skeletons in each cemetery.

	Pottery F	Pottery M	Spondylus F	Spondylus M	Bone F	Bone M
**Nitra**	54	22	7	11	2	6
**Vedrovice**	32	71	136	43	0	43
**Kleinhadersdorf**	18	16	19	11	4	16
**Aiterhofen**	21	15	8	12	6	25
**Schwetzingen**	24	38	7	5	7	43

Schwetzingen’s bone arrowpoints were not included in this count. Abbreviations: F = female; M = male.

This is an interesting point, as it reinforces the different mortuary treatment that tools receive from ornaments, possibly reflecting different symbolic systems of value. In the case of pottery vessels, more research is needed in order to properly address their meaning. While typologically well described the use of pottery (e.g. determined by lipid analysis) in funerary contexts has not yet been considered.

### Multiple Correspondence Analysis

**Fig 18:**
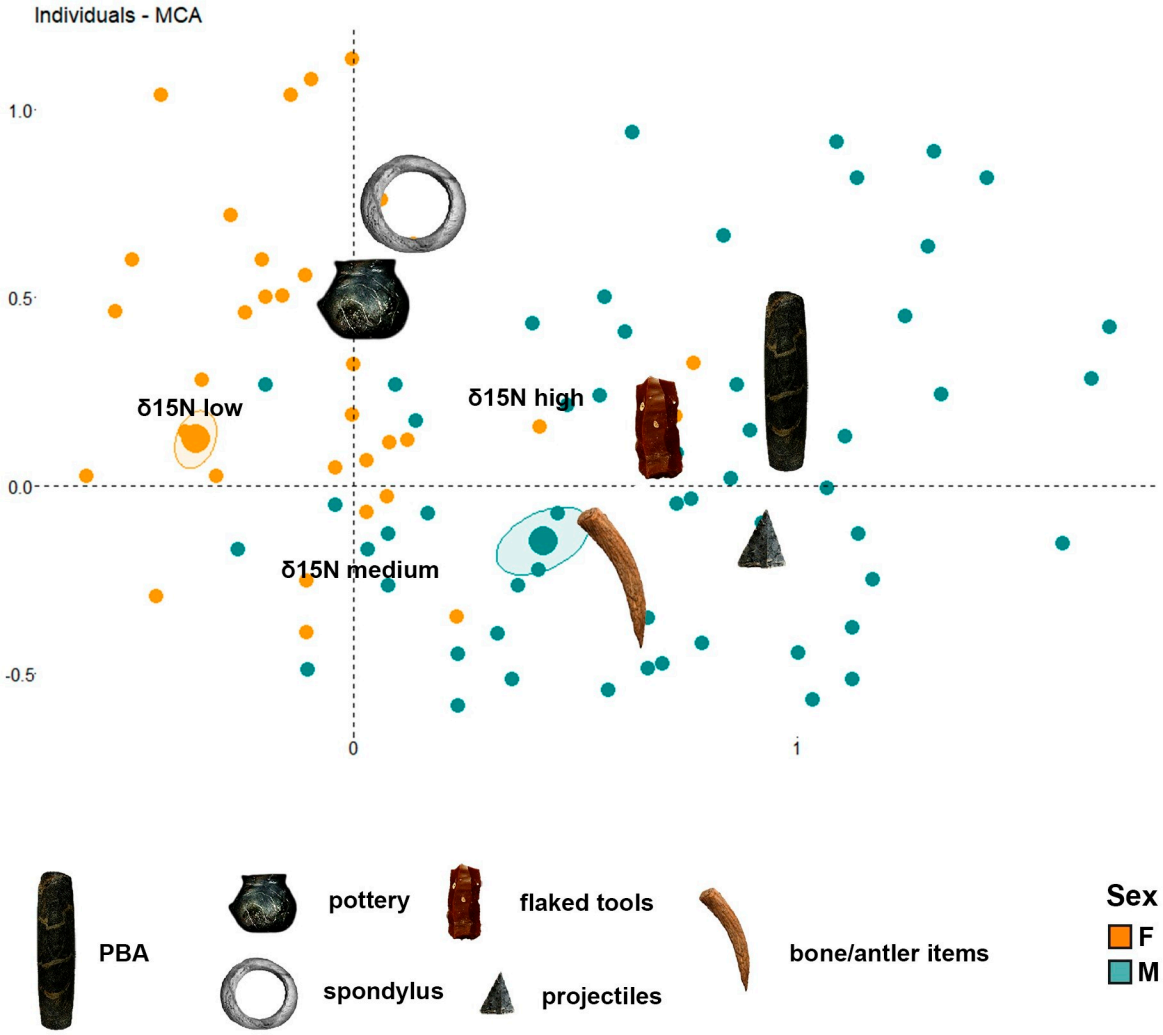
Multiple Correspondence Analysis plot of grave goods presence, sex (male/female) and isotope indicators of diet and mobility including only furnished graves.

Correlation between biological sex and mobility, diet groups, and grave good distribution and function, was explored through MCA (Multiple Correspondence Analysis). Bearing in mind that the female dichotomy between local/non-local origin is only strong at the eastern sites we decided to run the test twice: the first time excluding the mobility data (Text 3.1 in S3 File, comprising 276 burials in total) (Fig 18), the second including it and separating the database between the eastern sites (Text 3.2 in S3 File) and Schwetzingen (Text 3.3 in S3 File).

The results indicate that not only did males present less unfurnished graves (27%) than females (48% of the total), but that the grave good assemblages were also different. The males whose graves were furnished (73% of the total) were associated with stone tools and were related to hunting activities/interpersonal violence (PBAs/ projectiles), butchering (PBAs), woodwork (PBAs), bone instruments and only very infrequently to harvesting (sickle blades).

Furthermore, high δ^15^N dietary values tended to be related to male individuals (Fig 18). Males with high δ^15^N dietary values were more likely to be buried with more stone and bone tools as well as with more ornaments and pottery vessels, whereas those males with medium δ^15^N dietary values correlated with a lower presence of grave goods.

In contrast, the females were related to lower δ^15^N clusters, even if some of them fell within the medium nitrogen cluster (Fig 18). The female-sexed furnished graves (52% of the total) do not present statistical associations to any kind of grave good in particular, even if they showed at closer association with pottery vessels and *Spondylus* ornaments than males. In the rare cases where a woman was buried with a PBA, the use-wear suggests these tools were used for soft/elastic animal work, including hide/leather working and soft indeterminable tissues. Significantly, these women were mainly restricted to senile/mature adult ages (Table 1 in S2 File), suggesting age formed a significant identity trait for females.

Among the eastern sites where virilocality has been argued to be practiced (*vide supra*) there was a correlation between the individual’s mobility and their δ^15^N values, the grave good distribution, and sex (Text 3.2 in S3 File). Local skeletons had higher δ^15^N values than non-locals, which tended to present with medium values. Having a non-local isotopic signature is also more closely associated with females than with males, as well as being less probably related with stone and bone tools. The pottery vessels and *Spondylus* ornament frequencies do not apply to that rule, as they are the grave goods most likely to accompany the non-local females.

At Aiterhofen almost all the population is local, so no further patterning could be determined. At Schwetzingen, where the amount of non-local males is higher than females, sex determines the probability of having or not having stone and bone tools as well as displaying higher or lower δ^15^N values (Text 3.3 in S3 File). However, the mobility of individuals no longer correlates with any of these categories, matching the pattern found in the east. In this case, non-locals were associated with higher δ^15^N values, and locals to medium δ^15^N values and pottery vessels. As the mobility patterns in this area have been interpreted as the result of different farming practices which included use of unsettled uplands for animal husbandry (*vide supra*), we suggest a possible division of labour involving pastoralist practices, that may also be related to differences in diet between the sexes.

## Discussion and conclusions

The use-wear and technological analysis of LBK stone tool grave goods suggests the presence of a sexual division of labour associated with PBAs, bone tools and projectiles. This evidence, however, should not be considered as representative of absolute binary categorisation based on sex, as stone tools were present in fewer than half of the grave good assemblages.

Grave goods may well not be representative of everyday life but provide a particular formalised setting in which symbolic versions of sex were presented. The male biological sex is associated with tools used in butchery, woodwork, hunting, and possibly interpersonal violence. It is striking that, violence-related trauma on human skeletons is most frequently found on adult females and juveniles in LBK [29], whereas our data suggests adult males were the ones associated with these weapons. In contrast to males, females are not often associated with bone and stone tools, and, in those rare cases where it they are, PBAs and flaked tools were used for soft/elastic animal work, such as processing hides and skins. In this sense, there are no relevant discordances between sex estimated through osteological study and gendered toolkits in grave goods.

Not only use-wear provided sexed-based evidence. Females’ PBAs displayed restricted and distinct typological features (type 2 shapes, flat-convex sections, and light weights) different from males’ (types 3 and 4, U and flat-cylindrical sections and high/intermediate weights) and sharing some characteristics with non-adults’. This pattern could be explained either by the fact that each type’s morphological characteristics were the most suited for the intended function (different between males and females) or by social norm that required sex-differentiated artefact shape variation. This gendered PBAs morphological characteristic categorisation had been suggested by Ramminger [124] in the German area of Hesse and by Van de Velde [125] in the cemetery of Elsloo (Netherlands), and is now confirmed for a wider area.

Although PBA types 3 and 4 (associated with males) are more frequent than type 2 (associated with females) in the funerary sphere, the reverse is true at LBK domestic contexts as our own observations at LBK sites of Altscherbitz (Saxony) and Bischoffsheim (Alsace), and Ramminger’s [124] in Hesse attest. This implies different symbolic associations for each tool type, as we would expect alongside the hide working represented by type 2 PBAs, woodworking and animal butchery to also take place at settlements. Male labour, associated with hunting, animal butchery or even interpersonal violence could thus have been a strong symbolic focus for funerary rites.

This evidence is in line with the presence of sexually differentiated activity patterns in upper and lower limbs identified through osteology (muscular-skeletal stress markers, paleopathologies, and labour-related sexual asymmetry) that have been related to a possible LBK sexual division of labour (2015, 23, 31, 32, 33). Research of humeral morphology in Central-European LBK populations found asymmetry based on sex, with male humeri more asymmetric than females’, which was interpreted as suggesting more unilateral load [32]. Cross-sectional geometric analysis of lower limb bones in Central European communities confirmed sexual dimorphism with males having significantly higher femoral shape ratios and tibial TA (total subperiosteal area, estimating compressional strength) and J (polar second moment of area, indicating bending and torsional rigidity) than females [31, 33]. This evidence suggests either differentiated mobility patterns and/or distinct activities involving lower limb biomechanics, indicating at least a partial sexual division of labour.

Other indicators of labour such as grooves on upper incisors probably remnants of some type of dental manipulation such as passing of flexible material over anterior the teeth in a repetitive and habitual fashion in processing materials as sinews for bow strings or plant fibres for basketry or weaving, were more frequently found in females than in males, at least at the eastern sites [29, 50, 126, 127].

Detailed analysis at Nitra revealed that the lower limbs (femur and tibiae) measurements support a clear sexual dimorphism in the bone deformation, as male tibiae and femurs tend to be bigger and generally more robust [50]. Furthermore, they indicate a tendency of females to present more grouped and homogeneous values than males, possibly reflecting an inferior variability of movements and tasks. Pronounced musculoskeletal stress markers were recorded confirming the heavier physical load of males and their unilateral tasks, while upper limb data shows opposite trends between males and females suggesting very different nature of movements and workloads according to sex. Unfortunately, detailed studies on occupational stress in bones or sign of violence on the skeletons are limited to a few sites and do not often focus on identifying sexed-based differences. A higher bone fracture rate in LBK males was identified by Hedges et al. [29] among the *Lifeways* project sample, which may be seen as indicative of sexual division of labour, where males, through the activities they carried out, were at a higher risk of sustaining such trauma. More in-depth analysis is needed in this direction could yet add further nuance to the range of possible sex-differentiated activities.

Osteological evidence suggests that the spectrum of activities differentiated by sex is larger than that found within the grave good use-wear, suggesting a symbolic selection of certain tasks were chosen as funerary identity-markers. Other activities were not chosen to be represented among grave goods. For instance, there is an evident lack of macrolithic tools used in plant food processing in burials, which are well represented in domestic contexts, and cereal processing is likely to have been a task performed on a daily basis.

What this analysis cannot assess are grave goods made of organic materials, such as textiles, hides, wood artefacts, ornaments or other kinds of items made with vegetal fibres that had not been preserved. In some cases, such as the cemetery of Kleinhadersdorf [128] organic objects have been proposed to be present in currently “empty” areas in the graves. These items could have possibly been part of the gender identity codification, particularly as the use-wear analysis demonstrates extensive working with soft materials.

On the other hand, it seems like there is a significant symbolic differentiation between stone and bone tools, and ornaments and pottery, as they correlate differently in the assemblages. In the case of pottery vessels, future research may shed light into their use (e.g. lipid or protein analysis). Ornamentation is another form of grave good that differ in their frequency, regional diversity and place in the grave, from tools, representing a further dimension of funerary practice that may cross-cut the symbolism of certain tasks identified in this study. Thus, we see further division within each of the sexes as cautioning against seeing early farmers as living within a strict binary sexual hierarchy.

Correlations between biological sex, grave goods and isotopic values indicating diversity in diet and mobility, strengthen the link between the data found in the burial context and conditions in life. It is certainly striking the fact that different dietary groups in life received different treatment after death, and that those dietary groups were tightly related to biological sex. In this sense, higher δ^15^N dietary values reflecting a richer protein intake tended to be related to male individuals. In turn, the higher those δ^15^N values were, more were males likely to be buried with more stone and bone tools as well as with more ornaments and pottery vessels. In contrast, the females were related to lower-medium δ^15^N clusters and did not present statistical associations to any kind of grave good in particular, even if they were closer to pottery vessels and *Spondylus* ornaments than males.

Our results stress the importance of addressing gender and sex studies on the basis of the particular contextual characteristics of every community, as has been previously stated [24, 42]. Even in the 150-year period represented by this study, gender patterns proved to be dynamic as farming spread westwards, consistent with the presence of variability in cultural patterns. Those changes in social and symbolic means of expression include shifts in the human mobility patterns, possibly including exogamy practices, an increased presence of unfurnished inhumations, changes in the frequencies and characteristics of certain items considered as status and gender identity markers (*Spondylus* ornaments, polished adzes and arrowheads) [34, 42, 43, 62, 129].

This east-west cline in the stone tools grave good assemblages and distribution may be related to variations in hunting techniques or even warfare styles. This pattern may represent an increased symbolic importance of hunting wild animals as the LBK moved westward. Thus, we suggest that the symbolic associations of projectiles became more potent than the activities performed with PBA, and hence held increased value in the funerary sphere. Changes in the economic and symbolic importance of hunting has been observed in Aisne valley longhouses (Paris Basin), where a distinction between domesticated animals and hunting was identified [110]. These practices can thus be seen as a set of changes in cultural traditions and symbolic practices that increased as Neolithic reached the Paris Basin [34, 49, 130], and are still the object of debate. Chronology remains problematic in this regard, as early LBK graves dominate in the eastern regions, whereas burials increased in number in the later phases of the western distribution.

Mobility patterns also differed from east to west. Among the eastern sites where virilocality has been argued for (Nitra, Vedrovice and Kleinhadersdorf), local skeletons included almost all male and some of the female individuals. Locals were related to higher δ^15^N values than non-locals, as well as having a higher probability of being buried with stone and bone tools. Here, pottery vessels and *Spondylus* ornaments do not follow the same rules, as they are the grave goods most likely to be accompanying the non-local females, suggesting, again, a different symbolism for these kinds of grave good. In contrast, at Schwetzingen, non-locals tended to be males and were associated with higher δ^15^N values, and locals to medium δ^15^N values and pottery vessels. In this case the “non-local” mobility patterns have previously been related to animal husbandry movements and not to exogamy practices, which makes it reasonable to interpret these results as representing a possible division of labour involving pastoralist practices, which may have in turn influenced dietary choices and access.

In conclusion, our results make a contribution towards gaining a more rounded understanding of the sexual division of labour during the Neolithisation of Europe. Task specialisation is considered to have had a foundational role in the emergence of property, surplus accumulation, political power concentration and social exploitation [131–135]. However, these processes are often interpreted without considering possible sexual divisions in labour and gender symbolic systems, often allowing a binary gender hierarchy dominated by males to be an implicit factor in Neolithic social systems.

This paper reveals evidence that could sustain the hypothesis that the roots of gender inequalities are found, in part, in the roles carried out by females during the demographic and technological changes of Neolithisation. Indeed, the sexual division of labour has been hypothesised in the Near East [18, 20, 136, 137], where females have been associated with grinding and fibre processing and men with hunting. Our results indicate that, to a certain extent, sexual division of labour may have been part of the colonisation of the European continent by farmers, represented in at least two, if not more, separate symbolic spheres for the sexes in the funerary context.

In sum, our data cautions against simple models of either binary gender hierarchy or complete equality, but rather a complex and dynamic pathway rooted in a sexed division of labour from the earliest Neolithic. We have demonstrated that accounts of the transition to the Neolithic cannot ignore the sexual division of labour if they wish to further understanding of the development of task specialisation, production and the development of inequalities–the social consequences of this turning point in human prehistory.

## Supporting information

S1 File Statistics. (DOCX)

S2 File Database. (XLSX)

S3 File MCA analysis. (DOCX)
