# Supplementary material for: A sexual division of labour at the start of agriculture? A multi-proxy comparison through grave good stone tool technological and use-wear analysis
Source: PLoS One. 2021 Apr 14;16(4):e0249130. doi: 10.1371/journal.pone.0249130 (PMC8046253; doi:10.1371/journal.pone.0249130)

**Supporting Information 1**

Table 1: Resulting p-value χ2 and Kruskal-Wallis test of correlating stone tools morpho-technical characteristics and the site representation.

| **Stone tools morpho-technical characteristics** | **Kruskal-Wallis test** | **χ2** | **df** | **P** |
| --- | --- | --- | --- | --- |
| HBI type/ site |  | 155.63 | 18 | 0.000 |
| PBA weight/ site |  | 59.951 | 10 | 0.000 |
| PBA section/ site |  | 69.827 | 25 | 0.000 |
| Flaked tools length measurements/site | Kruskal-Wallis test | 21.18 |  | 0.000 |
| Flaked tools width measurements/site | Kruskal-Wallis test | 23,35 |  | 0.000 |
| Projectile elongation index/ site | Kruskal-Wallis test | 87,31 |  | 0.000 |
| Flaked presence (negative result)/ site |  | 7.378 | 5 | 0.194 |
| Projectile presence/ site | Kruskal-Wallis test | 16.698 |  | 0.000 |

Table 2: Resulting p-value χ2 of correlating PBAs techno-cultural evidence and uses.

| **PBAs techno-cultural evidence** | **χ2** | **df** | **P** |
| --- | --- | --- | --- |
| HBI type/ weight | 40.716 | 6 | 0.000 |
| HBI type/ section | 56.123 | 12 | 0.000 |
| Weight/section | 24.294 | 8 | 0.002 |
| Use/ weight | 15.741 | 8 | 0.046 |
| Use/ HBI type | 16.805 | 9 | 0.052 |
| Use/ section (negative result) | 8.448 | 9 | 0.490 |
| Use/ site | 8.905 | 4 | 0.063 |

Table 3: Resulting p-value χ2 of correlating the PBAs, projectiles, and other flaked tools with the sex (female/male) and age (adult/non-adult) of the buried individuals.

| **Variable** | **χ2** | **df** | **P** |
| --- | --- | --- | --- |
| Projectile presence and sex | 39.049 | 1 | 0.000 |
| PBA presence and sex | 78.441 | 1 | 0.000 |
| Flaked tools presence and sex | 17.377 | 1 | 0.000 |
|  |  |  |  |
| Projectile presence and age | 57.117 | 2 | 0.000 |
| PBA presence and age | 47.120 | 2 | 0.000 |
| Flaked tools presence and age | 28.735 | 2 | 0.000 |
|  |  |  |  |
| PBA use and sex | 61.647 | 7 | 0.000 |
| Flaked tools use and sex (negative result) | 2.17 | 4 | 0.703 |

Table 4. Quantification and proportions of female, male and sexually indeterminate individuals according to the sites.

| Site | f | f % | ind | m | m % | Total adults |
| --- | --- | --- | --- | --- | --- | --- |
| Aiterhofen | 43 | 36 | 21 | 54 | 46 | 118 |
| Kleinhadersdorf | 11 | 37 | 5 | 14 | 47 | 30 |
| Nitra | 26 | 50 | 7 | 19 | 37 | 52 |
| Schwetzingen | 63 | 50 | 12 | 52 | 41 | 127 |
| Vedrovice | 41 | 63 | 3 | 21 | 32 | 65 |
| Vendenheim | 3 | 4 | 59 | 13 | 17 | 75 |

Table 5. Resulting p-value χ2 of correlating PBAs techno-cultural evidence with the sex and age of the buried individuals.

| **PBAs techno-cultural evidence** | **χ2** | **df** | **P** |
| --- | --- | --- | --- |
| Sex/ section (negative result) | 8.544 | 4 | 0.074 |
| Sex/ HBI type | 10.099 | 3 | 0.018 |
| Sex/ weight (negative result) | 5.256 | 2 | 0.072 |
| Age-sex/ HBI type | 24.997 | 6 | 0.000 |
| Age-sex / section (negative result) | 14.680 | 10 | 0.144 |
| Age-sex / weight | 13.960 | 4 | 0.007 |

Table 6: Tests involving the Nδ15 clustering.

| **Female/male Nδ15 Ward’s Method Algorithm distance** | **Differences between clusters** |
| --- | --- |
| Vedrovice, distance 1 | Kruskal-Wallis Test, 8.109E-13 |
| Nitra, distance 1,5 | Mann-Whitney Test, 2.3683E-07 |
| Kleinhadersdorf, distance 1.5 | Kruskal-Wallis Test, 1.554E-05 |
| Aiterhofen, Paired group, 0.6 | Kruskal-Wallis Test, 4.407-06 |
| Schwetzingen, distance 2,5 | Mann-Whitney Test, 1.667E19 |
| Vendenheim - The little preservation of sexed skeletons prevented identifying differences between the sexes. | |

Table 7: Resulting p-value χ2 of correlating the percentages of pottery vessels, *Spondylus* items and bone tools presence among sexes based on percentages according to the number of male/female burials in each cemetery.

|  | **χ2** | **df** | **P** |
| --- | --- | --- | --- |
| Pottery vessels/ sex | 32.032 | 4 | 0.000 |
| *Spondylus*/ sex | 20.369 | 4 | 0.000 |
| Bone tools/ sex | 9.7489 | 4 | 0.0448 |

Table 8. Count of presence/absence of major categories of grave goods according to the sex of the buried skeletons. Schwetzingen’s bone arrowpoints were not included in this count. Abbreviations: P= presence; A= absence; F= female; M= male.

|  | Nitra | Vedrovice | Kleinhadersdorf | Aiterhofen | Schwetzingen | Vendenheim |
| --- | --- | --- | --- | --- | --- | --- |
| Pottery F P | 14 | 13 | 2 | 9 | 15 | 0 |
| Pottery F A | 12 | 28 | 9 | 34 | 48 | 3 |
| Pottery M P | 12 | 10 | 3 | 8 | 8 | 1 |
| Pottery M A | 7 | 11 | 11 | 46 | 44 | 12 |
| Spondylus F P | 3 | 15 | 5 | 5 | 3 | 0 |
| Spondylus F A | 23 | 26 | 6 | 38 | 60 | 3 |
| Spondylus M P | 6 | 6 | 2 | 6 | 1 | 0 |
| Spondylus M A | 13 | 15 | 12 | 48 | 51 | 13 |
| Bone F P | 1 | 0 | 1 | 4 | 3 | 0 |
| Bone F A | 25 | 41 | 10 | 39 | 60 | 3 |
| Bone M P | 3 | 6 | 6 | 13 | 9 | 5 |
| Bone M A | 16 | 15 | 8 | 41 | 43 | 8 |

Figure 1: *Spondylus* distribution among sexes based in each cemetery.


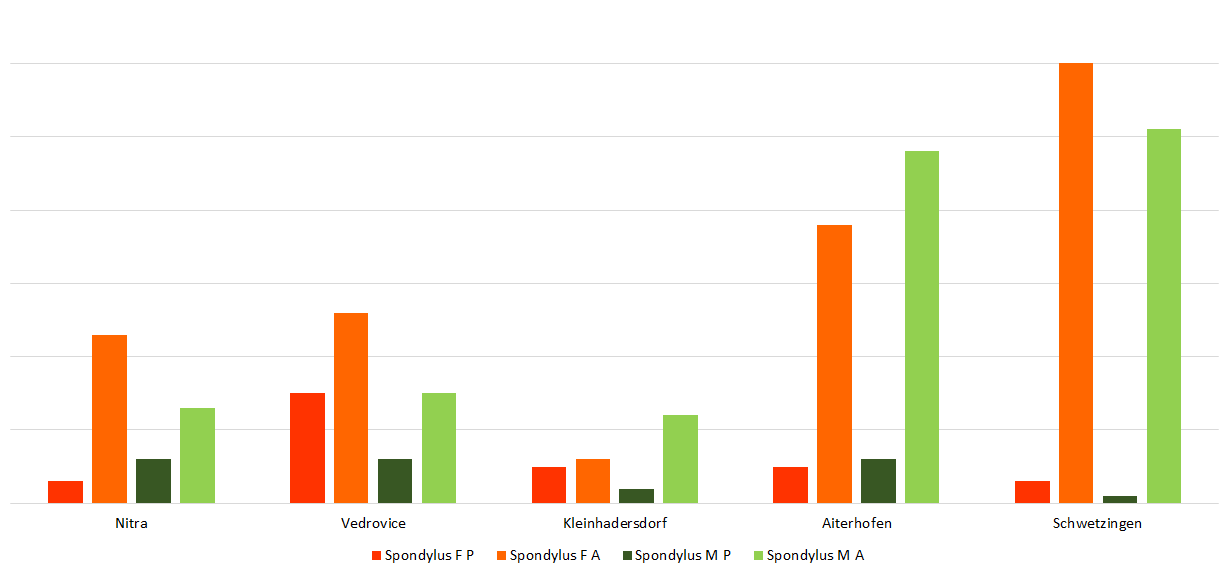


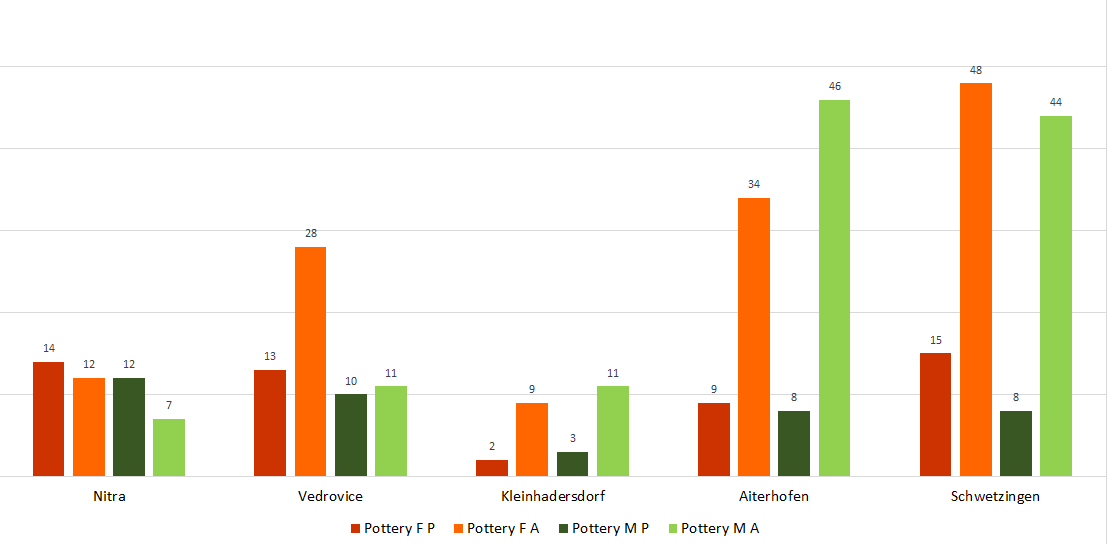
Figure 2: Pottery vessels distribution among sexes based in each cemetery.

Figure 3: Bone tools distribution among sexes based in each cemetery.


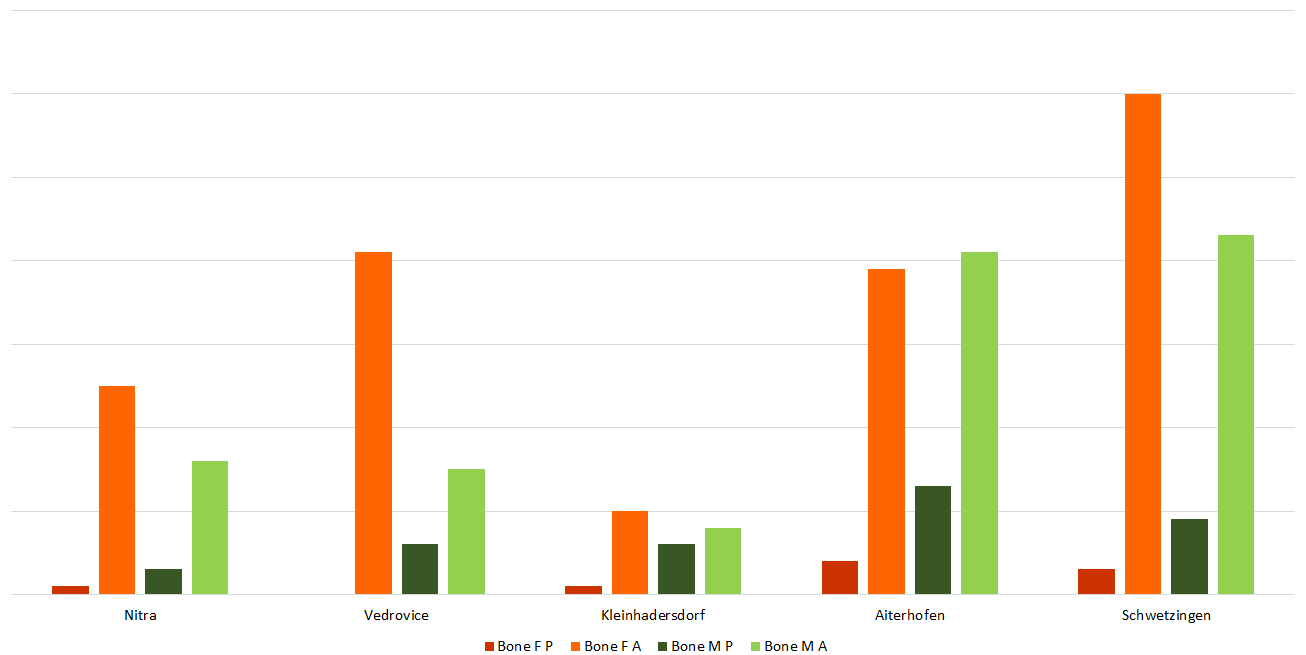

Supplement: S1 File — (DOCX) [file pone.0249130.s001.docx]
