# Supplementary material for: A sexual division of labour at the start of agriculture? A multi-proxy comparison through grave good stone tool technological and use-wear analysis
Source: PLoS One. 2021 Apr 14;16(4):e0249130. doi: 10.1371/journal.pone.0249130 (PMC8046253; doi:10.1371/journal.pone.0249130)
Supplement: S3 File — (DOCX) [file pone.0249130.s003.docx]

**Supporting** **Information 3**

The MCA statistics has been performed using “R” software (version 4.0.1.) (1), together with the R packages "FactoMineR" and "factoextra" (2, 3) and “ggplot2” (4).

1. **MCA Analysis: PBA technological atributes and use-wear analysis**

This database contained active individuals (rows 1:54) and active variables (columns 1:5 = Site, Section, Weight, HBI, use).

| Site | Section | Weigh groups | HBI Type | use |
| --- | --- | --- | --- | --- |
| Aiterhofen | flat-convex | heavy | Type 2 | butchering |
| Kleinhadersdorf | flat-convex | heavy | Type 2 | butchering |
| Vedrovice | flat-cylindrical | heavy | Type 3 | butchering |
| Vedrovice | flat-cylindrical | heavy | Type 3 | butchering |
| Vedrovice | flat-cylindrical | heavy | Type 3 | butchering |
| Vendenheim | flat-cylindrical | heavy | Type 1 | butchering |
| Nitra | triangular | heavy | Type 3 | butchering |
| Nitra | triangular | heavy | Type 3 | butchering |
| Vedrovice | triangular | heavy | Type 3 | butchering |
| Vedrovice | triangular | heavy | Type 3 | butchering |
| Kleinhadersdorf | U | heavy | Type 3 | butchering |
| Vedrovice | U | heavy | Type 3 | butchering |
| Vedrovice | U | heavy | Type 4 | butchering |
| Vendenheim | U | heavy | Type 4 | butchering |
| Vendenheim | U | heavy | Type 4 | butchering |
| Vedrovice | flat-convex | light | Type 2 | hide-work |
| Vedrovice | oval | heavy | Type 2 | hide-work |
| Vendenheim | oval | light | Type 1 | hide-work |
| Vendenheim | oval | light | Type 1 | hide-work |
| Aiterhofen | U | light | Type 1 | hide-work |
| Aiterhofen | flat-convex | light | Type 2 | soft |
| Schwetzingen | flat-convex | light | Type 2 | soft |
| Schwetzingen | flat-convex | light | Type 2 | soft |
| Vedrovice | flat-cylindrical | heavy | Type 3 | soft |
| Aiterhofen | U | light | Type 1 | soft |
| Aiterhofen | U | light | Type 1 | soft |
| Aiterhofen | flat-convex | heavy | Type 2 | soft animal tissues |
| Vedrovice | flat-convex | light | Type 2 | soft animal tissues |
| Kleinhadersdorf | oval | heavy | Type 2 | soft animal tissues |
| Vedrovice | triangular | heavy | Type 3 | soft animal tissues |
| Aiterhofen | U | light | Type 1 | soft animal tissues |
| Aiterhofen | U | heavy | Type 4 | soft animal tissues |
| Kleinhadersdorf | U | heavy | Type 3 | soft animal tissues |
| Schwetzingen | U | heavy | Type 3 | soft animal tissues |
| Aiterhofen | flat-convex | light | Type 2 | wood-work |
| Aiterhofen | flat-convex | light | Type 2 | wood-work |
| Vendenheim | flat-convex | light | Type 1 | wood-work |
| Nitra | flat-cylindrical | heavy | Type 3 | wood-work |
| Nitra | flat-cylindrical | heavy | Type 3 | wood-work |
| Aiterhofen | U | light | Type 1 | wood-work |
| Aiterhofen | U | light | Type 2 | wood-work |
| Aiterhofen | U | heavy | Type 4 | wood-work |
| Aiterhofen | U | heavy | Type 4 | wood-work |
| Aiterhofen | U | heavy | Type 4 | wood-work |
| Schwetzingen | U | heavy | Type 4 | wood-work |
| Vedrovice | U | heavy | Type 3 | wood-work |
| Vendenheim | U | light | Type 4 | wood-work |
| Schwetzingen | flat-convex | heavy | Type 2 | wood-work |
| Aiterhofen | flat-cylindrical | heavy | Type 2 | wood-work |
| Vendenheim | flat-cylindrical | heavy | Type 3 | wood-work |
| Aiterhofen | oval | light | Type 2 | wood-work |
| Vedrovice | triangular | heavy | Type 3 | wood-work |
| Aiterhofen | U | light | Type 1 | wood-work |
| Vedrovice | U | heavy | Type 4 | wood-work |

After selecting the variables that we wanted to be represented in the MCA we run the MCA()[FactoMiner package].

*Results*

*Eigenvalue*

eigenvalue variance.percent cumulative.variance.percent

Dim.1 0.68715991 22.905330 22.90533

Dim.2 0.44086308 14.695436 37.60077

Dim.3 0.39966646 13.322215 50.92298

Dim.4 0.31923954 10.641318 61.56430

Dim.5 0.28328685 9.442895 71.00719

Dim.6 0.23198466 7.732822 78.74002

Dim.7 0.20828490 6.942830 85.68285

Dim.8 0.15864647 5.288216 90.97106

Dim.9 0.10252963 3.417654 94.38872

Dim.10 0.07817348 2.605783 96.99450

Dim.11 0.05178335 1.726112 98.72061

Dim.12 0.03838167 1.279389 100.00000


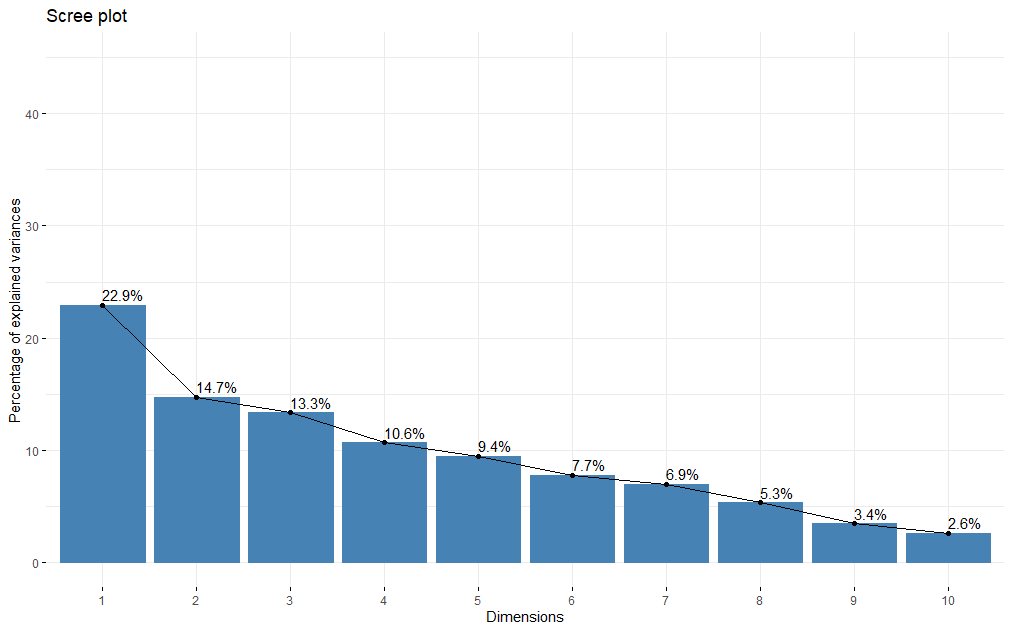


Multiple Correspondence Analysis Results for variables

===================================================

Name Description

1 "$coord" "Coordinates for categories"

2 "$cos2" "Cos2 for categories"

3 "$contrib" "contributions of categories"

> # Coordinates

> head(var$coord)

Dim 1 Dim 2 Dim 3 Dim 4 Dim 5

flat-convex 0.9338753 0.95758739 -1.009470368 0.24469569 0.01463786

flat-cylindrical -0.8733255 0.58943273 0.241518472 -1.04550521 -1.24055743

oval 1.2943360 0.14114397 1.964862515 1.18786356 -0.76847745

triangular -1.3106519 0.91327484 0.696414936 0.27123970 1.28092178

U -0.0888338 -1.04460510 -0.184673819 -0.04970715 0.32482814

heavy -0.6209433 0.03636398 0.005122216 0.20556239 -0.13132444

> # Cos2: quality on the factore map

> head(var$cos2)

Dim 1 Dim 2 Dim 3 Dim 4 Dim 5

flat-convex 0.249178046 0.261992460 0.2911515497 0.017107424 6.121911e-05

flat-cylindrical 0.152539487 0.069486188 0.0116662345 0.218616229 3.077965e-01

oval 0.170949563 0.002032818 0.3939474186 0.143981615 6.026098e-02

triangular 0.214726035 0.104258867 0.0606242203 0.009196372 2.050951e-01

U 0.005425368 0.750199867 0.0234467884 0.001698676 7.254041e-02

heavy 0.710261543 0.002435888 0.0000483315 0.077839805 3.176915e-02

> # Contributions to the principal components

> head(var$contrib)

Dim 1 Dim 2 Dim 3 Dim 4 Dim 5

flat-convex 7.0509478 11.55528340 14.165011661 1.04198982 0.004202001

flat-cylindrical 4.6246964 3.28362472 0.608123456 14.26673142 22.635812748

oval 5.6435517 0.10460132 22.360570460 10.23134416 4.825608533

triangular 6.9440746 5.25529086 3.370822963 0.64015822 16.088527779

U 0.1169681 25.20980458 0.869124280 0.07882973 3.793584925

heavy 9.0920190 0.04860192 0.001063732 2.14479068 0.986458911

We create a MCA plot showing the quality of representation of variable categories. The quality of the representation is called the squared cosine (cos2), which measures the degree of association between variable categories and a particular axis. If a variable category is well represented by two dimensions, the sum of the cos2 is closed to one. Using the squared cosine (cos2) we colour the variable categories by their cos2 values using the argument col.var = “cos2”. This produces a gradient colours.


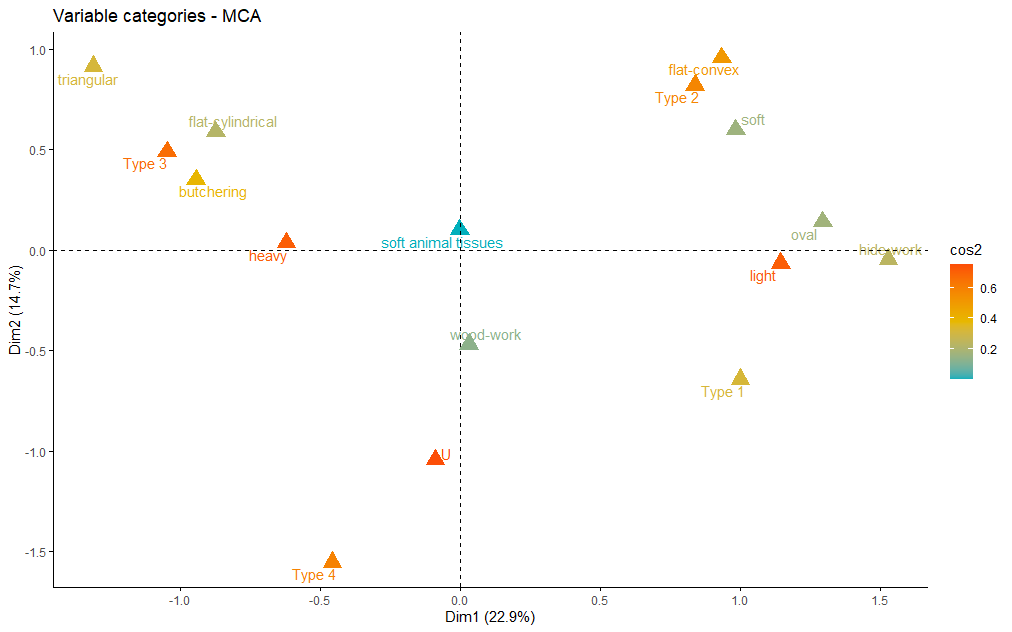


# MCA Analysis: PBA activities and sexed skeletons.

This database contained active individuals (rows 1:64) and active variables (columns 1:6 = Site, Sex, Section, Weight, HBI, use).

| Site | Sex | Section | Weight | HBI | use |
| --- | --- | --- | --- | --- | --- |
| Aiterhofen | m | flat-convex | intermediate | type 2 | butchering |
| Kleinhadersdorf | m | flat-convex | intermediate | type 3 | butchering |
| Vedrovice | m | flat-cylindrical | high | type 3 | butchering |
| Vedrovice | m | flat-cylindrical | high | type 3 | butchering |
| Vedrovice | m | flat-cylindrical | high | type 3 | butchering |
| Vedrovice | m | triangular | high | type 3 | butchering |
| Vedrovice | m | triangular | high | type 3 | butchering |
| Nitra | m | triangular | high | type 3 | butchering |
| Nitra | m | triangular | high | type 3 | butchering |
| Kleinhadersdorf | m | U | intermediate | type 3 | butchering |
| Vedrovice | m | U | high | type 3 | butchering |
| Vendenheim | m | U | intermediate | type 4 | butchering |
| Vedrovice | m | U | intermediate | type 4 | butchering |
| Vendenheim | non-adult | U | intermediate | type 4 | butchering |
| Aiterhofen | m | flat-convex | light | type 2 | hard material |
| Schwetzingen | m | flat-convex | high | type 2 | hard material |
| Schwetzingen | m | flat-convex | intermediate | type 2 | hard material |
| Aiterhofen | m | flat-convex | high | type 4 | hard material |
| Vendenheim | m | flat-cylindrical | high | type 3 | hard material |
| Aiterhofen | m | oval | intermediate | type 2 | hard material |
| Vedrovice | m | triangular | intermediate | type 3 | hard material |
| Aiterhofen | m | U | light | type 1 | hard material |
| Aiterhofen | m | U | intermediate | type 1 | hard material |
| Aiterhofen | m | U | light | type 2 | hard material |
| Kleinhadersdorf | m | U | intermediate | type 3 | hard material |
| Kleinhadersdorf | m | U | high | type 3 | hard material |
| Nitra | m | U | high | type 3 | hard material |
| Kleinhadersdorf | m | U | intermediate | type 4 | hard material |
| Vedrovice | m | U | intermediate | type 4 | hard material |
| Vedrovice | m | U | high | type 4 | hard material |
| Kleinhadersdorf | non-adult | flat-convex | light | type 2 | hard material |
| Schwetzingen | non-adult | flat-convex | light | type 2 | hard material |
| Kleinhadersdorf | non-adult | flat-convex | light | type 2 | hard material |
| Aiterhofen | non-adult | flat-convex | light | type 2 | hard material |
| Kleinhadersdorf | non-adult | flat-convex | high | type 2 | hard material |
| Vendenheim | non-adult | flat-cylindrical | light | type 1 | hard material |
| Vedrovice | f | oval | light | type 2 | hide-work |
| Aiterhofen | m | U | light | type 1 | hide-work |
| Vedrovice | non-adult | flat-convex | light | type 2 | hide-work |
| Vendenheim | non-adult | oval | light | type 1 | hide-work |
| Vendenheim | non-adult | oval | light | type 1 | hide-work |
| Vedrovice | f | flat-convex | light | type 2 | soft |
| Aiterhofen | f | flat-convex | light | type 2 | soft |
| Aiterhofen | m | flat-convex | light | type 2 | soft |
| Schwetzingen | m | flat-convex | light | type 2 | soft |
| Schwetzingen | m | flat-convex | light | type 2 | soft |
| Vedrovice | m | flat-cylindrical | high | type 3 | soft |
| Aiterhofen | m | U | light | type 1 | soft |
| Aiterhofen | m | U | light | type 1 | soft |
| Aiterhofen | m | U | intermediate | type 4 | soft |
| Aiterhofen | non-adult | U | light | type 1 | soft |
| Vendenheim | m | flat-convex | light | type 1 | wood-work |
| Aiterhofen | m | flat-convex | light | type 2 | wood-work |
| Nitra | m | flat-cylindrical | high | type 3 | wood-work |
| Nitra | m | flat-cylindrical | high | type 3 | wood-work |
| Aiterhofen | m | U | light | type 1 | wood-work |
| Aiterhofen | m | U | light | type 2 | wood-work |
| Vedrovice | m | U | high | type 3 | wood-work |
| Schwetzingen | m | U | high | type 3 | wood-work |
| Vendenheim | m | U | light | type 4 | wood-work |
| Aiterhofen | m | U | intermediate | type 4 | wood-work |
| Aiterhofen | m | U | intermediate | type 4 | wood-work |
| Aiterhofen | m | U | intermediate | type 4 | wood-work |
| Schwetzingen | m | flat-convex | intermediate | type 3 | wood-work |

After selecting those variables that we wanted to be represented in the MCA we run the MCA()[FactoMiner package].

*Results*

Eigenvalue:

**## eigenvalue variance.percent cumulative.variance.percent**## Dim.1 0.64483973 21.4946577 21.49466
## Dim.2 0.40953980 13.6513266 35.14598
## Dim.3 0.35489490 11.8298299 46.97581
## Dim.4 0.27706421 9.2354738 56.21129
## Dim.5 0.26121049 8.7070162 64.91830
## Dim.6 0.21063008 7.0210027 71.93931
## Dim.7 0.17595713 5.8652376 77.80454
## Dim.8 0.15299060 5.0996866 82.90423
## Dim.9 0.11939053 3.9796844 86.88392
## Dim.10 0.09146938 3.0489794 89.93289
## Dim.11 0.08884142 2.9613807 92.89428
## Dim.12 0.07181782 2.3939272 95.28820
## Dim.13 0.06248003 2.0826677 97.37087
## Dim.14 0.05384082 1.7946940 99.16556
## Dim.15 0.02503307 0.8344355 100.00000


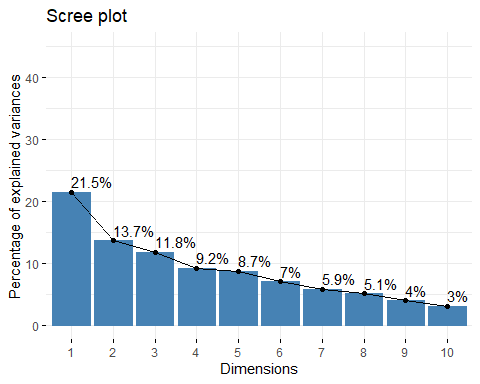


## Multiple Correspondence Analysis Results for variables
## ===================================================
## Name Description
## 1 "$coord" "Coordinates for categories"
## 2 "$cos2" "Cos2 for categories"
## 3 "$contrib" "contributions of categories"

The different components can be accessed as follow:

# Coordinates
head(var$coord)

## Dim 1 Dim 2 Dim 3 Dim 4 Dim 5
## f 1.8240632 0.9375284 -0.65063536 -0.4923001 2.97139885
## m -0.3635205 -0.1252988 -0.04354812 -0.1226429 0.04073786
## non-adult 1.1548942 0.3138504 0.37539199 0.6917315 -0.99555358
## flat-convex 0.7063528 0.2446659 -1.01829924 0.2269884 -0.12834945
## flat-cylindrical -0.9084291 1.1946717 0.30676984 -0.9522334 -0.74875962
## oval 1.7292172 0.5940992 2.28999979 1.0769027 0.88327605

# Cos2: quality on the factore map
head(var$cos2)

## Dim 1 Dim 2 Dim 3 Dim 4 Dim 5
## f 0.1636331 0.04322752 0.020819330 0.01191931 0.434223498
## m 0.4719542 0.05607066 0.006772994 0.05371889 0.005927046
## non-adult 0.2768224 0.02044382 0.029247370 0.09930977 0.205705589
## flat-convex 0.2267883 0.02720973 0.471333335 0.02341988 0.007487991
## flat-cylindrical 0.1178919 0.20389148 0.013443962 0.12953549 0.080091567
## oval 0.1993461 0.02353026 0.349606602 0.07731463 0.052011772

# Contributions to the principal components

## Dim 1 Dim 2 Dim 3 Dim 4 Dim 5
## f 4.837258 2.0120744 1.11827043 0.8200695 31.68856471
## m 3.202035 0.5989873 0.08349472 0.8482515 0.09927177
## non-adult 7.110094 0.8267837 1.36493808 5.9365948 13.04311649
## flat-convex 4.835836 0.9135469 18.26127505 1.1622699 0.39416440
## flat-cylindrical 3.199413 8.7124645 0.66292679 8.1817532 5.36579691
## oval 5.796386 1.0772881 18.47060588 5.2321778 3.73346697

We create a MCA plot showing the quality of representation of variable categories. The quality of the representation is called the squared cosine (cos2), which measures the degree of association between variable categories and a particular axis. If a variable category is well represented by two dimensions, the sum of the cos2 is closed to one. Using the squared cosine (cos2) we colour the variable categories by their cos2 values using the argument col.var = “cos2”. This produces a gradient colours.


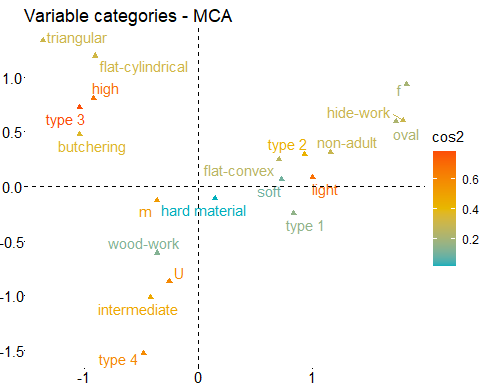


We also created a plot colouring individuals by groups visualising “Sex”:


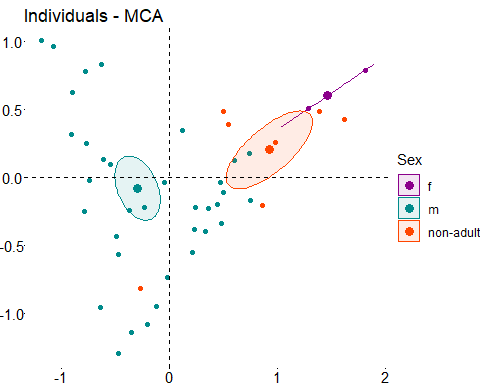


We created a plot colouring individuals by groups visualising all groups:


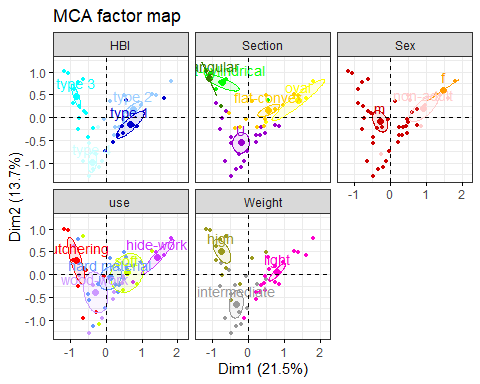


# MCA Analysis: a multi-proxy comparison

- 1. **MCA excluding the mobility data.**

In MCA terminology, our data contains active individuals (rows 1:366) and active variables (columns 1:10= Site, Unfurnished, PBA, Pottery, Spondylus, Flaked, Projectile, Bone, Sex, Diet).

| Site | Unfurnished | PBA | Pottery | Spondylus | Flaked | Projectile | Bone | Sex | Diet |
| --- | --- | --- | --- | --- | --- | --- | --- | --- | --- |
| Vedrovice | furnished | A | P | P | A | A | A | female | high |
| Vedrovice | furnished | A | A | P | A | A | A | female | high |
| Vedrovice | furnished | A | A | P | P | A | A | female | high |
| Vedrovice | unfurnished | A | A | A | A | A | A | female | high |
| Vedrovice | unfurnished | A | A | A | A | A | A | female | high |
| Vedrovice | furnished | P | A | A | A | A | A | female | high |
| Schwetzingen | furnished | A | A | A | A | A | P | female | high |
| Kleinhadersdorf | furnished | A | A | P | A | A | A | female | high |
| Nitra | furnished | A | P | P | A | A | A | female | high |
| Nitra | furnished | A | P | P | A | A | A | female | high |
| Nitra | furnished | A | P | A | A | A | A | female | high |
| Nitra | furnished | A | P | A | A | A | A | female | high |
| Nitra | furnished | A | P | A | A | A | A | female | high |
| Nitra | furnished | A | P | A | A | A | A | female | high |
| Nitra | furnished | A | P | A | A | A | A | female | high |
| Nitra | furnished | A | P | A | A | A | A | female | high |
| Nitra | furnished | A | P | A | A | A | A | female | high |
| Nitra | unfurnished | A | A | A | A | A | A | female | high |
| Nitra | unfurnished | A | A | A | A | A | A | female | high |
| Schwetzingen | furnished | A | P | A | A | A | A | female | high |
| Kleinhadersdorf | unfurnished | A | A | A | A | A | A | female | high |
| Kleinhadersdorf | unfurnished | A | A | A | A | A | A | female | high |
| Schwetzingen | unfurnished | A | A | A | A | A | A | female | high |
| Vendenheim | unfurnished | A | A | A | A | A | A | female | high |
| Schwetzingen | furnished | A | A | A | P | A | A | female | high |
| Schwetzingen | furnished | A | A | A | A | A | P | female | high |
| Schwetzingen | unfurnished | A | A | A | A | A | A | female | high |
| Nitra | furnished | A | A | A | A | A | A | male | high |
| Kleinhadersdorf | furnished | P | A | P | P | P | P | male | high |
| Kleinhadersdorf | furnished | A | A | A | A | A | A | male | high |
| Aiterhofen | furnished | P | A | P | A | A | A | male | high |
| Aiterhofen | furnished | P | A | P | A | A | A | male | high |
| Kleinhadersdorf | furnished | P | P | A | A | P | P | male | high |
| Aiterhofen | furnished | P | A | P | P | A | P | male | high |
| Vedrovice | furnished | P | P | A | A | A | P | male | high |
| Vedrovice | furnished | P | P | P | A | A | A | male | high |
| Vedrovice | furnished | P | P | P | A | P | A | male | high |
| Vedrovice | unfurnished | A | A | A | A | A | A | male | high |
| Vedrovice | unfurnished | A | A | A | A | A | A | male | high |
| Vedrovice | unfurnished | A | A | A | A | A | A | male | high |
| Vedrovice | furnished | P | A | A | A | P | A | male | high |
| Vedrovice | furnished | P | A | A | A | P | A | male | high |
| Vendenheim | furnished | A | P | A | A | P | A | male | high |
| Vedrovice | furnished | P | P | P | A | P | P | male | high |
| Kleinhadersdorf | furnished | P | P | A | P | P | P | male | high |
| Kleinhadersdorf | furnished | P | A | A | A | P | A | male | high |
| Schwetzingen | furnished | A | A | A | A | A | P | male | high |
| Kleinhadersdorf | furnished | A | P | A | A | P | P | male | high |
| Vendenheim | furnished | P | A | A | P | A | P | male | high |
| Vendenheim | furnished | P | A | A | P | P | P | male | high |
| Nitra | furnished | P | P | P | P | A | P | male | high |
| Schwetzingen | furnished | A | A | A | P | P | P | male | high |
| Aiterhofen | furnished | P | A | A | A | P | A | male | high |
| Nitra | furnished | P | P | P | A | A | A | male | high |
| Nitra | furnished | P | P | P | A | A | A | male | high |
| Nitra | furnished | P | P | P | A | A | A | male | high |
| Nitra | furnished | A | P | A | A | A | A | male | high |
| Nitra | unfurnished | A | A | A | A | A | A | male | high |
| Nitra | unfurnished | A | A | A | A | A | A | male | high |
| Nitra | unfurnished | A | A | A | A | A | A | male | high |
| Nitra | furnished | P | A | A | A | A | A | male | high |
| Vendenheim | unfurnished | A | A | A | A | A | A | male | high |
| Kleinhadersdorf | furnished | P | A | A | A | A | A | male | high |
| Vendenheim | furnished | P | A | A | A | P | A | male | high |
| Schwetzingen | unfurnished | A | A | A | A | A | A | male | high |
| Schwetzingen | furnished | A | A | P | A | A | A | female | low |
| Vedrovice | furnished | P | P | P | A | A | A | female | low |
| Vedrovice | furnished | A | A | P | A | A | A | female | low |
| Vedrovice | furnished | A | P | P | A | A | A | female | low |
| Vedrovice | furnished | A | P | A | A | A | A | female | low |
| Vedrovice | furnished | A | P | A | A | A | A | female | low |
| Vedrovice | unfurnished | A | A | A | A | A | A | female | low |
| Vedrovice | unfurnished | A | A | A | A | A | A | female | low |
| Vedrovice | unfurnished | A | A | A | A | A | A | female | low |
| Vedrovice | unfurnished | A | A | A | A | A | A | female | low |
| Vedrovice | unfurnished | A | A | A | A | A | A | female | low |
| Vedrovice | unfurnished | A | A | A | A | A | A | female | low |
| Vedrovice | unfurnished | A | A | A | A | A | A | female | low |
| Vedrovice | furnished | A | P | A | P | A | A | female | low |
| Vedrovice | unfurnished | A | A | A | A | A | A | female | low |
| Vedrovice | unfurnished | A | A | A | A | A | A | female | low |
| Kleinhadersdorf | furnished | A | P | P | P | A | A | female | low |
| Nitra | furnished | A | A | A | P | A | P | female | low |
| Kleinhadersdorf | furnished | A | A | P | A | A | P | female | low |
| Kleinhadersdorf | furnished | A | P | P | A | A | A | female | low |
| Schwetzingen | furnished | A | A | P | A | A | A | female | low |
| Nitra | furnished | A | P | A | A | A | A | female | low |
| Nitra | furnished | A | P | A | A | A | A | female | low |
| Nitra | furnished | A | P | A | A | A | A | female | low |
| Nitra | unfurnished | A | A | A | A | A | A | female | low |
| Nitra | unfurnished | A | A | A | A | A | A | female | low |
| Nitra | unfurnished | A | A | A | A | A | A | female | low |
| Nitra | unfurnished | A | A | A | A | A | A | female | low |
| Nitra | unfurnished | A | A | A | A | A | A | female | low |
| Nitra | furnished | P | P | A | A | A | A | female | low |
| Schwetzingen | furnished | A | P | A | A | A | A | female | low |
| Schwetzingen | furnished | A | P | A | A | A | A | female | low |
| Schwetzingen | furnished | A | A | A | A | A | A | female | low |
| Aiterhofen | unfurnished | A | A | A | A | A | A | female | low |
| Kleinhadersdorf | unfurnished | A | A | A | A | A | A | female | low |
| Kleinhadersdorf | unfurnished | A | A | A | A | A | A | female | low |
| Kleinhadersdorf | unfurnished | A | A | A | A | A | A | female | low |
| Schwetzingen | unfurnished | A | A | A | A | A | A | female | low |
| Schwetzingen | unfurnished | A | A | A | A | A | A | female | low |
| Schwetzingen | unfurnished | A | A | A | A | A | A | female | low |
| Schwetzingen | unfurnished | A | A | A | A | A | A | female | low |
| Vendenheim | unfurnished | A | A | A | A | A | A | female | low |
| Aiterhofen | furnished | A | P | A | P | A | A | female | low |
| Nitra | furnished | A | P | P | A | A | A | female | low |
| Nitra | furnished | A | A | P | A | A | A | female | low |
| Nitra | unfurnished | A | A | A | A | A | A | female | low |
| Schwetzingen | furnished | A | P | A | A | A | A | female | low |
| Schwetzingen | unfurnished | A | A | A | A | A | A | female | low |
| Vedrovice | furnished | P | P | P | A | P | P | male | low |
| Kleinhadersdorf | furnished | A | A | A | A | A | P | male | low |
| Kleinhadersdorf | furnished | A | A | A | A | A | A | male | low |
| Kleinhadersdorf | furnished | A | A | A | A | A | A | male | low |
| Aiterhofen | furnished | A | A | A | P | A | P | male | low |
| Nitra | furnished | A | P | A | P | A | P | male | low |
| Kleinhadersdorf | furnished | P | A | P | A | A | P | male | low |
| Schwetzingen | furnished | A | A | A | A | A | P | male | low |
| Nitra | furnished | A | P | A | A | A | A | male | low |
| Kleinhadersdorf | unfurnished | A | A | A | A | A | A | male | low |
| Schwetzingen | furnished | A | A | A | P | A | A | male | low |
| Kleinhadersdorf | furnished | P | A | A | A | A | A | male | low |
| Nitra | furnished | A | P | A | A | A | P | male | low |
| Nitra | furnished | A | P | A | A | A | A | male | low |
| Aiterhofen | furnished | A | A | A | A | A | A | female | medium |
| Aiterhofen | furnished | A | A | A | A | A | A | female | medium |
| Aiterhofen | furnished | A | A | A | A | A | A | female | medium |
| Schwetzingen | furnished | A | A | A | A | A | A | female | medium |
| Schwetzingen | furnished | A | A | A | A | A | P | female | medium |
| Aiterhofen | furnished | A | A | P | A | A | A | female | medium |
| Vedrovice | furnished | A | P | P | A | A | A | female | medium |
| Schwetzingen | furnished | A | P | A | A | A | A | female | medium |
| Vedrovice | furnished | A | A | A | A | A | A | female | medium |
| Vedrovice | furnished | A | P | P | A | A | A | female | medium |
| Vedrovice | furnished | A | P | P | A | A | A | female | medium |
| Vedrovice | furnished | A | A | P | A | A | A | female | medium |
| Vedrovice | furnished | A | A | P | P | A | A | female | medium |
| Vedrovice | furnished | A | P | P | A | A | A | female | medium |
| Vedrovice | furnished | A | P | P | A | A | A | female | medium |
| Vedrovice | furnished | A | A | P | A | A | A | female | medium |
| Vedrovice | furnished | A | P | A | A | A | A | female | medium |
| Vedrovice | furnished | A | P | A | A | A | A | female | medium |
| Vedrovice | unfurnished | A | A | A | A | A | A | female | medium |
| Vedrovice | unfurnished | A | A | A | A | A | A | female | medium |
| Vedrovice | unfurnished | A | A | A | A | A | A | female | medium |
| Vedrovice | unfurnished | A | A | A | A | A | A | female | medium |
| Vedrovice | unfurnished | A | A | A | A | A | A | female | medium |
| Vedrovice | unfurnished | A | A | A | A | A | A | female | medium |
| Vedrovice | unfurnished | A | A | A | A | A | A | female | medium |
| Vedrovice | furnished | A | A | P | A | A | A | female | medium |
| Aiterhofen | furnished | P | A | P | P | A | P | female | medium |
| Aiterhofen | furnished | A | P | P | P | A | A | female | medium |
| Aiterhofen | furnished | A | A | A | P | A | P | female | medium |
| Aiterhofen | furnished | A | A | P | A | A | P | female | medium |
| Schwetzingen | furnished | A | A | A | A | A | A | female | medium |
| Schwetzingen | furnished | A | A | A | P | A | A | female | medium |
| Aiterhofen | furnished | P | P | A | P | A | P | female | medium |
| Schwetzingen | furnished | A | P | A | A | A | P | female | medium |
| Schwetzingen | furnished | A | A | P | A | A | A | female | medium |
| Aiterhofen | furnished | A | P | A | A | A | A | female | medium |
| Aiterhofen | furnished | A | P | A | A | A | A | female | medium |
| Schwetzingen | furnished | A | P | A | A | A | A | female | medium |
| Schwetzingen | furnished | A | P | A | A | A | A | female | medium |
| Schwetzingen | furnished | A | P | A | A | A | A | female | medium |
| Schwetzingen | furnished | A | P | A | A | A | A | female | medium |
| Schwetzingen | furnished | A | P | A | A | A | A | female | medium |
| Schwetzingen | furnished | A | P | A | A | A | A | female | medium |
| Aiterhofen | unfurnished | A | A | A | A | A | A | female | medium |
| Schwetzingen | furnished | A | A | A | A | A | A | female | medium |
| Aiterhofen | unfurnished | A | A | A | A | A | A | female | medium |
| Aiterhofen | unfurnished | A | A | A | A | A | A | female | medium |
| Aiterhofen | unfurnished | A | A | A | A | A | A | female | medium |
| Aiterhofen | unfurnished | A | A | A | A | A | A | female | medium |
| Aiterhofen | unfurnished | A | A | A | A | A | A | female | medium |
| Aiterhofen | unfurnished | A | A | A | A | A | A | female | medium |
| Aiterhofen | unfurnished | A | A | A | A | A | A | female | medium |
| Schwetzingen | unfurnished | A | A | A | A | A | A | female | medium |
| Schwetzingen | unfurnished | A | A | A | A | A | A | female | medium |
| Schwetzingen | unfurnished | A | A | A | A | A | A | female | medium |
| Schwetzingen | unfurnished | A | A | A | A | A | A | female | medium |
| Schwetzingen | unfurnished | A | A | A | A | A | A | female | medium |
| Schwetzingen | unfurnished | A | A | A | A | A | A | female | medium |
| Schwetzingen | unfurnished | A | A | A | A | A | A | female | medium |
| Schwetzingen | unfurnished | A | A | A | A | A | A | female | medium |
| Schwetzingen | unfurnished | A | A | A | A | A | A | female | medium |
| Schwetzingen | unfurnished | A | A | A | A | A | A | female | medium |
| Schwetzingen | unfurnished | A | A | A | A | A | A | female | medium |
| Schwetzingen | unfurnished | A | A | A | A | A | A | female | medium |
| Schwetzingen | unfurnished | A | A | A | A | A | A | female | medium |
| Schwetzingen | unfurnished | A | A | A | A | A | A | female | medium |
| Aiterhofen | furnished | A | P | A | A | P | A | female | medium |
| Schwetzingen | furnished | A | P | A | A | A | A | female | medium |
| Schwetzingen | unfurnished | A | A | A | A | A | A | female | medium |
| Schwetzingen | unfurnished | A | A | A | A | A | A | female | medium |
| Schwetzingen | unfurnished | A | A | A | A | A | A | female | medium |
| Schwetzingen | unfurnished | A | A | A | A | A | A | female | medium |
| Schwetzingen | unfurnished | A | A | A | A | A | A | female | medium |
| Schwetzingen | unfurnished | A | A | A | A | A | A | female | medium |
| Schwetzingen | furnished | A | P | A | P | P | A | female | medium |
| Aiterhofen | furnished | P | A | P | P | A | P | male | medium |
| Vedrovice | furnished | P | P | P | P | A | A | male | medium |
| Schwetzingen | furnished | A | A | A | A | A | P | male | medium |
| Schwetzingen | furnished | A | A | A | P | A | P | male | medium |
| Schwetzingen | furnished | A | A | A | A | A | A | male | medium |
| Schwetzingen | furnished | P | P | A | P | A | A | male | medium |
| Aiterhofen | furnished | P | A | A | A | P | A | male | medium |
| Vedrovice | furnished | P | P | P | P | P | A | male | medium |
| Schwetzingen | furnished | P | A | A | A | A | A | male | medium |
| Vedrovice | furnished | P | P | A | A | A | P | male | medium |
| Vedrovice | furnished | P | A | A | A | A | P | male | medium |
| Vedrovice | furnished | P | A | A | P | A | P | male | medium |
| Vedrovice | furnished | A | P | A | A | A | A | male | medium |
| Vedrovice | unfurnished | A | A | A | A | A | A | male | medium |
| Vedrovice | unfurnished | A | A | A | A | A | A | male | medium |
| Vedrovice | unfurnished | A | A | A | A | A | A | male | medium |
| Vedrovice | unfurnished | A | A | A | A | A | A | male | medium |
| Vedrovice | furnished | A | P | A | P | P | A | male | medium |
| Aiterhofen | furnished | P | A | A | P | P | A | male | medium |
| Aiterhofen | furnished | P | A | A | A | A | A | male | medium |
| Aiterhofen | furnished | P | A | A | A | A | A | male | medium |
| Schwetzingen | furnished | A | A | A | A | A | P | male | medium |
| Schwetzingen | furnished | P | P | A | A | P | A | male | medium |
| Aiterhofen | furnished | A | A | A | A | A | P | male | medium |
| Aiterhofen | furnished | A | A | A | A | A | P | male | medium |
| Vendenheim | furnished | A | A | A | A | A | P | male | medium |
| Schwetzingen | furnished | P | A | A | A | P | P | male | medium |
| Schwetzingen | furnished | A | A | A | A | A | P | male | medium |
| Schwetzingen | furnished | P | A | A | A | P | P | male | medium |
| Schwetzingen | furnished | A | P | P | A | A | P | male | medium |
| Aiterhofen | furnished | A | A | A | A | A | P | male | medium |
| Schwetzingen | furnished | A | A | A | P | P | P | male | medium |
| Schwetzingen | furnished | P | A | A | A | A | P | male | medium |
| Schwetzingen | furnished | A | A | A | A | A | P | male | medium |
| Schwetzingen | furnished | A | A | A | P | A | P | male | medium |
| Schwetzingen | furnished | A | A | A | P | A | P | male | medium |
| Aiterhofen | furnished | A | A | A | P | A | P | male | medium |
| Aiterhofen | furnished | P | P | A | P | A | A | male | medium |
| Aiterhofen | furnished | A | P | A | A | A | A | male | medium |
| Schwetzingen | furnished | A | P | A | A | A | A | male | medium |
| Schwetzingen | furnished | A | P | A | A | A | A | male | medium |
| Aiterhofen | furnished | A | P | A | A | A | A | male | medium |
| Aiterhofen | furnished | A | A | A | A | A | A | male | medium |
| Schwetzingen | furnished | A | A | A | A | A | A | male | medium |
| Aiterhofen | unfurnished | A | A | A | A | A | A | male | medium |
| Aiterhofen | unfurnished | A | A | A | A | A | A | male | medium |
| Schwetzingen | unfurnished | A | A | A | A | A | A | male | medium |
| Schwetzingen | unfurnished | A | A | A | A | A | A | male | medium |
| Schwetzingen | unfurnished | A | A | A | A | A | A | male | medium |
| Schwetzingen | furnished | A | A | A | A | P | A | male | medium |
| Schwetzingen | furnished | A | A | A | A | P | A | male | medium |
| Schwetzingen | furnished | A | P | A | P | P | A | male | medium |
| Aiterhofen | furnished | A | A | A | P | P | A | male | medium |
| Aiterhofen | furnished | P | A | A | A | A | A | male | medium |
| Aiterhofen | furnished | P | A | A | A | A | A | male | medium |
| Schwetzingen | furnished | P | A | A | A | A | A | male | medium |
| Schwetzingen | furnished | P | A | A | A | A | A | male | medium |
| Schwetzingen | furnished | P | A | A | A | A | A | male | medium |
| Aiterhofen | furnished | P | P | A | P | A | A | male | medium |
| Schwetzingen | furnished | P | A | A | P | A | A | male | medium |
| Schwetzingen | furnished | P | A | A | A | P | A | male | medium |
| Schwetzingen | furnished | P | P | A | P | A | P | male | medium |
| Schwetzingen | unfurnished | A | A | A | A | A | A | male | medium |
| Schwetzingen | unfurnished | A | A | A | A | A | A | male | medium |
| Schwetzingen | unfurnished | A | A | A | A | A | A | male | medium |
| Schwetzingen | unfurnished | A | A | A | A | A | A | male | medium |
| Schwetzingen | unfurnished | A | A | A | A | A | A | male | medium |
| Schwetzingen | unfurnished | A | A | A | A | A | A | male | medium |
| Schwetzingen | unfurnished | A | A | A | A | A | A | male | medium |
| Schwetzingen | unfurnished | A | A | A | A | A | A | male | medium |
| Schwetzingen | unfurnished | A | A | A | A | A | A | male | medium |
| Schwetzingen | furnished | A | A | A | P | A | A | male | medium |
| Schwetzingen | furnished | A | A | A | A | P | A | male | medium |

## 2.2. Data description using furnished and unfurnished burials.

First, we select those variables that we want to be represented in the MCA (all but the site: columns 3-10) and we use the function MCA()[FactoMiner package]

### *Results*

Eigenvalue:

eigenvalue variance.percent cumulative.variance.percent

Dim.1 0.28720443 25.529283 25.52928

Dim.2 0.17316026 15.392023 40.92131

Dim.3 0.13997009 12.441785 53.36309

Dim.4 0.11551365 10.267880 63.63097

Dim.5 0.10687298 9.499821 73.13079

Dim.6 0.09071772 8.063797 81.19459

Dim.7 0.08421793 7.486038 88.68063

Dim.8 0.07294489 6.483991 95.16462


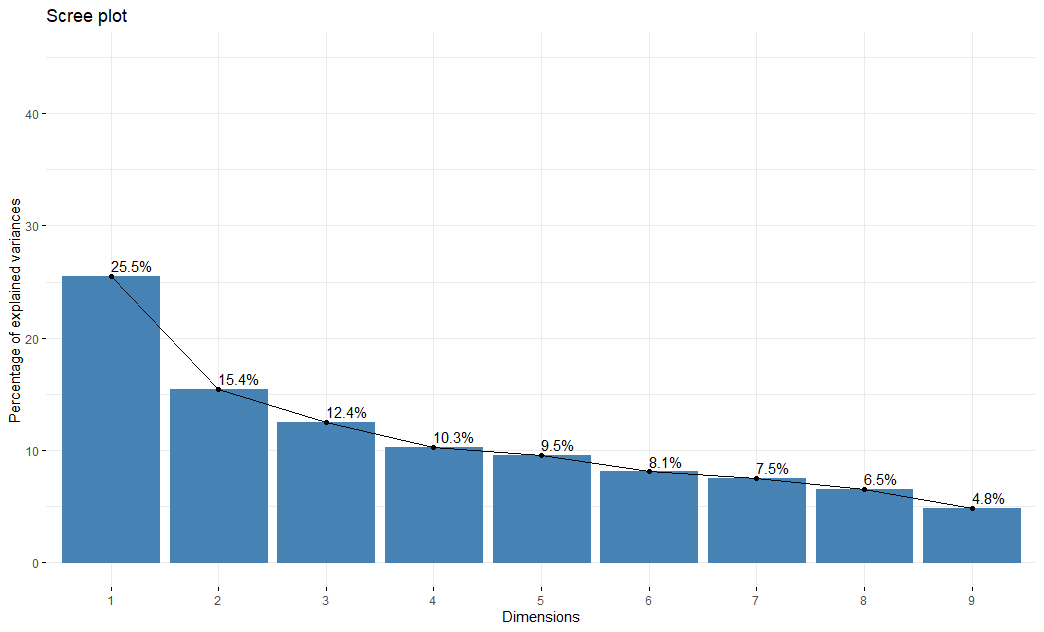
Dim.9 0.05439804 4.835381 100.00000

## Multiple Correspondence Analysis Results for variables
## ===================================================
## Name Description
## 1 "$coord" "Coordinates for categories"
## 2 "$cos2" "Cos2 for categories"
## 3 "$contrib" "contributions of categories"

> # Coordinates

> (var$coord)

Dim 1 Dim 2 Dim 3 Dim 4 Dim 5

PBA_A -0.38206552 -0.066991219 0.08010487 -0.02181572 0.03909018

PBA_P 1.46123303 0.256212031 -0.30636599 0.08343557 -0.14950280

Pottery_A -0.08308546 -0.439043589 -0.02685367 0.29054603 -0.24980954

Pottery_P 0.19219769 1.015618905 0.06211934 -0.67210647 0.57787267

Spondylus_A -0.07636478 -0.327695223 -0.05552897 0.02652857 0.23105148

Spondylus_P 0.37045041 1.589670444 0.26937457 -0.12869178 -1.12084547

Flaked_A -0.22229502 -0.021034047 -0.25579690 0.04711279 -0.03178827

Flaked_P 1.23320809 0.116688881 1.41906378 -0.26136380 0.17634921

Projectile_A -0.22267781 0.009950844 0.09066379 0.03614296 -0.16509620

Projectile_P 1.75268985 -0.078322773 -0.71361175 -0.28448009 1.29946689

Bone_A -0.26945786 0.058458730 -0.23359156 -0.14661453 0.06792608

Bone_P 1.18350121 -0.256759914 1.02597077 0.64395403 -0.29834200

female -0.66450481 0.294284290 0.11708585 -0.07885770 -0.01995201

male 0.79740577 -0.353141147 -0.14050302 0.09462924 0.02394241

high 0.59589084 0.569727569 -1.08058868 0.58244981 -0.30931949

low -0.65878493 0.570961076 0.65744800 1.06253837 0.83601609

medium 0.01426866 -0.489404586 0.19916546 -0.70092309 -0.21437318

> # Cos2: quality on the factore map

> (var$cos2)

Dim 1 Dim 2 Dim 3 Dim 4 Dim 5

PBA_A 0.5582867521 0.0171639564 0.024541407 0.001820207 0.005844092

PBA_P 0.5582867521 0.0171639564 0.024541407 0.001820207 0.005844092

Pottery_A 0.0159688340 0.4459009693 0.001668132 0.195277864 0.144358106

Pottery_P 0.0159688340 0.4459009693 0.001668132 0.195277864 0.144358106

Spondylus_A 0.0282893637 0.5209274111 0.014958092 0.003414009 0.258973005

Spondylus_P 0.0282893637 0.5209274111 0.014958092 0.003414009 0.258973005

Flaked_A 0.2741360199 0.0024544394 0.362992122 0.012313577 0.005605836

Flaked_P 0.2741360199 0.0024544394 0.362992122 0.012313577 0.005605836

Projectile_A 0.3902851366 0.0007793777 0.064698744 0.010281953 0.214537048

Projectile_P 0.3902851366 0.0007793777 0.064698744 0.010281953 0.214537048

Bone_A 0.3189037067 0.0150098586 0.239658114 0.094413021 0.020265203

Bone_P 0.3189037067 0.0150098586 0.239658114 0.094413021 0.020265203

female 0.5298799725 0.1039238917 0.016450916 0.007462245 0.000477699

male 0.5298799725 0.1039238917 0.016450916 0.007462245 0.000477699

high 0.1099075397 0.1004681794 0.361422251 0.105005266 0.029614788

low 0.1263279369 0.0948910148 0.125815720 0.328625554 0.203442349

medium 0.0002372598 0.2791219969 0.046225970 0.572530627 0.053554863

> # Contributions to the principal components

> (var$contrib)

Dim 1 Dim 2 Dim 3 Dim 4 Dim 5

PBA_A 5.036378430 0.256815569 0.45427245 0.04082627 0.14167762

PBA_P 19.261938559 0.982206913 1.73739288 0.15614258 0.54185477

Pottery_A 0.209767150 9.715062521 0.04496252 6.37786205 5.09599099

Pottery_P 0.485244491 22.473397638 0.10400970 14.75360860 11.78831650

Spondylus_A 0.210429786 6.426938727 0.22830538 0.06314028 5.17680418

Spondylus_P 1.020808324 31.177489994 1.10752399 0.30629752 25.11300750

Flaked_A 1.822223196 0.027060181 4.95095044 0.20350615 0.10013804

Flaked_P 10.109000111 0.150119577 27.46598699 1.12897462 0.55552769

Projectile_A 1.914828502 0.006342183 0.65132782 0.12542420 2.82861199

Projectile_P 15.071553369 0.049919117 5.12658029 0.98720983 22.26391372

Bone_A 2.574043729 0.200944382 3.96921299 1.89472455 0.43957249

Bone_P 11.305603828 0.882579245 17.43340605 8.32192745 1.93067135

female 10.482712993 3.410000994 0.66779350 0.36704856 0.02539649

male 12.579255592 4.092001192 0.80135220 0.44045827 0.03047579

high 3.652859417 5.538305625 24.64769272 8.67709573 2.64507079

low 4.258583668 5.305591216 8.70275187 27.54386920 18.43021269

medium 0.004768854 9.305224927 1.90647820 28.61188413 2.89275740

## *Data visualisation*

***
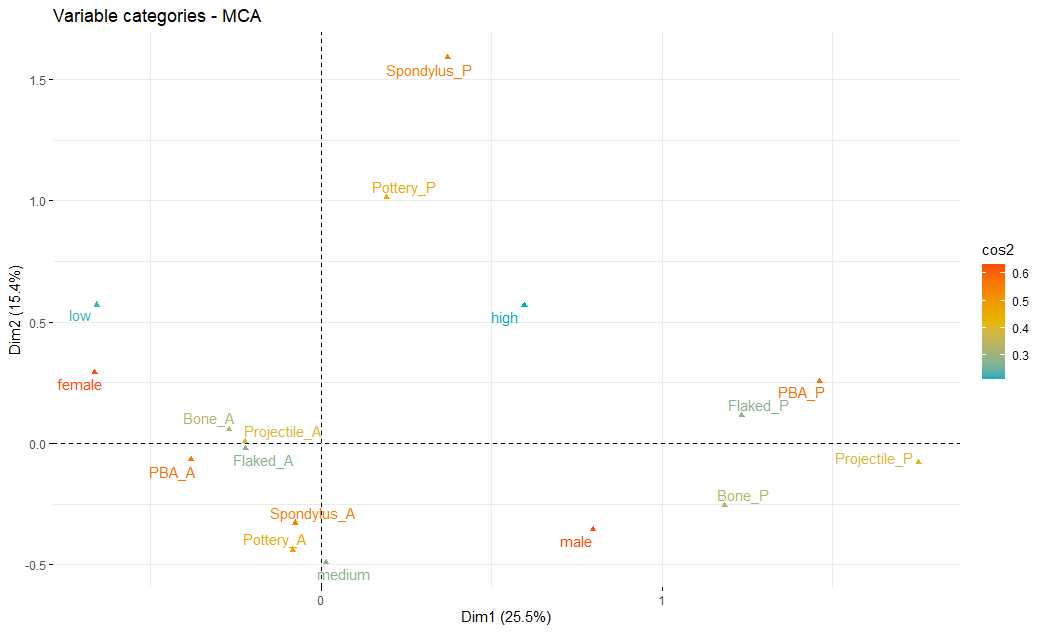
***We created another MCA plot showing the quality of representation of variable categories.


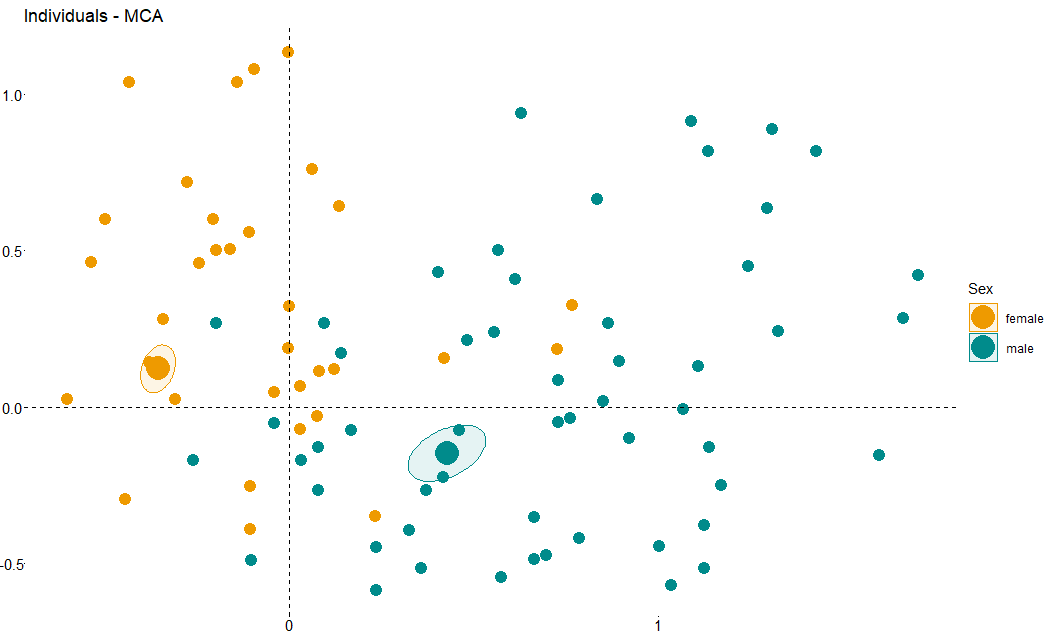
We created a plot colouring individuals by groups visualising “Sex”:

We created a plot colouring individuals by groups visualising all groups:


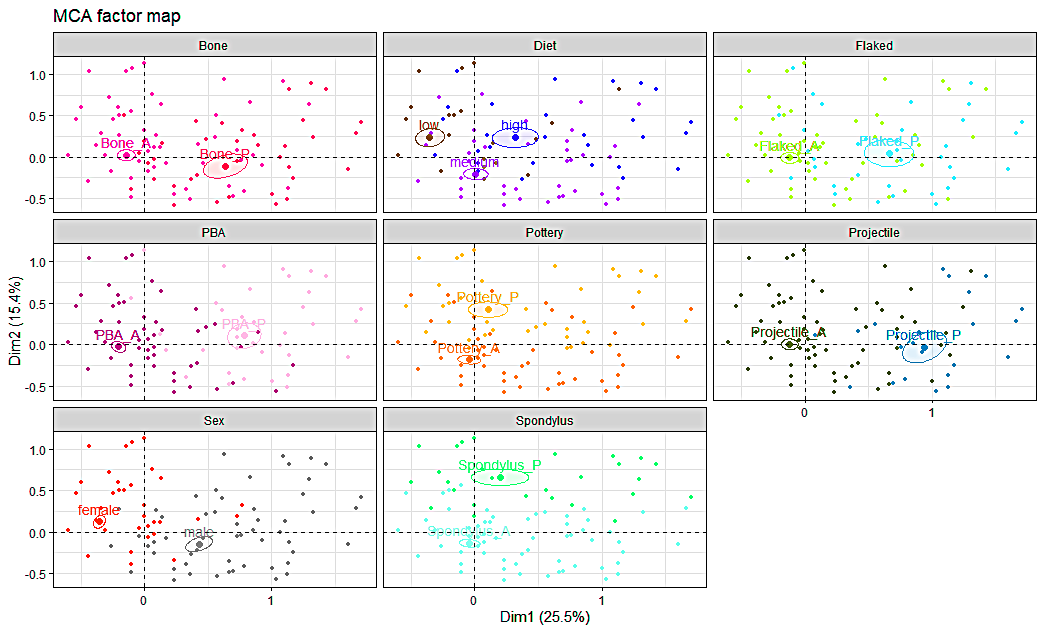


- 1. **MCA including the mobility data from eastern sites.**

In MCA terminology, our data contains active individuals (rows 1:84) and active variables (columns 1:9 = Site, PBA, Pottery, Spondylus, Flaked, Projectile, Bone, Sex, Diet, Mobility).

| **Site** | **PBA** | **Pottery** | **Spondylus** | **Flaked** | **Projectile** | **Bone** | **sex** | **diet** | **mobility** |
| --- | --- | --- | --- | --- | --- | --- | --- | --- | --- |
| Kleinhadersdorf | P | A | P | P | P | P | m | higher | local |
| Vedrovice | A | A | P | P | A | A | f | medium | local |
| Vedrovice | P | P | P | P | A | A | m | medium | local |
| Kleinhadersdorf | A | P | P | P | A | A | f | lower | local |
| Nitra | P | P | P | P | A | P | m | higher | local |
| Vedrovice | A | P | A | P | P | A | m | medium | local |
| Kleinhadersdorf | P | P | A | P | P | P | m | higher | local |
| Nitra | A | P | A | P | A | P | m | lower | local |
| Nitra | A | A | A | P | A | P | f | lower | local |
| Vedrovice | A | P | A | P | A | A | f | lower | local |
| Vedrovice | P | P | P | A | P | A | m | higher | local |
| Vedrovice | P | P | P | A | P | P | m | lower | local |
| Nitra | A | A | P | A | A | A | f | lower | non-local |
| Vedrovice | A | P | P | A | A | A | f | medium | local |
| Vedrovice | A | P | P | A | A | A | f | medium | non-local |
| Vedrovice | A | A | P | A | A | A | f | medium | local |
| Nitra | A | P | P | A | A | A | f | higher | local |
| Vedrovice | A | A | P | A | A | A | f | higher | local |
| Vedrovice | A | A | P | A | A | A | f | lower | non-local |
| Vedrovice | A | P | P | A | A | A | f | medium | non-local |
| Vedrovice | A | P | P | A | A | A | f | medium | non-local |
| Vedrovice | A | P | P | A | A | A | f | medium | non-local |
| Vedrovice | P | P | P | A | A | A | f | lower | local |
| Vedrovice | P | P | P | A | A | A | m | higher | local |
| Nitra | P | P | P | A | A | A | m | higher | local |
| Nitra | P | P | P | A | A | A | m | higher | local |
| Kleinhadersdorf | A | P | P | A | A | A | f | lower | non-local |
| Kleinhadersdorf | A | A | P | A | A | A | f | higher | local |
| Kleinhadersdorf | P | A | P | A | A | P | m | lower | local |
| Kleinhadersdorf | A | A | P | A | A | P | f | lower | non-local |
| Kleinhadersdorf | A | P | A | A | P | P | m | higher | local |
| Kleinhadersdorf | P | P | A | A | P | P | m | higher | local |
| Vedrovice | P | A | A | A | P | A | m | higher | local |
| Vedrovice | P | A | A | A | P | A | m | higher | local |
| Kleinhadersdorf | P | A | A | A | P | A | m | higher | local |
| Vedrovice | P | A | A | A | A | P | m | medium | local |
| Vedrovice | A | A | A | A | A | A | f | lower | local |
| Vedrovice | A | P | A | A | A | A | f | medium | local |
| Vedrovice | A | P | A | A | A | A | f | medium | non-local |
| Vedrovice | A | A | A | A | A | A | f | higher | local |
| Vedrovice | A | A | A | A | A | A | f | higher | local |
| Vedrovice | A | A | A | A | A | A | m | higher | local |
| Vedrovice | A | A | A | A | A | A | m | higher | local |
| Vedrovice | A | A | A | A | A | A | f | lower | local |
| Vedrovice | A | A | A | A | A | A | f | lower | local |
| Vedrovice | A | A | A | A | A | A | f | lower | local |
| Vedrovice | A | A | A | A | A | A | f | lower | local |
| Vedrovice | A | A | A | A | A | A | f | lower | non-local |
| Vedrovice | A | A | A | A | A | A | f | lower | non-local |
| Vedrovice | A | A | A | A | A | A | m | medium | local |
| Vedrovice | A | A | A | A | A | A | f | medium | non-local |
| Vedrovice | A | A | A | A | A | A | f | medium | non-local |
| Vedrovice | A | A | A | A | A | A | f | medium | non-local |
| Vedrovice | A | A | A | A | A | A | f | medium | non-local |
| Vedrovice | A | A | A | A | A | A | f | medium | non-local |
| Vedrovice | A | A | A | A | A | A | f | medium | non-local |
| Vedrovice | A | A | A | A | A | A | m | medium | non-local |
| Nitra | A | P | A | A | A | A | f | higher | local |
| Nitra | A | P | A | A | A | A | f | higher | local |
| Nitra | A | P | A | A | A | A | f | higher | local |
| Nitra | A | P | A | A | A | A | f | higher | local |
| Nitra | A | P | A | A | A | A | f | higher | non-local |
| Nitra | A | P | A | A | A | A | m | lower | local |
| Nitra | A | P | A | A | A | A | m | lower | local |
| Nitra | A | P | A | A | A | A | f | higher | local |
| Nitra | A | P | A | A | A | A | m | higher | local |
| Nitra | P | A | A | A | A | A | m | higher | local |
| Nitra | A | A | A | A | A | A | f | higher | local |
| Nitra | A | A | A | A | A | A | f | higher | local |
| Nitra | A | A | A | A | A | A | m | higher | local |
| Nitra | A | A | A | A | A | A | f | lower | local |
| Nitra | A | A | A | A | A | A | f | lower | local |
| Nitra | A | A | A | A | A | A | f | lower | non-local |
| Nitra | A | A | A | A | A | A | f | lower | non-local |
| Nitra | A | A | A | A | A | A | f | lower | non-local |
| Kleinhadersdorf | A | A | A | A | A | A | f | higher | local |
| Kleinhadersdorf | A | A | A | A | A | A | f | higher | local |
| Kleinhadersdorf | A | A | A | A | A | A | m | higher | local |
| Kleinhadersdorf | A | A | A | A | A | A | f | lower | local |
| Kleinhadersdorf | A | A | A | A | A | A | f | lower | local |
| Kleinhadersdorf | A | A | A | A | A | A | f | lower | local |
| Kleinhadersdorf | A | A | A | A | A | A | m | lower | local |
| Kleinhadersdorf | A | A | A | A | A | A | m | lower | local |
| Kleinhadersdorf | A | A | A | A | A | P | m | lower | local |

First, we select those variables that we want to be represented in the MCA (all but the category "site" and we use the function MCA()[FactoMiner package]

Second, we calcule and visualise the Eigenvalue

eigenvalue variance.percent cumulative.variance.percent

Dim.1 0.33525756 30.173180 30.17318

Dim.2 0.17235086 15.511578 45.68476

Dim.3 0.13747584 12.372825 58.05758

Dim.4 0.11245975 10.121377 68.17896

Dim.5 0.09674863 8.707377 76.88634

Dim.6 0.07074149 6.366734 83.25307

Dim.7 0.06189668 5.570702 88.82377

Dim.8 0.05498492 4.948642 93.77242

Dim.9 0.03858527 3.472674 97.24509


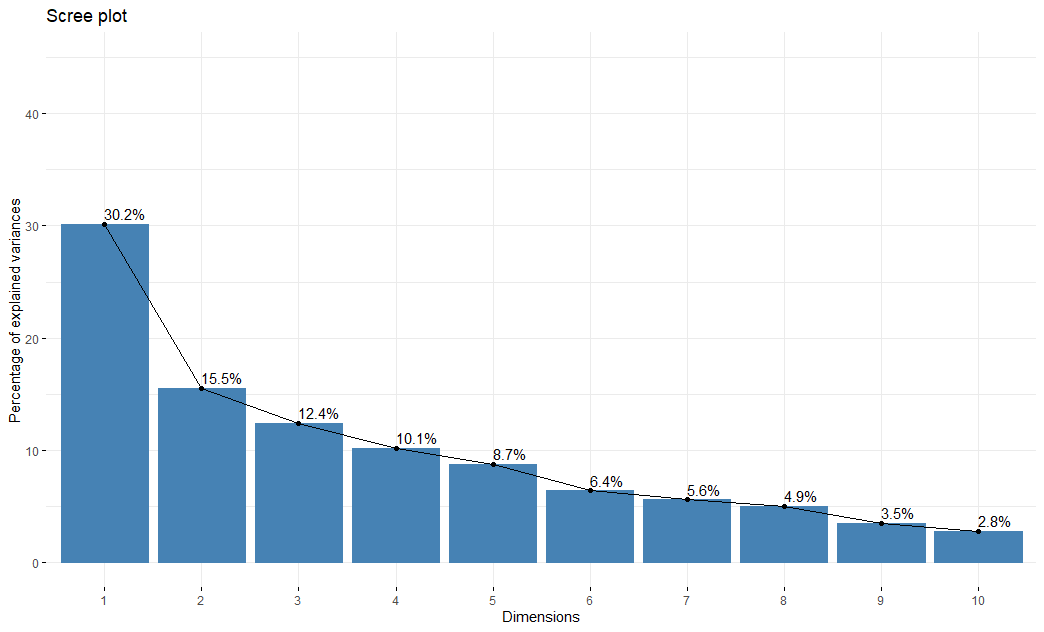
Dim.10 0.03061011 2.754910 100.00000


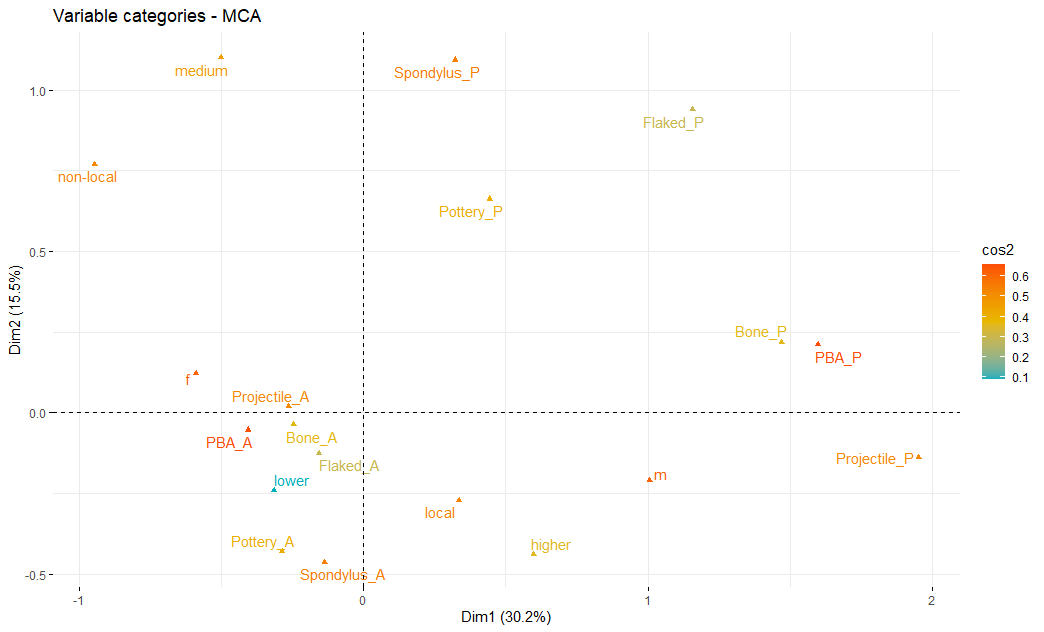

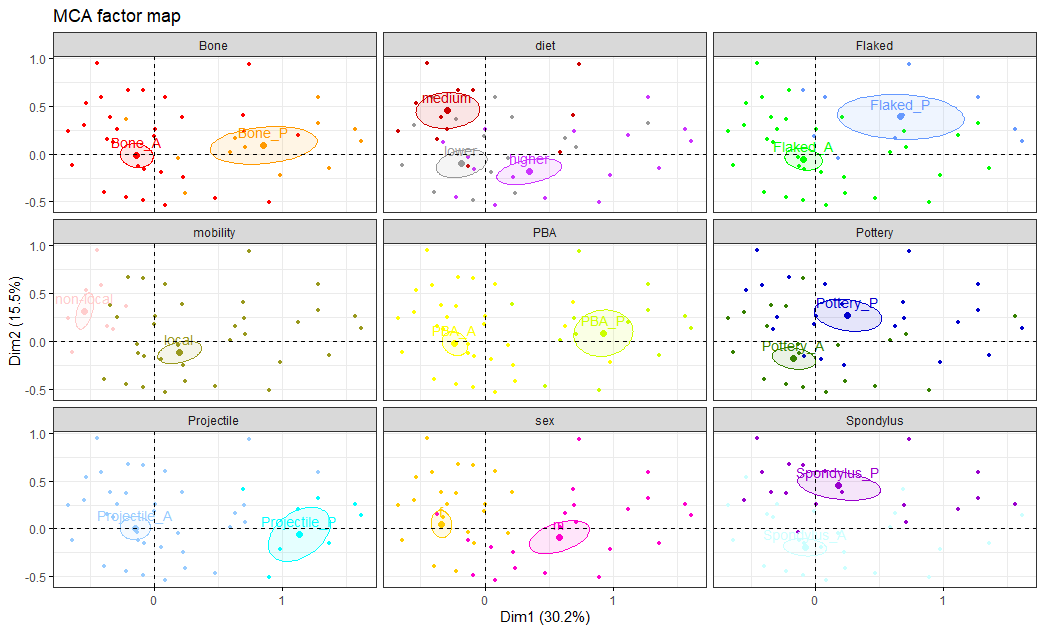
Multiple Correspondence Analysis Results for variables

===================================================

Name Description

1 "$coord" "Coordinates for categories"

2 "$cos2" "Cos2 for categories"

3 "$contrib" "contributions of categories"

> # Coordinates

> (var$coord)

Dim 1 Dim 2 Dim 3 Dim 4 Dim 5

PBA_A -0.4052912 -0.05348324 0.06094260 -0.08116819 0.16642026

PBA_P 1.5973240 0.21078688 -0.24018554 0.31989815 -0.65589162

Pottery_A -0.2862849 -0.42853234 0.20942235 0.34385036 -0.14469496

Pottery_P 0.4424403 0.66227725 -0.32365272 -0.53140510 0.22361949

Spondylus_A -0.1361218 -0.46304663 0.04165911 0.17421344 0.32393358

Spondylus_P 0.3212474 1.09279004 -0.09831550 -0.41114371 -0.76448324

Flaked_A -0.1562210 -0.12679198 -0.18359530 0.07497219 -0.18645690

Flaked_P 1.1560357 0.93826063 1.35860522 -0.55479422 1.37978105

Projectile_A -0.2636807 0.01876599 0.02303396 -0.13808187 -0.01500079

Projectile_P 1.9512373 -0.13886834 -0.17045133 1.02180585 0.11100587

Bone_A -0.2448884 -0.03650563 -0.22503938 -0.07893941 0.02401940

Bone_P 1.4693306 0.21903378 1.35023626 0.47363648 -0.14411638

f -0.5879095 0.12242376 0.03623052 -0.18039471 -0.01023097

m 1.0051356 -0.20930514 -0.06194251 0.30841677 0.01749166

higher 0.5984751 -0.44008975 -0.73289818 -0.25886963 0.05445605

lower -0.3138947 -0.24097976 0.98286509 -0.27077874 -0.48232294

medium -0.5009471 1.09966670 -0.31415889 0.84684193 0.65774808

local 0.3353174 -0.27260864 -0.01000211 -0.28690404 0.10141312

non-local -0.9449854 0.76826071 0.02818777 0.80854774 -0.28580061

> # Cos2: quality on the factore map

> (var$cos2)

Dim 1 Dim 2 Dim 3 Dim 4 Dim 5

PBA_A 0.64738133 0.011273565 0.0146375309 0.02596555 0.1091536539

PBA_P 0.64738133 0.011273565 0.0146375309 0.02596555 0.1091536539

Pottery_A 0.12666398 0.283807219 0.0677801147 0.18272384 0.0323566138

Pottery_P 0.12666398 0.283807219 0.0677801147 0.18272384 0.0323566138

Spondylus_A 0.04372877 0.506012740 0.0040957360 0.07162676 0.2476417904

Spondylus_P 0.04372877 0.506012740 0.0040957360 0.07162676 0.2476417904

Flaked_A 0.18059711 0.118963919 0.2494335319 0.04159414 0.2572696969

Flaked_P 0.18059711 0.118963919 0.2494335319 0.04159414 0.2572696969

Projectile_A 0.51450364 0.002606002 0.0039261699 0.14109286 0.0016651760

Projectile_P 0.51450364 0.002606002 0.0039261699 0.14109286 0.0016651760

Bone_A 0.35982206 0.007995966 0.3038563282 0.03738859 0.0034615885

Bone_P 0.35982206 0.007995966 0.3038563282 0.03738859 0.0034615885

f 0.59092873 0.025623924 0.0022442094 0.05563675 0.0001789566

m 0.59092873 0.025623924 0.0022442094 0.05563675 0.0001789566

higher 0.23175864 0.125321696 0.3475610086 0.04336167 0.0019188280

lower 0.05763069 0.033966199 0.5650327797 0.04288594 0.1360697743

medium 0.07842126 0.377895894 0.0308424408 0.22410664 0.1351976661

local 0.31687006 0.209434509 0.0002819373 0.23197561 0.0289839315

non-local 0.31687006 0.209434509 0.0002819373 0.23197561 0.0289839315

> # Contributions to the principal components

> (var$contrib)

Dim 1 Dim 2 Dim 3 Dim 4 Dim 5

PBA_A 4.3421890 0.14708722 0.239424496 0.5191915 2.537000561

PBA_P 17.1133330 0.57969670 0.943614192 2.0462254 9.998766915

Pottery_A 1.6491748 7.18790163 2.152127886 7.0923514 1.459857176

Pottery_P 2.5487247 11.10857524 3.326015824 10.9609067 2.256142909

Spondylus_A 0.4313272 9.70881456 0.098519862 2.1061845 8.464422002

Spondylus_P 1.0179322 22.91280237 0.232506875 4.9705953 19.976035924

Flaked_A 0.7125419 0.91301941 2.399974747 0.4892302 3.517401938

Flaked_P 5.2728101 6.75634363 17.759813126 3.6203034 26.028774340

Projectile_A 2.0299628 0.02000044 0.037776431 1.6595340 0.022766355

Projectile_P 15.0217246 0.14800325 0.279545587 12.2805514 0.168471028

Bone_A 1.7036041 0.07364053 3.508337514 0.5277174 0.056792377

Bone_P 10.2216243 0.44184320 21.050025085 3.1663041 0.340754262

f 7.2276386 0.60963806 0.066938644 2.0286381 0.007584778

m 12.3569304 1.04228442 0.114443487 3.4683167 0.012967524

higher 4.6634330 4.90525046 17.055052506 2.6011011 0.133794911

lower 1.2051172 1.38161666 28.813853852 2.6734491 9.859863386

medium 1.9802198 18.56166343 1.899243069 16.8700279 11.829939730

local 2.7504485 3.53619064 0.005967976 6.0026926 0.871792923

non-local 7.7512638 9.96562816 0.016818842 16.9166793 2.456870964

Data visualisation


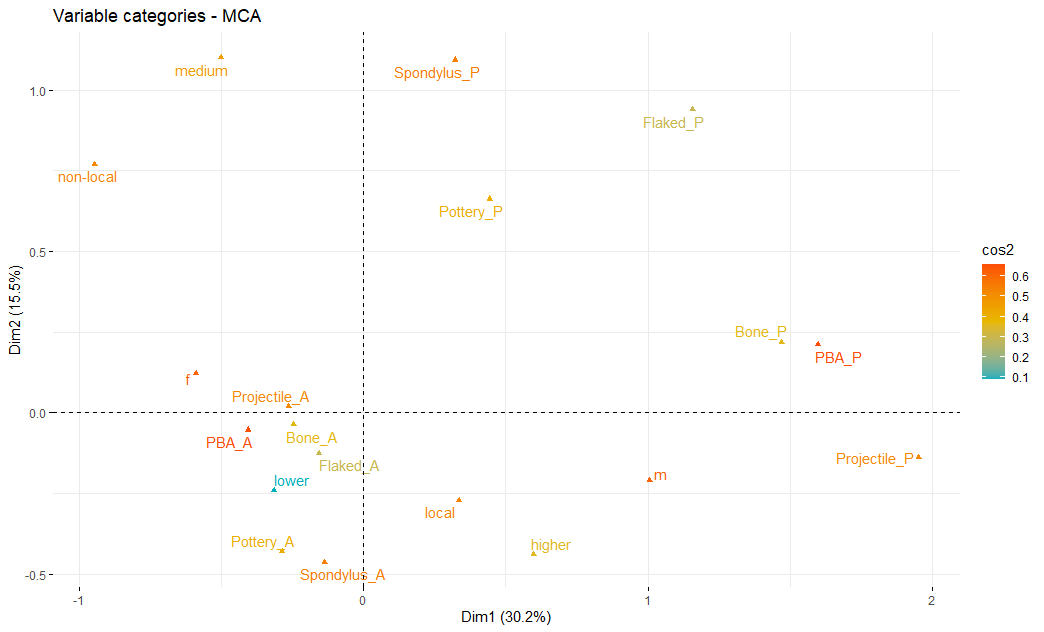
We created another MCA plot showing the quality of representation of variable categories.


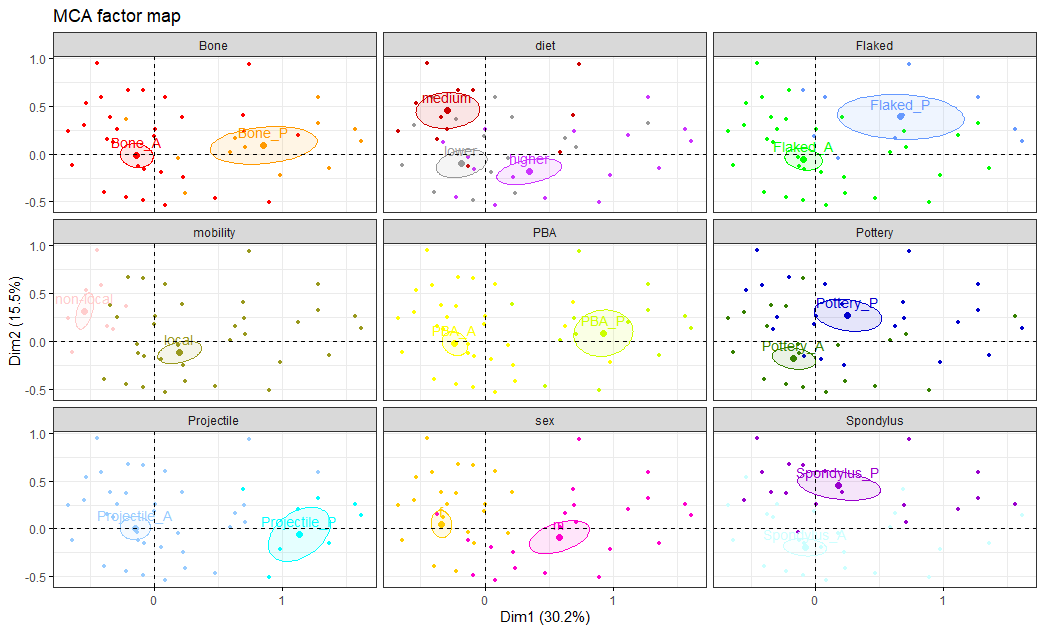
We created a plot colouring individuals by groups visualising all groups:

- 1. **MCA including the mobility data from Schwetzingen.**

In MCA terminology, our data contains active individuals (rows 1:93) and active variables (columns 1:9 = Site, PBA, Pottery, Spondylus, Flaked, Projectile, Bone, Sex, Diet, Mobility).

| **PBA** | **Pottery** | **Spondylus** | **Flaked** | **Projectile** | **Bone** | **sex** | **diet** | **mobility** |
| --- | --- | --- | --- | --- | --- | --- | --- | --- |
| A | A | P | A | A | A | f | lower | local |
| A | A | P | A | A | A | f | lower | non-local |
| A | A | P | A | A | A | f | medium | non-local |
| A | A | A | A | P | A | f | higher | local |
| A | A | A | P | P | A | m | higher | local |
| A | A | A | A | A | P | f | higher | non-local |
| A | A | A | A | A | P | m | higher | local |
| A | A | A | P | A | A | f | higher | non-local |
| A | P | A | A | A | A | f | higher | local |
| A | A | A | A | A | A | m | higher | local |
| A | A | A | A | A | A | f | higher | non-local |
| A | A | A | A | P | P | m | lower | local |
| A | A | A | P | A | A | m | lower | non-local |
| A | P | A | A | A | A | f | lower | local |
| A | P | A | A | A | A | f | lower | local |
| A | P | A | A | A | A | f | lower | local |
| A | A | A | A | A | A | f | lower | local |
| A | A | A | A | A | A | f | lower | local |
| A | A | A | A | A | A | f | lower | local |
| A | A | A | A | A | A | f | lower | local |
| A | A | A | A | A | A | f | lower | local |
| A | A | A | A | A | A | f | lower | non-local |
| A | A | A | A | A | P | f | medium | local |
| A | A | A | P | P | A | m | medium | non-local |
| A | A | A | A | P | A | m | medium | local |
| A | A | A | P | P | A | m | medium | non-local |
| A | A | A | P | P | A | m | medium | non-local |
| P | P | A | P | A | P | m | medium | local |
| A | A | A | A | A | P | m | medium | local |
| A | A | A | P | P | A | m | medium | local |
| P | A | A | A | P | P | m | medium | local |
| A | A | A | A | A | P | m | medium | local |
| A | A | A | A | A | P | m | medium | local |
| P | A | A | A | P | P | m | medium | local |
| A | P | A | A | A | P | f | medium | non-local |
| A | P | A | P | P | A | f | medium | local |
| A | P | A | P | P | A | m | medium | local |
| P | P | A | A | P | A | m | medium | local |
| P | A | A | A | P | A | m | medium | local |
| A | A | A | A | P | A | m | medium | non-local |
| A | A | A | A | P | A | m | medium | non-local |
| A | A | A | A | P | A | m | medium | non-local |
| P | P | A | P | A | A | m | medium | local |
| P | A | A | P | A | A | m | medium | local |
| A | A | A | P | A | A | f | medium | local |
| A | A | A | P | A | A | m | medium | non-local |
| A | P | A | A | A | A | m | medium | local |
| A | P | A | A | A | A | f | medium | local |
| A | P | A | A | A | A | f | medium | local |
| A | P | A | A | A | A | f | medium | local |
| A | P | A | A | A | A | f | medium | local |
| A | P | A | A | A | A | f | medium | local |
| A | P | A | A | A | A | f | medium | local |
| A | P | A | A | A | A | f | medium | non-local |
| A | P | A | A | A | A | f | medium | non-local |
| A | P | A | A | A | A | m | medium | non-local |
| P | A | A | A | A | A | m | medium | local |
| P | A | A | A | A | A | m | medium | local |
| P | A | A | A | A | A | m | medium | local |
| P | A | A | A | A | A | m | medium | non-local |
| A | A | A | A | A | A | f | medium | local |
| A | A | A | A | A | A | f | medium | local |
| A | A | A | A | A | A | f | medium | local |
| A | A | A | A | A | A | f | medium | local |
| A | A | A | A | A | A | f | medium | local |
| A | A | A | A | A | A | f | medium | local |
| A | A | A | A | A | A | f | medium | local |
| A | A | A | A | A | A | f | medium | local |
| A | A | A | A | A | A | f | medium | local |
| A | A | A | A | A | A | f | medium | local |
| A | A | A | A | A | A | f | medium | local |
| A | A | A | A | A | A | f | medium | local |
| A | A | A | A | A | A | m | medium | local |
| A | A | A | A | A | A | m | medium | local |
| A | A | A | A | A | A | m | medium | local |
| A | A | A | A | A | A | m | medium | local |
| A | A | A | A | A | A | m | medium | local |
| A | A | A | A | A | A | m | medium | local |
| A | A | A | A | A | A | f | medium | non-local |
| A | A | A | A | A | A | f | medium | non-local |
| A | A | A | A | A | A | f | medium | non-local |
| A | A | A | A | A | A | f | medium | non-local |
| A | A | A | A | A | A | f | medium | non-local |
| A | A | A | A | A | A | f | medium | non-local |
| A | A | A | A | A | A | f | medium | non-local |
| A | A | A | A | A | A | m | medium | non-local |
| A | A | A | A | A | A | m | medium | non-local |
| A | A | A | A | A | A | m | medium | non-local |
| A | A | A | A | A | A | m | medium | non-local |
| A | A | A | A | A | A | m | medium | non-local |
| A | A | A | A | A | A | m | medium | non-local |
| A | A | A | A | A | A | m | medium | non-local |
| A | A | A | A | A | A | m | medium | non-local |

First, we select those variables that we want to be represented in the MCA and we use the function MCA()[FactoMiner package]

Second, we calculate and visualise the Eigenvalue

eigenvalue variance.percent cumulative.variance.percent

Dim.1 0.23121443 20.809299 20.80930

Dim.2 0.15206642 13.685978 34.49528

Dim.3 0.12909087 11.618178 46.11346

Dim.4 0.12639995 11.375995 57.48945

Dim.5 0.12557626 11.301863 68.79131

Dim.6 0.08696397 7.826757 76.61807

Dim.7 0.08011057 7.209951 83.82802

Dim.8 0.06947190 6.252471 90.08049

Dim.9 0.06145108 5.530597 95.61109

**
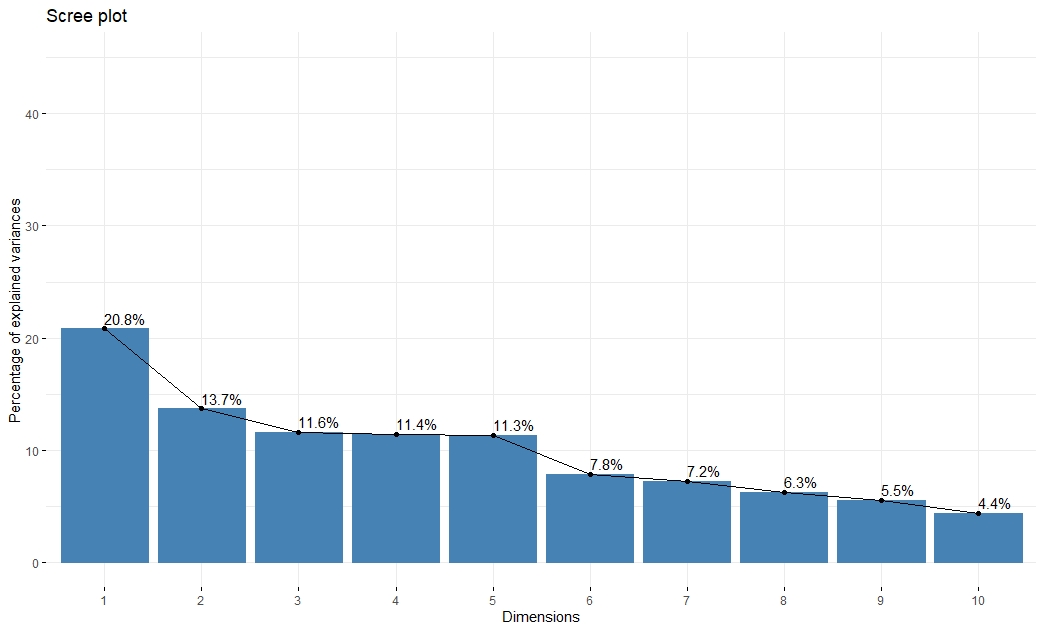
**Dim.10 0.04876566 4.388909 100.00000

Multiple Correspondence Analysis Results for variables

===================================================

Name Description

1 "$coord" "Coordinates for categories"

2 "$cos2" "Cos2 for categories"

3 "$contrib" "contributions of categories"

> # Coordinates

> (var$coord)

Dim 1 Dim 2 Dim 3 Dim 4 Dim 5

PBA_A -0.21367387 -0.135240391 -0.03749250 0.11751092 -0.083338201

PBA_P 1.59284155 1.008155640 0.27948954 -0.87599051 0.621248409

Pottery_A 0.03808976 -0.295796657 0.30410854 -0.08820600 -0.004874728

Pottery_P -0.13902761 1.079657798 -1.10999615 0.32195189 0.017792757

Spondylus_A 0.07149977 0.044517181 -0.03939718 0.02894638 -0.114558509

Spondylus_P -2.14499297 -1.335515436 1.18191548 -0.86839129 3.436755280

Flaked_A -0.20653575 0.094068612 0.18192944 -0.15627164 -0.141093658

Flaked_P 1.16545170 -0.530815738 -1.02660187 0.88181854 0.796171354

Projectile_A -0.29775302 0.084282646 0.04000497 -0.11598877 -0.160024192

Projectile_P 1.33113113 -0.376793006 -0.17884573 0.51853805 0.715402271

Bone_A -0.10943795 -0.119336460 -0.24293000 -0.05591352 0.019340417

Bone_P 0.81581019 0.889599066 1.81093275 0.41680991 -0.144174017

f -0.75969701 0.173618165 -0.13711441 0.21218453 -0.054982054

m 0.81034348 -0.185192709 0.14625537 -0.22633016 0.058647525

higher 0.06424116 -0.409620148 0.94967673 2.63690131 -0.654692751

lower -1.20130607 0.293492471 0.27648274 0.24345461 1.579965818

medium 0.20976458 -0.007478346 -0.15544013 -0.33694612 -0.212527967

local 0.04690791 0.591396940 0.06042222 0.10053492 0.133664854

non-local -0.08139902 -1.026247631 -0.10485033 -0.17445765 -0.231947835

> # Cos2: quality on the factore map

> (var$cos2)

Dim 1 Dim 2 Dim 3 Dim 4 Dim 5

PBA_A 0.3403486126 0.1363433628 0.010478761 0.102938453 5.177372e-02

PBA_P 0.3403486126 0.1363433628 0.010478761 0.102938453 5.177372e-02

Pottery_A 0.0052955279 0.3193591671 0.337559304 0.028398088 8.673485e-05

Pottery_P 0.0052955279 0.3193591671 0.337559304 0.028398088 8.673485e-05

Spondylus_A 0.1533664948 0.0594533826 0.046564140 0.025136781 3.937096e-01

Spondylus_P 0.1533664948 0.0594533826 0.046564140 0.025136781 3.937096e-01

Flaked_A 0.2407074364 0.0499330996 0.186769108 0.137803230 1.123347e-01

Flaked_P 0.2407074364 0.0499330996 0.186769108 0.137803230 1.123347e-01

Projectile_A 0.3963483094 0.0317571116 0.007154717 0.060144593 1.144817e-01

Projectile_P 0.3963483094 0.0317571116 0.007154717 0.060144593 1.144817e-01

Bone_A 0.0892805975 0.1061616034 0.439929897 0.023305311 2.788386e-03

Bone_P 0.0892805975 0.1061616034 0.439929897 0.023305311 2.788386e-03

f 0.6156155173 0.0321528184 0.020053718 0.048023758 3.224561e-03

m 0.6156155173 0.0321528184 0.020053718 0.048023758 3.224561e-03

higher 0.0003884166 0.0157918744 0.084883378 0.654423388 4.034095e-02

lower 0.2345096453 0.0139973975 0.012421940 0.009631399 4.056474e-01

medium 0.1508611833 0.0001917451 0.082839889 0.389254920 1.548622e-01

local 0.0038182582 0.6069197083 0.006335290 0.017539085 3.100327e-02

non-local 0.0038182582 0.6069197083 0.006335290 0.017539085 3.100327e-02

> # Contributions to the principal components

> (var$contrib)

Dim 1 Dim 2 Dim 3 Dim 4 Dim 5

PBA_A 1.93453356 1.178332657 0.1066797 1.0702814 0.541837553

PBA_P 14.42106834 8.783934349 0.7952484 7.9784610 4.039152668

Pottery_A 0.05472665 5.018230553 6.2482600 0.5368423 0.001650405

Pottery_P 0.19975227 18.316541520 22.8061488 1.9594744 0.006023979

Spondylus_A 0.23774497 0.140132559 0.1292861 0.0712785 1.123735811

Spondylus_P 7.13234922 4.203976755 3.8785832 2.1383550 33.712074324

Flaked_A 1.74131420 0.549234474 2.4199818 1.8235391 1.496266938

Flaked_P 9.82598726 3.099251674 13.6556113 10.2899705 8.443220579

Projectile_A 3.48165336 0.424161400 0.1125694 0.9664361 1.851620089

Projectile_P 15.56503854 1.896250964 0.5032515 4.3205379 8.277830985

Bone_A 0.50746883 0.917490090 4.4787330 0.2423122 0.029181830

Bone_P 3.78294945 6.839471583 33.3869188 1.8063271 0.217537278

f 14.31468086 1.136770484 0.8351922 2.0426610 0.138054373

m 15.26899292 1.212555183 0.8908717 2.1788384 0.147257998

higher 0.01705989 1.054614737 6.6776029 52.5781593 3.262361288

lower 9.69416249 0.879788963 0.9197259 0.7282944 30.874912718

medium 1.63702911 0.003163624 1.6100443 7.7264686 3.094080980

local 0.06708166 16.212552115 0.1993538 0.5636552 1.002890397

non-local 0.11640641 28.133546317 0.3459374 0.9781075 1.740309807

Data visualisation

We created another MCA plot showing the quality of representation of variable categories:

**
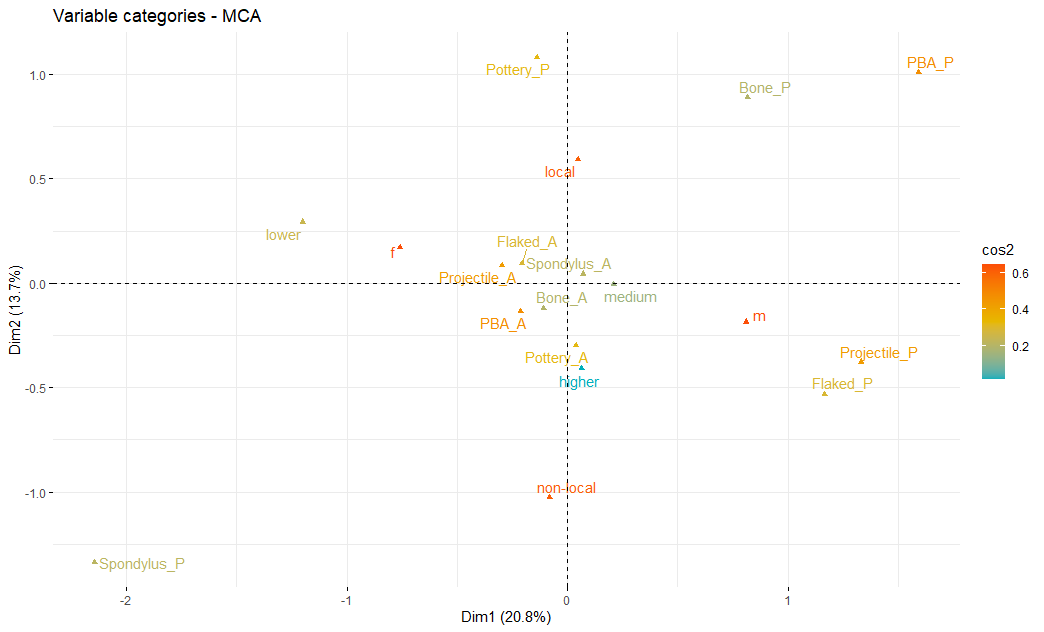
**

**
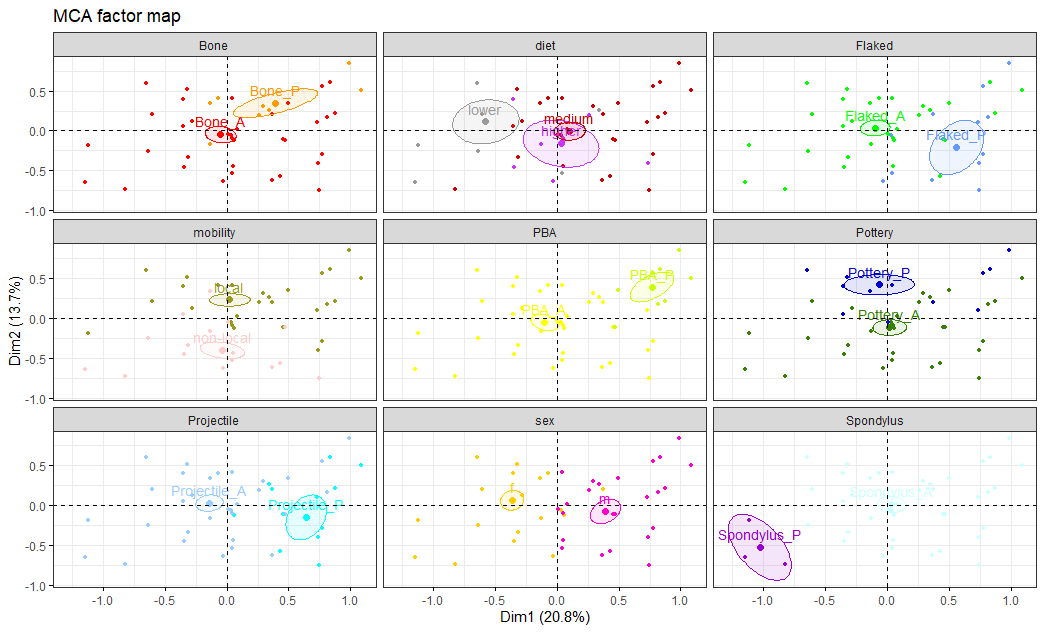
**We created a plot, colouring individuals by groups visualising all groups:

**References**

1. R Core Team R. A language and environment for statistical computing. R Foundation for Statistical Computing (Vienna, Austria, 2013). <http://www.R-project.org/>

2. Hervé A, Williams LJ. Principal Component Analysis. Inc. WIREs Comp Stat. 2010; 2: 433–59.

3. Husson F, Le S, Pagès J, Exploratory Multivariate Analysis by Example Using R. Florida: Boca Raton; 2017.

4. Wickham H. ggplot2: Elegant Graphics for Data Analysis. New York: Springer-Verlag; 2016. <https://ggplot2.tidyverse.org>.
